# MR2G: A novel framework for causal network inference using GWAS summary data

**DOI:** 10.1371/journal.pgen.1012144

**Published:** 2026-05-26

**Authors:** Zhaotong Lin, Wei Pan, Haoran Xue

**Affiliations:** 1 Department of Statistics, Florida State University, Tallahassee, Florida, United States of America; 2 Division of Biostatistics and Health Data Science, School of Public Health, University of Minnesota, Minneapolis, Minnesota, United States of America; 3 Department of Biostatistics, City University of Hong Kong, Hong Kong, China; The University of Chicago, UNITED STATES OF AMERICA

## Abstract

Inferring a causal network among multiple traits is essential for unraveling complex biological relationships and informing interventions. Mendelian randomization (MR) has emerged as a powerful tool for causal inference, utilizing genetic variants as instrumental variables (IVs) to estimate causal effects. However, when the directions of causal relationships among traits are unknown, reconstructing the underlying causal network becomes challenging. In particular, the presence of cycles or feedback loops, which are common in biological systems, poses additional challenges for causal network inference, and remains largely under-studied with standard MR approaches and existing IV-based network inference methods. To address these issues, we introduce MR2G, a new statistical framework that enables robust inference of causal networks, including those with cycles, directly from GWAS summary statistics. MR2G is built on a formally defined recursive causal graph model that rigorously links direct causal effects to (univariable) MR estimands. It recovers a biologically interpretable causal network from pairwise MR effect estimates, while incorporating a network-informed IV screening strategy to reduce pleiotropic bias and improve robustness. Through realistic simulations, MR2G demonstrates superior accuracy and robustness in recovering complex causal structures, including those involving feedback loops. We apply MR2G to GWAS summary statistics for six complex diseases and nine cardiometabolic risk factors. MR2G not only recovers well-established causal pathways but also uncovers multiple feedback relationships, highlighting its utility in disentangling complex and biologically plausible causal networks from large-scale genetic data.

## Author summary

Unraveling how complex traits causally influence each others is essential for understanding disease mechanisms and guiding effective interventions. However, inferring such a causal network from observational data remains challenging due to hidden confounding, and most existing methods assume that the underlying network has no feedback loops, which is often unrealistic in biological systems. In this work, we developed MR2G, a statistical framework that infers a causal network of multiple traits directly from GWAS summary statistics, while allowing for cycles and feedback loops. Our approach is based on a recursive causal model that formally links direct causal effects to Mendelian randomization estimands, and incorporates a novel instrumental variable screening strategy to reduce bias from horizontal pleiotropy. We applied MR2G to 15 complex traits including cardiometabolic risk factors and diseases, and identified biologically supported causal pathways, such as the direct effects of blood pressure and LDL cholesterol on coronary artery disease, and a bidirectional relationship between atrial fibrillation and coronary artery disease.

## Introduction

Understanding how complex human traits causally influence one another is critical for uncovering disease mechanisms and identifying effective intervention targets. For example, traits like blood pressure, body mass index (BMI), and lipid levels are known to be interrelated and collectively influence the risk of cardiometabolic diseases. Modeling these interdependencies as a causal network allows researchers to move beyond isolated pairwise effects and instead capture the broader architecture of trait–trait relationships. However, inferring such networks from observational data is challenging due to confounding, reverse causation, and the presence of feedback loops.

Mendelian randomization (MR) provides a powerful tool for causal inference in the presence of unmeasured confounders by leveraging genetic variants, mostly single-nucleotide polymorphisms (SNPs), as instrumental variables (IVs) [1]. Traditional MR methods, both univariable (i.e., one exposure) [2–6] and multivariable (i.e., multiple exposures) [7–11], assume the directions of effects are known from exposures to outcomes. When causal direction between a pair of traits is unclear, bidirectional MR methods have been proposed to infer causal effects in both directions [12–15], but they do not account for complex causal relationships among a set of interrelated traits.

The MR framework has considerable potential to be extended to causal network inference among traits [16,17], and several methods have been proposed for this purpose. Early efforts focused on mediation or two-step MR frameworks to test whether one trait mediates the effect of another on an outcome [18,19], but these approaches are limited to networks with only three (or a few more) traits, and require pre-specified directionality. More recent methods combine genetic instruments with structural learning algorithms to infer larger networks. For example, [20] developed BN-GWAS to construct a network that links multiple genes to an outcome. [21] introduced a linear structural equation model (SEM)-based method to infer a causal protein network without cycles. [22] proposed MrDAG, a Bayesian approach to estimate causal relationships among multiple exposures and outcomes under the assumption of no feedback loops. However, these approaches typically assume a directed acyclic graph (DAG) structure and/or often require the causal directions between exposures and outcomes to be specified a priori, limiting their applicability in settings where feedback or cyclic relationships are biologically plausible. For example, in type 2 diabetes (T2D), insulin resistance leads to elevated blood glucose, which in turn can exacerbate insulin resistance, forming a positive feedback cycle. Graph-MRcML [23] relaxed the DAG assumption by first estimating a total-(causal-)effect network using bidirectional MR, then followed by network deconvolution to reconstruct the direct-(causal-)effect network [24]. However, Graph-MRcML lacks a formal causal model, and the equivalence between the total-(causal-)effect network and the MR-estimated network may break down for cyclic networks (as to be discussed later).

To address the limitations of existing approaches, we introduce **MR2G**, a new framework for inferring the causal network among multiple complex traits using MR with GWAS summary statistics. MR2G is built upon a recursive model, which captures the propagation of causal effects through the (potentially cyclic) direct-(causal-)effect network of interest denoted by **G**. Under appropriate assumptions, this dynamic system converges to an equilibrium state, allowing us to define the MR-(causal-)effect network denoted by Θ which can be estimated by MR, and derive an analytical expression linking **G** and Θ. With this insight, MR2G first estimates Θ using bidirectional MR, and then directly reconstructs the direct-effect network **G** from the estimated Θ, without relying on structural learning or the heuristic network deconvolution algorithm. To further improve robustness, we introduce a novel IV screening strategy **ScreenAug** when performing MR. Unlike standard Steiger-type filtering [25] commonly used in bidirectional MR analysis, ScreenAug incorporates the inferred network structure to further exclude SNPs that may exert correlated pleiotropic effects on the outcome through alternative pathways after conditioning on the exposure.

We apply MR2G to investigate causal relationships among nine cardiometabolic risk factors and six diseases, including four major cardiometabolic diseases. MR2G successfully identifies multiple plausible and biologically meaningful causal relationships, including the independent effects of systolic blood pressure (SBP) and low-density lipoprotein (LDL) cholesterol on coronary artery disease (CAD), while traits such as BMI and triglycerides (TG) appear to exert more indirect effects. It also reveals BMI and height as direct contributors to atrial fibrillation (AF), a bidirectional causal link between AF and CAD, and AF as a major upstream driver of stroke. These findings highlight the ability of MR2G to robustly recover interpretable causal networks from large-scale genetic data, even in the presence of complex interdependencies and feedback loops.

## Results

### Method overview

**Fig 1:**
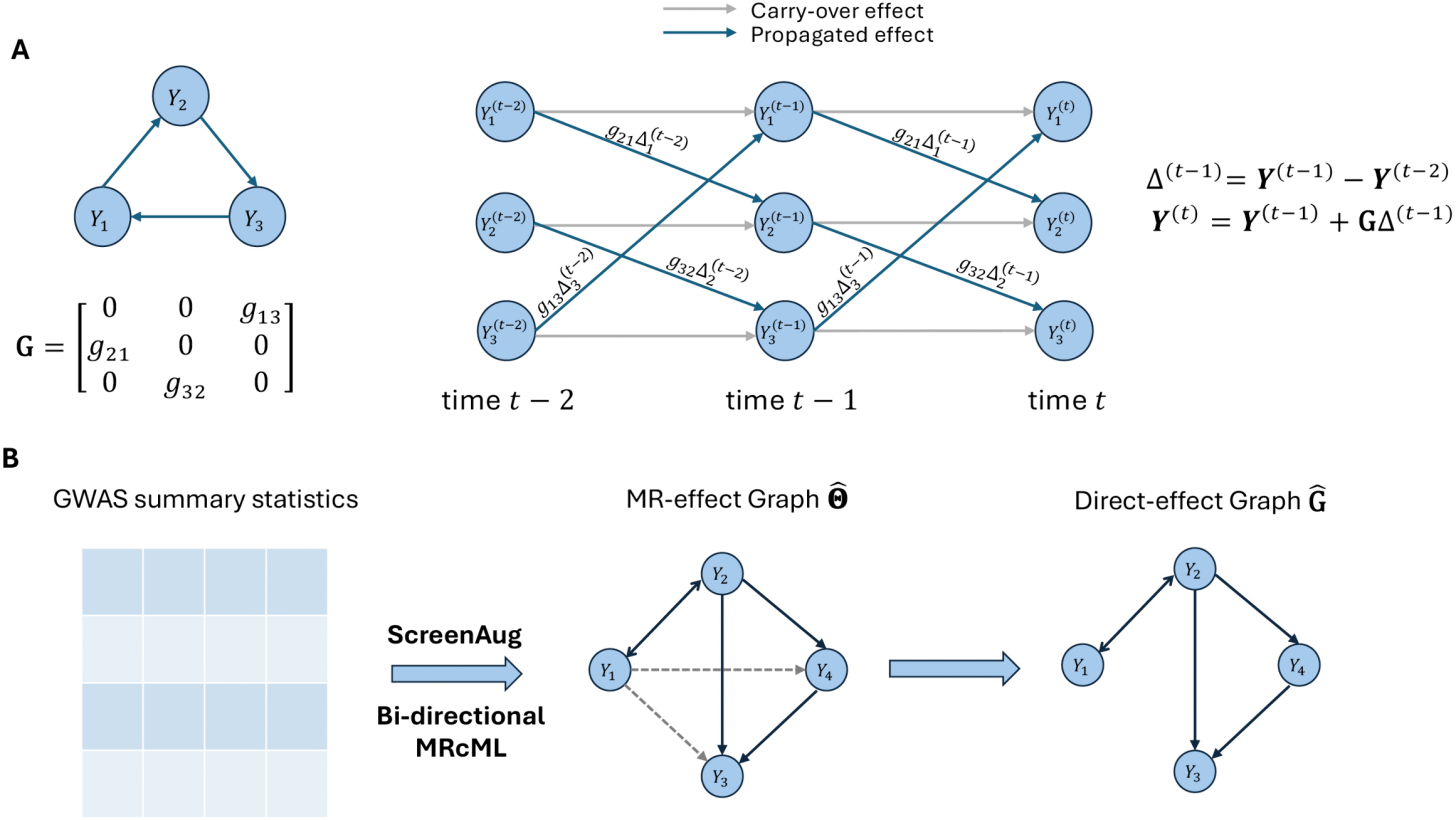
Illustrations of the proposed cyclic causal network model and the MR2G method. Panel **A** shows that in a causal network of three traits, values of traits evolve over time and can be decomposed as the sum of values from the previous time point (i.e., carry-over effects) and changes due to causal effects (i.e., propagated effects). In panel **B**, for a causal network of four traits, MR2G first estimates an MR-effect network $\hat{\Theta }$ and then constructs the direct-effect network $\hat{G}$ from the estimated MR-effect network.

**MR2G** is a two-stage framework for inferring direct-(causal-)effect networks from GWAS summary statistics. It is based on a recursive model that allows for feedback loops and cycles, capturing how traits influence each other over time. Specifically, we consider a dynamic system where trait values evolve over discrete time points: (1) At the initial time point, the trait values are determined by SNPs (with their direct effects on traits denoted by matrix Γ) and random errors; (2) At each subsequent time point, the trait values are updated by combining carry-over effects from the previous time point together with effects propagated through the direct-(causal-)effect network denoted by matrix **G** (Fig 1A). Under mild conditions on the spectral radius of **G**, this system converges to an equilibrium state, at which the total effects of SNPs on traits (denoted by matrix **B**) are jointly determined by **G** and Γ. For each ordered pair of traits, with SNPs being their valid IVs (see Definition 1), we can define an estimable MR-(causal-)effect using total SNP effects from **B**, and eventually derive an analytical relationship between **G** and the MR-(causal-)effect network Θ as shown in Eq (7). More details about the model are in the section Causal network model with cyclic structures.

MR2G operates in two stages (Fig 1B). In Stage 1, we obtain the estimated MR-effect network $\hat{\Theta }$ via a robust bidirectional (univariable) MR method across all trait pairs. Specifically, we adopt our previously developed method, (univariable) MRcML [6], which accommodates invalid IVs exhibiting both correlated and uncorrelated pleiotropy under a plurality condition [6,12,23]. In Stage 2, based on $\hat{\Theta }$, MR2G estimates the direct-effect network as $\hat{G}$ using the connection between **G** and Θ in Eq (7). To enable robust statistical inference on the entries of $\hat{G}$, we employ a data perturbation (equivalent to parametric bootstrapping) procedure based on a matrix normal distribution that captures both the linkage disequilibrium (LD) structure among the genetic instruments and the correlation structure of GWAS summary statistics. Further implementation details are provided in the section MR2G: estimating causal networks from GWAS summary statistics.

**Fig 2:**
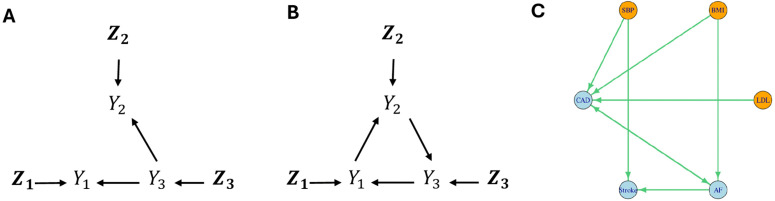
True direct-effect networks used in simulation studies. In panels **A** and **B**, *Y*_1_, *Y*_2_, *Y*_3_ are three traits, and $Z_{1},Z_{2},Z_{3}$ represent three sets of SNPs exerting direct effects on the corresponding traits. Panel **C** is estimated based on real data application.

To further enhance robustness in MR estimation and network inference, we develop a novel IV selection strategy, **ScreenAug**, which leverages the estimated causal network to refine instrument selection (see the section IV screening procedure). It is applied after an initial candidate IV set being selected with **ScreenPair** and **ScreenMax**. Briefly, ScreenPair implements Steiger’s filtering by selecting a SNP as an instrument for the trait with which it has a stronger marginal association than that with the other trait [25]. However, this pairwise screening rule can lead to overlapping IVs across trait pairs, increasing the risk of using SNPs with horizontal pleiotropy. As demonstrated in our simulations, this issue becomes more pronounced with increasing GWAS sample sizes, as weaker effects become more likely to pass the selection threshold. To mitigate this issue, ScreenMax globally prunes the candidate IV set by retaining only the strongest SNP–trait association across all traits and assigns the SNP as an IV exclusively for that trait. For example, in Fig 2A, SNPs in the set ***Z***_3_ are associated with both traits *Y*_1_ and *Y*_2_, yet are not valid IVs for either of them due to the shared confounder *Y*_3_. While ScreenPair may incorrectly use ***Z***_3_ to infer either $Y_{1}\to Y_{2}$ or $Y_{2}\to Y_{1}$ based on marginal association strengths, ScreenMax will only use them as IVs for *Y*_3_, as associations of ***Z***_3_ with *Y*_3_ are stronger than with *Y*_1_ and *Y*_2_. However, ScreenMax can also exclude valid instruments. To balance stringency and sensitivity, ScreenAug augments the ScreenMax-filtered set by reintroducing a subset of excluded SNPs if they are deemed valid based on the estimated causal network. Specifically, a SNP is re-included as an IV for a trait pair only if the inferred network indicates no direct path through which the SNP could influence the outcome after conditioning on the exposure.

In summary, MR2G combines a theoretically sound cyclic causal network model, robust (univariable) MR estimation via MRcML with a network-aware IV screening procedure, and a closed-form graph recovery algorithm to enable robust and interpretable causal network inference from GWAS summary data while allowing for feedback loops and cycles, invalid instruments, and complex trait interdependencies.

### ScreenAug outperforms other IV selection strategies by leveraging the inferred network structure

To evaluate the impact of IV screening on causal network estimation, we first conducted simulation studies under simple settings with three traits (Fig 2A and Fig 2B). We compared three IV selection strategies, ScreenPair, ScreenMax, and the proposed ScreenAug (see the sections **Method overview** and **IV screening procedure**), each applied within the MR2G framework.

**Fig 3:**
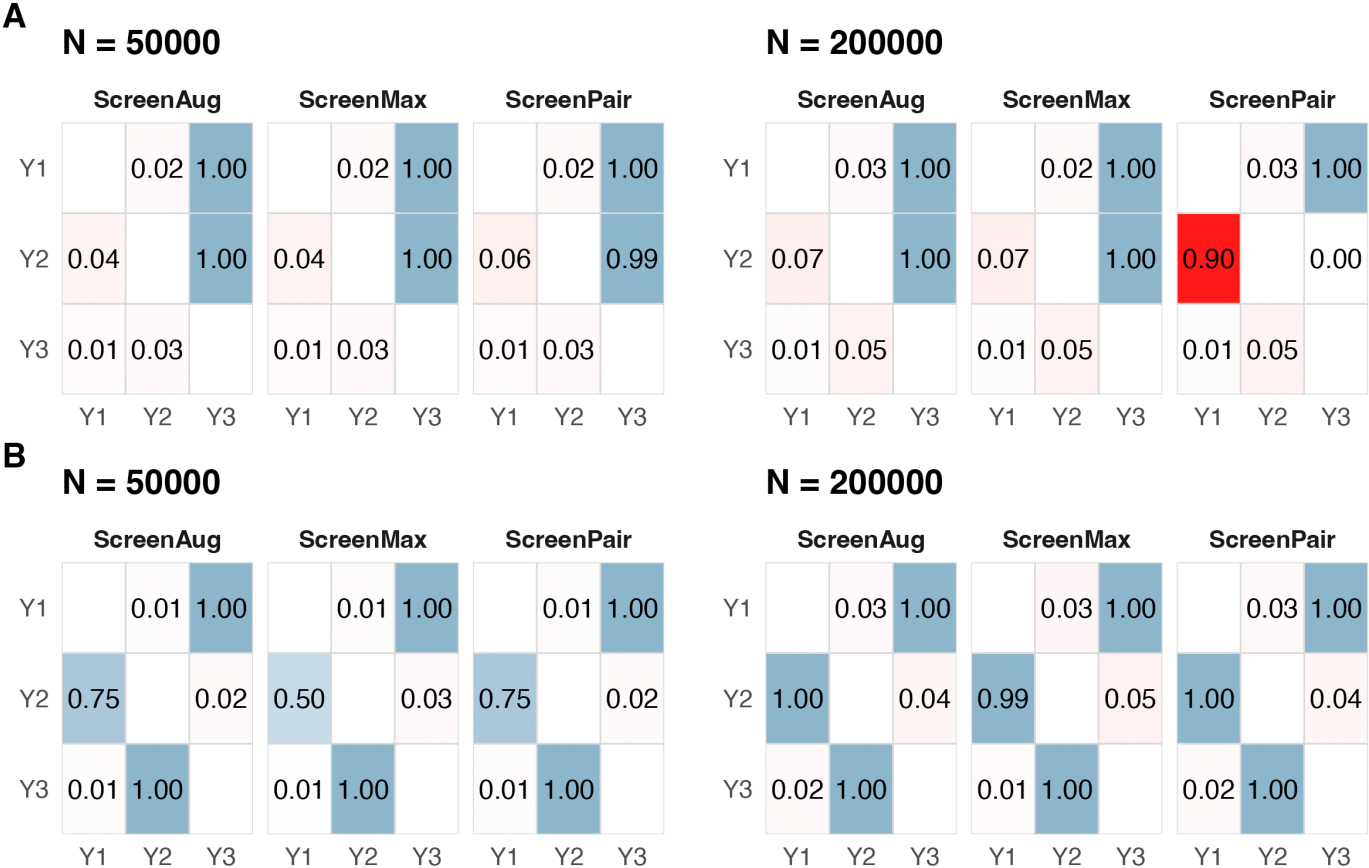
Proportions of rejections by MR2G across three IV-selection approaches in (A) the acyclic setting (Fig 2A) and (B) the cyclic setting (Fig 2B). Each cell reports the rejection proportion for the effect from the column trait to the row trait. Within each panel, the left column corresponds to *N* = 50,000 and the right column to *N* = 200,000. Power is indicated in blue and type I error in red.

This simulation involved three traits and a set of 30 SNPs, among which 8 SNPs directly affected *Y*_1_ ($|Z_{1}|=8$), 7 SNPs directly affected *Y*_2_ ($|Z_{2}|=7$), and 15 SNPs directly affected *Y*_3_ ($|Z_{3}|=15$). In the first scenario of Fig 2A, when the sample size was *N* = 50000, MR2G coupled with all three IV screening methods worked similarly well, with well-controlled type-I error (Fig 3A left panel) and almost no bias (Figure A in S1 Appendix). However, it might seem surprising that when the sample size increased to *N* = 200 000 (Fig 3A right panel), the performance of ScreenPair dropped dramatically with highly inflated type-I error and substantial bias (Figure A in S1 Appendix). This is because when the sample size increased, SNPs in ***Z***_3_ reached the significance threshold for marginal associations with *Y*_1_ (and *Y*_2_). When performing a bi-directional MR analysis on *Y*_1_ and *Y*_2_, ScreenPair incorrectly selected the SNPs in ***Z***_3_ as IVs for *Y*_1_, due to their stronger correlations with *Y*_1_ than that with *Y*_2_ (as $g_{13}=0.2>g_{23}=0.1$). However, these SNPs in ***Z***_3_ were all invalid IVs for *Y*_1_ in this scenario as they exhibited pleiotropic effects on *Y*_2_. Furthermore, since ***Z***_3_ constituted the largest group ($\left|Z_{3}|>|Z_{1}\right|$), the plurality assumption required by MRcML was violated, leading to poor estimation of the MR effect $Y_{1}\to Y_{2}$. It is also noted that, although ScreenPair could correctly estimate the MR effect of $Y_{3}\to Y_{2}$, the spurious effect of $Y_{1}\to Y_{2}$ effectively accounted for the true $Y_{3}\to Y_{2}$ edge through the indirect pathway $Y_{3}\to Y_{1}\to Y_{2}$, causing the estimated direct effect of $Y_{3}\to Y_{2}$ to shrink toward zero and loss of power (Figure A in S1 Appendix). In contrast, ScreenMax demonstrated robust performance by selecting SNPs as IVs only for the trait with which they had the strongest marginal association. As a result, only SNPs in ***Z***_1_ were used as IVs for *Y*_1_ in the analysis, successfully recovering the true direct-effect network. ScreenAug performed similarly to ScreenMax, as the initial direct-effect network estimated using ScreenMax ensured that SNPs in ***Z***_3_ were not added into the IV set for *Y*_1_ when inferring $Y_{1}\to Y_{2}$. This is because, after removing *Y*_1_, these SNPs still had a path to *Y*_2_ via *Y*_3_.

In the second scenario Fig 2B, all three screening methods performed well with well-controlled type-I error (Fig 3B) and almost unbiased estimates (Figure B in S1 Appendix). Unlike in Fig 2A, ScreenPair did not have the same issue when the sample size was large. This is because, in this scenario, SNPs in ***Z***_3_ became valid IVs for *Y*_1_ when inferring $Y_{1}\to Y_{2}$. Notably, ScreenAug achieved higher power than ScreenMax for inferring $Y_{1}\to Y_{2}$ when *N* = 50 000 (0.75 v.s. 0.50). This is because ScreenMax’s conservative global pruning assigned SNPs in ***Z***_3_ exclusively to *Y*_3_, even though they were also valid IVs for *Y*_1_ in this setting. ScreenAug recovered these valid IVs by verifying that, after removing *Y*_1_ from the reference graph, no alternative path existed from these SNPs to *Y*_2_, thereby augmenting the IV set and improving power. This illustrates the advantage of ScreenAug that it retains the robustness of ScreenMax while recovering valid IVs that ScreenMax unnecessarily excludes.

Furthermore, we also applied the Graph-MRcML approach [23] for comparison, which used ScreenPair for IV selection and the network deconvolution algorithm for estimating direct-(causal-)effect network **G**. The results are provided in Figure C in S1 Appendix. Overall, Graph-MRcML had performance similar to the performance of ScreenPair in Fig 3. It is worth noting that, in scenario Fig 2B, although all selected IVs were valid, it yielded a slightly biased estimate of the direct-effect network. This is because Graph-MRcML [23] approximated the off-diagonal elements of the total-effect network $G_{tot}$ as defined in Eq (9) by pairwise MR. As shown in the section **Relationship with network deconvolution**, this approximation became inaccurate in the presence of cycles ($Y_{1}\to Y_{2}\to Y_{3}\to Y_{1}$ in this case), leading to a biased estimate of the direct-effect network.

We conducted several additional sensitivity analyses for the three-trait simulation. First, we varied the AR(1) residual correlation to assess robustness to confounding strength (Section 3.3.2 in S1 Appendix). Second, we reduced the direct-effect sizes and controlled per-trait SNP heritability at different levels to evaluate the performance (Section 3.3.3 in S1 Appendix). Third, we introduced an explicit latent confounder *U* with direct effects on all three traits to examine robustness beyond correlated residuals (Section 3.3.4 in S1 Appendix). Across all settings, the main conclusions remained consistent that ScreenMax and ScreenAug maintained well-controlled type-I error, while ScreenPair exhibited inflated false positives in the acyclic setting when indirect associations became detectable. Additionally, we compared MR2G with conventional univariable MR methods (IVW, MR-Egger, and MR-PRESSO) to illustrate the limitations of standard approaches in the network setting (Section 3.3.5 in S1 Appendix).

### MR2G accurately reconstructs complex causal networks in simulations designed to mimic real-world GWAS data

**Fig 4:**
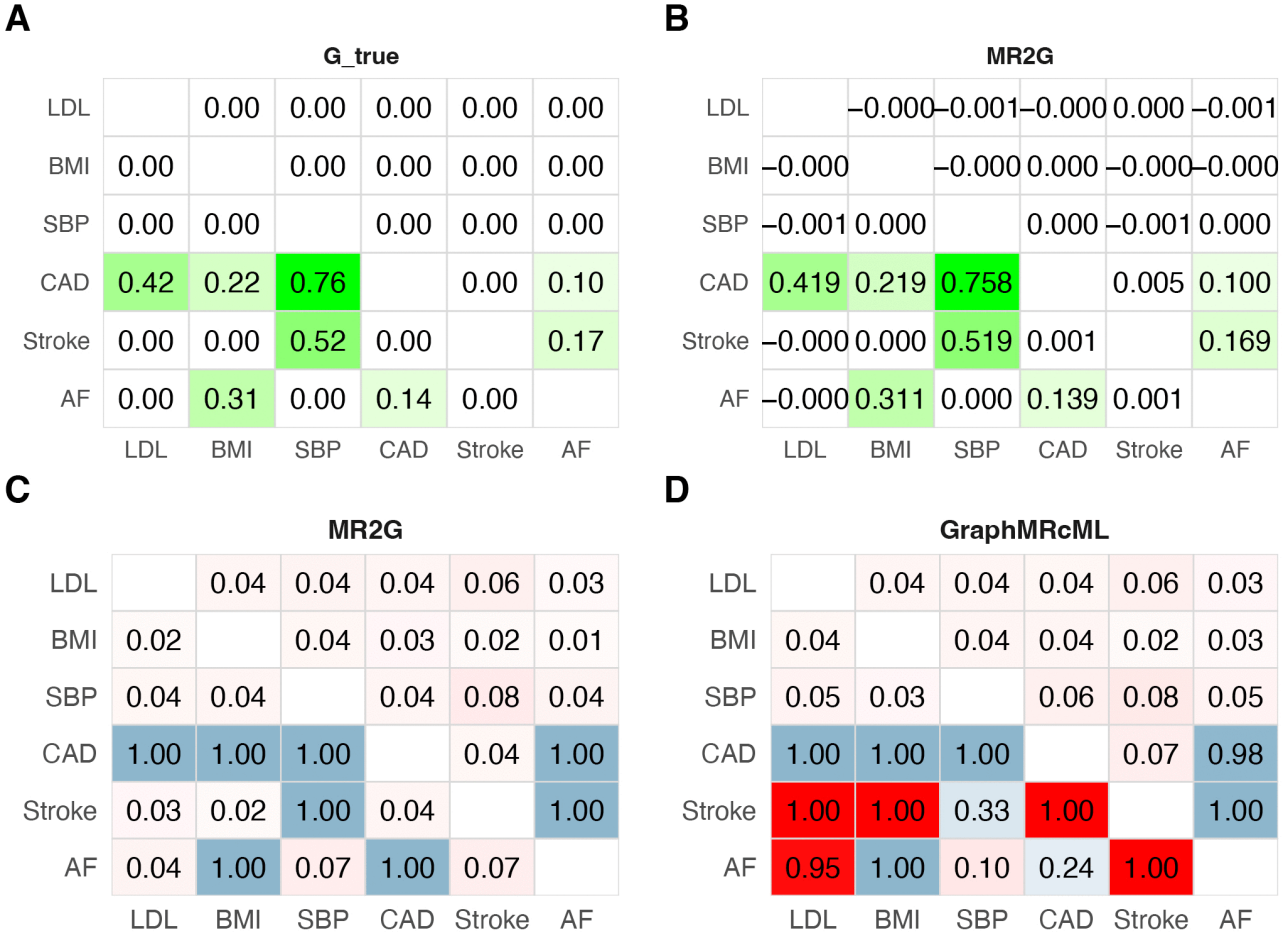
Simulation results based on real data application in Fig 2C. Panel **A** shows the true direct-effect network **G** used in the simulation. Panel **B** shows the mean of $\hat{G}$ estimated by MR2G across 200 replicates (values are rounded to three decimal places). Panels **C** and **D** show the rejection rate of MR2G and Graph-MRcML, respectively. Power is indicated by a blue colour scale; type I error is indicated by a red colour scale.

To evaluate the performance of MR2G under realistic genetic architectures, we designed a simulation using six traits and an underlying direct-effect network estimated from real GWAS data (Fig 2C, Fig 4A). We directly simulated the GWAS summary statistics of 1056 SNPs across 392 LD blocks that closely mimicked the real data analysis and preserved realistic LD patterns (see the section Simulation based on real GWAS summary data for details).

MR2G successfully recovered the direct-effect network, achieving well-controlled type-I error (Fig 4C) and nearly unbiased estimates (Fig 4B). In contrast, Graph-MRcML, which is also a two-stage approach that allows for cycles, exhibited substantially inflated type-I error for several pathways involving Stroke (Fig 4D). The unsatisfactory performance of Graph-MRcML is primarily attributable to its IV screening step, where ScreenPair tends to include invalid instruments with pleiotropic effects, as discussed next.

We examined the other two IV screening strategies used by MR2G. ScreenPair exhibited highly inflated type-I error rates in some pairs, particularly for the pathways involving Stroke (Figure D in S1 Appendix). This inflation was primarily due to the poor estimation of the MR effects for the pathways CAD → Stroke and Stroke → AF (Figure E in S1 Appendix). ScreenMax produced results similar to ScreenAug but with a slightly higher root mean squared error (Table A in S1 Appendix). We further investigated the poor performance of ScreenPair. When inferring the causal relationship CAD → Stroke, ScreenPair selected many SNPs that had a direct effect on SBP, which in turn had an indirect effect on Stroke that did not mediate through CAD. A similar issue arose when inferring the relationship Stroke → AF. These findings are consistent with the simpler setting in the previous section and further confirm the robustness of ScreenAug in the presence of realistic polygenic architectures and complex causal network structure with indirect pathways.

In summary, the proposed method MR2G reliably recovered complex causal networks with accurate effect estimates and well-controlled false positives. These results highlight the effectiveness of combining a theoretically sound network recovery framework with robust MR estimation and the ScreenAug strategy, which improves IV selection in the presence of horizontal pleiotropy and trait interdependencies. To further assess robustness, we repeated this simulation under weaker causal effects by scaling the direct-effect matrix **G** in Fig 4A by λ∈{0.1,0.25,0.5} (Section 3.4.1 in S1 Appendix). The results were consistent with the above conclusions, with ScreenMax and ScreenAug maintaining well-controlled type-I error across all scaling factors.

### MR2G reveals biologically plausible network among 15 traits with large-scale GWAS summary data

**Fig 5:**
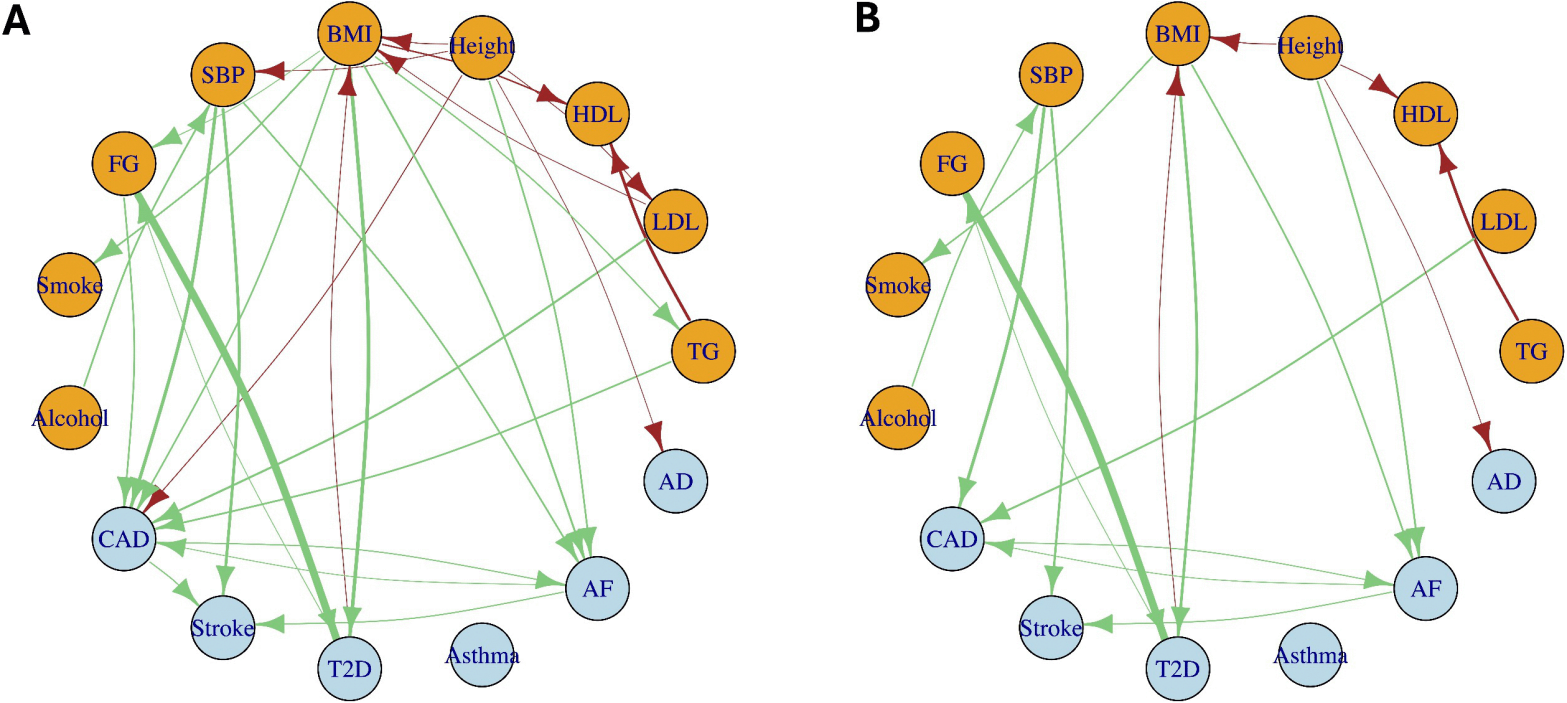
Networks among 15 complex traits inferred by MR2G with GWAS summary data. Panel **A** is the estimated MR-effect network $\hat{\Theta }$, and panel **B** is the estimated direct-effect network $\hat{𝐆}$. Edge color indicates the sign of an estimated effect (green for positive and red for negative), and edge width is proportional to its magnitude.

We applied MR2G to infer a causal network among 15 complex traits, including 9 common risk factors — body mass index (BMI), height, systolic blood pressure (SBP), low-density lipoprotein (LDL) cholesterol, high-density lipoprotein (HDL) cholesterol, triglycerides (TG), fasting glucose (FG), smoking, and alcohol drinking — and six major diseases: coronary artery disease (CAD), stroke, atrial fibrillation (AF), type 2 diabetes (T2D), Alzheimer’s disease (AD), and asthma. Fig 5A and Fig 5B show the MR-effect and direct-effect networks inferred by MR2G (with 1000 data perturbations/bootstrap samples) using IVs selected by ScreenAug. Edges represent significant effects passing Bonferroni corrected *p*-value threshold, with green and red indicating positive and negative effects, respectively. The estimated effect sizes with their standard errors are shown in the Figures G and H in S1 Appendix.

First, we discuss several key findings on the refined causal landscape of the three cardiovascular diseases. For CAD, the estimated MR-effect network indicated multiple risk factors, including BMI, height, FG, SBP, and lipid traits (LDL and TG). However, after accounting for potential indirect effects mediated by other traits, only SBP and LDL remained as independent direct causal factors. This is consistent with findings from large-scale studies such as the Cholesterol Treatment Trialists’ Collaboration [26], which demonstrated LDL’s direct causal role in CAD, and several large studies [27,28], which highlighted SBP as a robust, independent predictor of CAD risk. Other factors including BMI, height, FG, and TG often exert their effects indirectly through mediators such as hypertension, dyslipidemia, or insulin resistance [29–31]. The direct-effect network also highlighted the role of SBP as a key determinant of stroke. Furthermore, while the MR-effect network suggested a marginally significant Alcohol → Stroke relationship with an effect size of 0.26 (standard error = 0.09, *p*-value = 0.0018), this association completely disappeared in the direct-effect network with an effect size of 0.03 and *p*-value greater than 0.7, indicating that alcohol’s influence on stroke risk is largely mediated through increases in SBP. The direct-effect graph also provided important insights into AF and its relationship with CAD and stroke. BMI and height were identified as strong independent risk factors for AF, consistent with the well-established roles of obesity in atrial remodeling [32] and the emerging evidence that taller individuals have a greater predisposition to AF due to larger atrial dimensions and prolonged conduction pathways [33,34]. Furthermore, a bidirectional causal relationship between CAD and AF was observed, suggesting a complex interplay between these conditions [35]. This supports previous studies indicating that atherosclerosis may contribute to AF via systemic inflammation and endothelial dysfunction [36], while AF may exacerbate CAD risk through altered hemodynamics and increased thromboembolic burden [37,38]. Additionally, AF was confirmed as a direct causal risk factor for stroke, reinforcing its role as a major contributor to cardioembolic stroke [39,40]. Interestingly, the direct CAD → stroke relationship was only marginally significant in the direct-effect graph, suggesting that effect of CAD on stroke is partially mediated through AF or other vascular mechanisms rather than a direct causal pathway.

Beyond findings related to the three cardiovascular diseases, the FG ↔ T2D ↔ BMI pathway provided insights into the metabolic interdependence of diabetes and obesity. The bidirectional relationship between fasting glucose and T2D is expected, as elevated FG levels contribute to diabetes onset, while T2D exacerbates glycemic dysregulation through insulin resistance and beta-cell dysfunction. Interestingly, while obesity is a well-established risk factor for T2D (BMI → T2D), a negative causal effect of T2D on BMI was discovered, suggesting that the onset of diabetes may lead to weight loss. This is consistent with clinical observations where unintended weight loss is one of the symptoms of diabetes [41], often resulting from dehydration, metabolic inefficiency, increased glucose excretion, and muscle breakdown [42,43]. Further studies are warranted to explore the underlying mechanisms and potential clinical implications of this bidirectional relationship.

The direct-effect network also revealed the causal relationship between height and AD, suggesting that taller individuals may have a reduced risk of AD. This finding aligns with recent observational studies [44] and MR studies [45,46] reporting height as a protective factor of AD. Moreover, the effect sizes of height → AD remained similar between the MR-effect and direct-effect graphs, suggesting that the effect was not strongly influenced by mediation through other pathways considered in this analysis. Lastly, as a negative control, asthma was included to assess the specificity of the inferred causal effects. Consistent with what is expected, no significant causal relationship was identified between asthma and key metabolic or cardiovascular traits.

We also evaluated the impact of different IV screening methods, ScreenAug, ScreenMax, and ScreenPair, on the inferred direct-effect network structure (Figure A in S1 Appendix). The estimated networks were broadly consistent across all IV screening approaches, with only minor differences due to *p*-values falling slightly below the Bonferroni-adjusted threshold. For example, ScreenPair failed to detect the bidirectional relationship CAD ↔ AF after Bonferroni correction, despite these associations being nearly significant. ScreenAug and ScreenMax obtained similar direct-effect networks, but ScreenAug provided more precise causal effect estimates with smaller standard errors in over 75% of the estimated causal effects.

## Discussion

Understanding how complex traits influence one another is essential for uncovering disease mechanisms and designing effective interventions. In this study, we introduce MR2G, a novel statistical framework that enables robust inference of direct-(causal-)effect networks among multiple traits from GWAS summary statistics, even in the presence of feedback loops/cycles and horizontal pleiotropy of SNPs. MR2G consists of three key components: (i) a recursive dynamic model that accommodates cyclic effects among traits, (ii) a closed-form graph reconstruction algorithm based on pairwise MR estimates, and (iii) a new IV selection strategy, ScreenAug, which uses the estimated network structure to mitigate bias from correlated pleiotropy.

Many existing approaches for causal network inference from GWAS data rely on structural equation models or Bayesian frameworks that assume a DAG structure. While these methods have enabled progress in disentangling trait relationships, they are fundamentally limited in settings where feedback loops and bidirectional influences are biologically plausible. In contrast, MR2G is built upon a recursive causal model that naturally accommodates cyclic networks and provides a theoretically sound link between direct effects and marginal MR effects. Compared to our previous Graph-MRcML framework [23], which also allows for feedback loops, MR2G replaces the heuristic network deconvolution step with a closed-form graph recovery algorithm, improving both the theoretical validity and computational efficiency. Additionally, MR2G extends Steiger’s filtering used in Graph-MRcML to ScreenAug for IV selection, which leverages the estimated causal structure to exclude instruments with potential pleiotropic effects, leading to more robust causal estimates.

It is also important to distinguish MR2G from conventional MR methods. Univariable MR methods such as IVW [47], MR-Egger [2], and MR-PRESSO [48], along with MRcML [6], estimate the pairwise MR effect $\theta_{ij}$, which captures both direct and indirect causal effects, but cannot disentangle the two. MR2G addresses this by recovering the direct-effect network **G** from the MR-effect network Θ via Eq (7). We have further shown that the resulting direct effects are the same as those from multivariable MR [7], which estimates the effect of each exposure on an outcome conditional on other exposures. However, unlike MVMR, MR2G does not require a pre-specified exposure–outcome direction and recovers the *entire* direct-effect network simultaneously. There may also be other technical challenges with application of MVMR, such as the exacerbated weak IV bias with multiple correlated exposures as often encountered [10]. MR2G can be regarded as another way to achieve the goal of MVMR (*and beyond*) through applying simpler univariable MR.

Simulation studies demonstrate that MR2G achieved well-calibrated type-I error control and unbiased estimation, even in challenging scenarios with dense horizontal pleiotropy and complex indirect pathways. The proposed ScreenAug procedure outperformed alternative IV screening strategies, offering greater robustness and improved precision in causal effect estimates. Although the benefits of ScreenAug were more modest in real data analyses with current GWAS sample sizes, simulation studies suggest that its advantages will become increasingly pronounced as sample sizes grow and weaker GWAS effects become more detectable. A sensitivity analysis with five traits further demonstrated that ScreenAug is robust to moderate misspecification of the reference graph used for IV augmentation, provided that edges involved in confounder-like motifs are captured (Section 3.5 in S1 Appendix). When applied to 15 complex traits, MR2G recovered biologically supported relationships, such as the direct effects of SBP and LDL cholesterol on CAD, the feedback loop between AF and CAD, and feedback loops among T2D, FG, and BMI.

The proposed MR2G framework still has certain limitations, and there are several promising extensions could be pursued in future work. First, although the recursive model guarantees convergence under a commonly adopted spectral radius condition, this constraint is not enforced during estimation, making it possible that the spectral radius of the estimated causal network $\hat{G}$ is greater than 1. Second, although ScreenAug empirically enhanced IV selection, its performance depends on the accuracy of the inferred direct-effect graph and thus inherits assumptions required by MRcML, such as the plurality condition. In practice, other robust UVMR methods, such as MR-APSS [49], can also be used in the MR2G framework, while their performance need to be further evaluated. In addition, ScreenAug cannot fully eliminate invalid IVs with (correlated) horizontal pleiotropy mediated by traits outside the analyzed network. Third, when the number of available IVs is limited, the significance threshold for IV selection could be relaxed (e.g., from 5e-8 to 5e-6) to include more candidate IVs. While this strategy increases the number of IVs, it may also introduce more weak IVs; some existing methods, such as MR-RAPS [50] (and likely MRcML [10] as we are currently studying), are robust to weak IVs, and thus can be used. Fourth, MR2G could be extended to incorporate correlated SNPs as IVs following existing methods such as *cis*-MRcML [51], which can increase the number of usable IVs (especially for molecular traits) and improve statistical efficiency. Fifth, while MR2G is built on a recursive causal model, Algorithm 1 is algebraically equivalent to the formula of [52]. In principle, MR2G can be extended to leverage interventional data (e.g., Perturb-seq) by using interventions instead of SNPs as IVs to infer gene regulatory networks, as in recent works [52,53]. Finally, future extensions to high-dimensional traits through penalization or regularization techniques will further enhance the utility of the MR2G framework.

In summary, MR2G provides a powerful and robust tool for elucidating causal relationships among complex traits using GWAS summary data. As biobank-scale datasets continue to grow, we expect that MR2G will facilitate more accurate construction of causal networks, enabling deeper understanding of the biological mechanisms underlying human traits and diseases.

## Materials and methods

### Causal network model with cyclic structures

#### Recursive model.

Suppose we have *T* traits of interest denoted as $Y={\left(Y_{1},\cdots ,Y_{T}\right)}^{𝖳}$, and *S* SNPs as IVs denoted as $Z={\left(Z_{1},\cdots ,Z_{S}\right)}^{𝖳}$. To accommodate potential feedback loops/cyclic causal relationships among traits, we model the system dynamically, allowing trait values to evolve over time. Denote t=1,2,⋯ as a series of discrete time points, the value of trait *Y*_*i*_ at time *t* is denoted as $Y_{i}^{\left(t\right)}$, and $Y^{\left(t\right)}={\left(Y_{1}^{\left(t\right)},\cdots ,Y_{T}^{\left(t\right)}\right)}^{𝖳}$. For the convenience of notation, we denote ***Y***^(0)^ as a vector of 0’s with length *T*. We adopt the following recursive model


$$\begin{array}{c} Y^{\left(1\right)}=\Gamma Z+\epsilon , \end{array}$$ (1)

$$\begin{array}{c} Y^{\left(t\right)}=Y^{\left(t-1\right)}+G\left(Y^{\left(t-1\right)}-Y^{\left(t-2\right)}\right),\text{ for }t\geq 2. \end{array}$$ (2)

**Fig 6:**
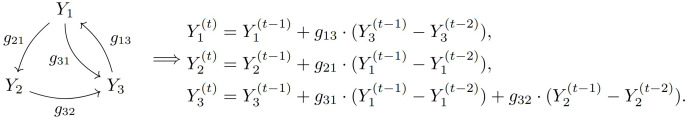
An illustrative example for the recursive model with three traits. The figure on the left shows the causal model with cycle $Y_{1}\to Y_{2}\to Y_{3}\to Y_{1}$ and bidirectional causal effects between *Y*_1_ and *Y*_3_, effect sizes are marked on the directed edges. As shown by the equations on the right, values of traits at time *t* are recursively determined by values at times *t* − 1 and *t* − 2.

Eq (1) is the initialization of the model at *t* = 1. Here Γ is a *T* × *S* matrix, with its element $\gamma_{ik}$ in the *i*^*th*^ row and *k*^*th*^ column being the direct effect from *Z*_*k*_ to *Y*_*i*_. The random errors $\epsilon ={\left(\epsilon_{1},\cdots ,\epsilon_{T}\right)}^{𝖳}$ are correlated, and their correlations account for unmeasured confounders. Eq (2) represents a second-order difference equation, which describes the recursive process. It consists of two components: a carry-over term ***Y***^(*t*−1)^ and a propagated change $G\left(Y^{\left(t-1\right)}-Y^{\left(t-2\right)}\right)$, which captures how recent changes in trait values propagate through the direct-(causal-)effect network **G**. Therefore, the matrix $𝐆\in {\mathbb{R}}^{T\times T}$ is the causal network of our main interest, where each entry *g*_*ij*_ encodes the direct causal effect from *Y*_*j*_ to *Y*_*i*_. More specifically, if the value of *Y*_*j*_ changes by $\left(Y_{j}^{\left(t-1\right)}-Y_{j}^{\left(t-2\right)}\right)$ from time *t* − 2 to *t* − 1, then this change will cause *Y*_*i*_ to change at time *t* (comparing to time *t* − 1) by an amount of $g_{ij}\left(Y_{j}^{\left(t-1\right)}-Y_{j}^{\left(t-2\right)}\right)$. This second-order formulation reflects how changes in one trait can have (delayed) effects on other traits, a feature especially important for modeling feedback and cyclic relationships. The example in Fig 6 is an illustration of the recursive model.

Denote $G^{0}=I$, with **I** being the identity matrix. From Eqs (1) and (2), for any *t* ≥ 1 we have


$$\begin{array}{c} Y^{\left(t\right)}=\sum_{l=0}^{t-1}G^{l}\left(\Gamma Z+\epsilon \right). \end{array}$$ (3)

We make the following Assumption 1 about the causal network **G**.

**Assumption 1** We have (1) the spectral radius of **G** (i.e., the largest absolute value of all real and complex eigenvalues of **G**) is less than 1, and (2) all diagonal elements of **G** (i.e., *g*_*ii*_’s) are 0. In the Assumption 1, part (1) about the spectral radius (also commonly adopted in previous studies such as [54]) guarantees the dynamic model will converge to an equilibrium state, and part (2) requires that any trait cannot have direct effect on itself (i.e., there is no self-loop). With part (1) of Assumption 1, the inverse ${\left(I-G\right)}^{-1}$ always exists, and we can calculate the sum in (3) as


$$\begin{array}{c} Y^{\left(t\right)}={\left(I-G\right)}^{-1}\left(I-G^{t}\right)\left(\Gamma Z+\epsilon \right). \end{array}$$ (4)

#### Marginal model.

In most real-world applications, it is (almost) impossible to measure ***Y***^(*t*)^ at different times *t*’s, and thus the observed values of trai*t*s are treated as fixed. With part (1) of Assumption 1, we have **G**^*t*^ converges to a matrix of 0’s as t→∞. Therefore, we have $Y^{\left(t\right)}\to Y^{\left(\infty \right)}$ with the marginal model


$$\begin{array}{c} Y^{\left(\infty \right)}={\left(I-G\right)}^{-1}\left(\Gamma Z+\epsilon \right)=𝐁Z+\xi . \end{array}$$ (5)

In the marginal model (5), $𝐁={\left(I-G\right)}^{-1}\Gamma$ is a *T* × *S* matrix with its element $\beta_{ik}$ in the *i*^*th*^ row and *k*^*th*^ column being the *total (or marginal) effect* from *Z*_*k*_ to *Y*_*i*_. And $\xi ={\left(I-G\right)}^{-1}\epsilon$ is the vector of marginal random errors. For the convenience of notation, we denote $𝐖={\left(I-G\right)}^{-1}$, and *w*_*ij*_ is the element in the *i*^*th*^ row and *j*^*th*^ column of **W**.

Notably, Eq (5) coincides with the commonly-used linear structural equation model (SEM):


$$\begin{array}{c} Y^{\left(\infty \right)}=GY^{\left(\infty \right)}+\Gamma Z+\epsilon , \end{array}$$ (6)

which describes the relationship among endogenous variables (i.e., traits ***Y***) and exogenous variables (i.e. IVs ***Z***). While this SEM representation is static, our recursive modeling provides a dynamic interpretation of how changes in one trait propagate through the causal network over time. In particular, it reveals how the system evolves toward a steady state (or equilibrium) described in Eq (6).

#### MR-(causal-)effect network.

Suppose we have an ordered sequence of *M* variables $Q_{1},\cdots ,Q_{M}$ with an integer *M* ≥ 2, such that *Q*_*l*_ has non-zero direct effect on *Q*_*l*+1_ for l≤M−1. Since an IV can have direct effects on traits and cannot be directly affected by a trait or another IV, we have *Q*_1_ being an IV or a trait (i.e., $Q_{1}\in Z\cup Y$) and *Q*_*l*_ being a trait (i.e., $Q_{l}\in Y$) for *l* ≥ 2. Therefore, $Q_{1}\to \cdots \to Q_{M}$ is a *directed path* with *M* variables and of length (*M* − 1), and we denote this directed path as $Q_{M}=(Q_{1},\cdots ,Q_{M})$. Note that, different *Q*_*l*_’s can be the same trait due to existence of cycles in the causal network. With the concept of directed path, we can define valid IV as follows.

**Definition 1.** *For an IV* *Z*_*k*_ *and two traits* *Y*_*i*_ *and* *Y*_*j*_*, if for any directed path* ***Q***_*M*_ *from* *Z*_*k*_ *to* *Y*_*i*_ *(i.e.,* *Q*_1_ = *Z*_*k*_ *and* *Q*_*M*_ = *Y*_*i*_*), we always have* *Y*_*j*_ *is on the directed path (i.e., there is some 1 < l < M such that* *Q*_*l*_ = *Y*_*j*_*), then* *Z*_*k*_ *is a valid IV for* *Y*_*j*_ *to* *Y*_*i*_*. Furthermore, if* *Z*_*k*_ *is a valid IV for* *Y*_*j*_ *to* *Y*_*i*_ *for all* *i* ≠ *j* *(which is equivalent to* $\gamma_{jk}\neq 0$ *and* $\gamma_{ik}=0$ *for all* i≠j*), then* *Z*_*k*_ *is a valid IV for* *Y*_*j*_*.*

Definition 1 can be viewed as an extension of the original definition of valid IV in the univariable MR framework [1], in which a valid IV cannot affect the outcome through a path that is not mediated through the exposure. Note that, IV validity from Definition 1 is not conditional on the modeled network. If we add/exclude some variables to/from the network containing *Y*_*j*_ and *Y*_*i*_, the pleiotropic effects from *Z*_*k*_ to *Y*_*i*_ through directed paths ***Q***’s including these variables can be separated from/merged into the effects from *Z*_*k*_ to *Y*_*i*_ through other existing directed paths, and the total pleiotropic effect does not change. As shown by Theorem 1, we can define the MR effect of one trait on another with valid IVs.

**Theorem 1.** *Suppose* *Z*_*k*_ *is a valid IV for* *Y*_*j*_ *to* *Y*_*i*_*, we have the ratio of the two total (marginal) effects as* $\beta_{ik}/\beta_{jk}=w_{ij}/w_{jj}:=\theta_{ij}$*. Therefore,* $\theta_{ij}$ *is independent of* *Z*_*k*_ *and completely determined by* **W** *and thus by* **G***. We call* $\theta_{ij}$ *as the MR effect from* *Y*_*j*_ *to* *Y*_*i*_*.*

The proof of Theorem 1 is in the Section 1.1 in S1 Appendix. To get some intuition, consider a simple case that *Z*_*k*_ is a valid IV for *Y*_*j*_, i.e., $\gamma_{jk}\neq 0$ and $\gamma_{ik}=0$ for all i≠j. Recall that, from Eq (5), the total effects of SNPs on traits **B** are jointly determined by the direct-(causal-)effect network **G** and direct effects of SNPs on traits Γ as 𝐁=WΓ with $W=(I-G{)}^{-1}$, we have $\beta_{jk}=w_{jj}\gamma_{jk}$ and $\beta_{ik}=w_{ij}\gamma_{jk}$, therefore the ratio $\beta_{ik}/\beta_{jk}=w_{ij}/w_{jj}=\theta_{ij}$ is independent of *Z*_*k*_. Denote Θ as the *T* × *T* matrix representing the *MR-(causal-)effect network* with its element in the *i*^*th*^ row and *j*^*th*^ column as $\theta_{ij}$, from Theorem 1 we have the following key connection between Θ and **W** as


$$\begin{array}{c} \Theta =𝐖{\left(\text{Diag}(w)\right)}^{-1}, \end{array}$$ (7)

where $w=\left(w_{11},\cdots ,w_{TT}\right)$ and Diag(w) is the *T* × *T* diagonal matrix with diagonal elements as ***w***.

### MR2G: estimating causal networks from GWAS summary statistics

#### Estimating the MR-effect network via MRcML.

Model (5) describes marginal associations between SNPs and traits, therefore we can obtain estimates of elements in **B** from GWAS summary data. Denote estimate of $\beta_{ik}$ as ${\hat{\beta }}_{ik}$ for 1 ≤ *i* ≤ *T* and 1 ≤ *k* ≤ *S*, and standard error of ${\hat{\beta }}_{ik}$ as $\text{SE}({\hat{\beta }}_{ik})$. For any two traits *Y*_*j*_ and *Y*_*i*_, Theorem 1 allows us to combine multiple valid IVs to estimate the MR effect $\theta_{ij}$. However, a critical issue in MR is that SNPs being used as IVs may be invalid due to the widespread horizontal pleiotropy, leading to biased results. A number of MR methods have been developed to deal with invalid IVs [2–5], and one robust and powerful method is the constrained maximum likelihood-based MR method (MRcML) [6]. Under some mild conditions, including the plurality assumption for model identifiability and the ratio of sample sizes of the two traits being bounded, MRcML can consistently select invalid IVs to achieve unbiased inference of the causal effect. More details about MRcML can be found in [6]. For an ordered pair of traits (*Y*_*j*_, *Y*_*i*_) with 1 ≤ *i* ≠ *j* ≤ *T*, we apply MRcML to get an estimate ${\hat{\theta }}_{ij}$ of MR effect $\theta_{ij}$ with a set of independent SNPs as candidate IVs. The strategy of IV selection and screening is described in the following Section. With all ${\hat{\theta }}_{ij}$’s, we can get the estimated MR-effect network denoted as $\hat{\Theta }$.

#### IV screening procedure.

In the conventional MR analysis, the directions of causal effects are assumed from given exposures to outcomes. Therefore, SNPs being significantly associated with an exposure (typically with *p*-values less than 5 × 10^−8^) will be selected as candidate IVs for the exposure. However, when the directions of causal effects between two traits are unknown, as discussed in [12], selecting IVs based on *p*-values can lead to biased results. For example, suppose trait *Y*_1_ has a non-zero causal effect θ on trait *Y*_2_ but not the other way around, and *Z* is a valid IV for *Y*_1_ with effect γ. Therefore, *Z* has non-zero effect γ·θ on *Y*_2_ that is mediated through *Y*_1_. When the sample size of GWAS for *Y*_2_ is large enough, *Z* is significantly associated with *Y*_2_ and will be used as an IV for *Y*_2_ as well, falsely estimating the causal effect from *Y*_2_ to *Y*_1_ as 1/θ.

A simple IV screening rule based on Steiger’s method was proposed in [12], which can effectively alleviate this problem when estimating bi-directional causal relationships between two traits. The same screening rule was also adopted in the Graph-MRcML method [23] when estimating total causal effects between traits. Specifically, for bi-directional MR between *Y*_*i*_ and *Y*_*j*_, if a SNP is significantly associated with both traits, it is only used as an IV for the trait with which it has a stronger correlation. This approach has demonstrated good performance in examining bi-directional causal relationships by alleviating violation of MR assumptions required by many robust MR methods (e.g., the InSIDE assumption, plurality assumption), particularly in scenarios involving bi-directional causality [12]. We refer to this screening approach as **ScreenPair**.

While ScreenPair is effective in bi-directional MR analysis, it does not fully account for the complex causal network structure. For instance, consider the causal graph among 3 traits shown in Fig 2A, SNPs in the set ***Z***_3_ are marginally associated with all three traits. Depending on the effect sizes of $Y_{3}\to Y_{1}$ and $Y_{3}\to Y_{2}$ (and consequently, the correlations of SNPs in ***Z***_3_ with *Y*_1_ and *Y*_2_), the ScreenPair approach may select these SNPs as IVs for *Y*_1_ when performing MR of $Y_{1}\to Y_{2}$, provided their correlations with *Y*_1_ are stronger, and vice versa. However, all SNPs in ***Z***_3_ are invalid IVs for investigating the bi-directional relationship between *Y*_1_ and *Y*_2_ due to (correlated) horizontal pleiotropy. The following Assumption 2 is a generalization of the assumption in [12] to the causal network model.

**Assumption 2** For an IV *Z*_*k*_ and a trait *Y*_*j*_, if $\left|\text{cor}(Z_{k},Y_{j})|>|\text{cor}(Z_{k},Y_{i})\right|$ for all i≠j, then $\gamma_{jk}\neq 0$.

Assumption 2 states that, if *Z*_*k*_ has the strongest correlation with *Y*_*j*_, then it has a non-zero direct effect on *Y*_*j*_. This assumption is reasonable in practice given that causal effects between complex traits are typically small. Note that, it does not exclude direct effects of *Z*_*k*_ on other traits. With Assumption 2, we can construct a set of candidate IVs for *Y*_*j*_ as ${ℳ}_{j}=\{Z_{k}|1\leq k\leq S,\text{ and }|\text{cor}(Z_{k},Y_{j})|>|\text{cor}(Z_{k},Y_{i})|\text{ for all }i\neq j\}$. If a SNP is significantly associated with multiple traits, it will only be used as an IV for the trait with the strongest correlation. We refer to this approach as **ScreenMax**. Under ScreenMax, SNPs in $Z_{1},Z_{2},Z_{3}$ will only be used as IVs for *Y*_1_, *Y*_2_ and *Y*_3_ respectively, thereby avoiding the issue caused by ScreenPair in Fig 2A.

However, ScreenMax may be overly restrictive as it may exclude some valid IVs. For example, in Fig 2B, SNPs in ***Z***_3_ are indeed valid IVs for *Y*_1_ when performing MR of $Y_{1}\to Y_{2}$. Accordingly, we can augment the IV set selected by ScreenMax by adding back some potentially valid IVs selected by ScreenPair. The procedure is summarized as follows:

1. For each pair of traits *Y*_*j*_ and *Y*_*i*_, we first identify a set of independent SNPs, with each SNP being significantly associated with either/both of the two traits. Then we use **ScreenPair** to construct IV sets ${𝒫}_{ij}$ for performing MR $Y_{j}\to Y_{i}$, and ${𝒫}_{ji}$ for performing MR $Y_{i}\to Y_{j}$.
2. Next we use **ScreenMax** to refine these sets. Specifically, for ${𝒫}_{ij}$, we retain only the IVs that exhibit the strongest correlation with *Y*_*j*_ among all other traits with which they are significantly associated, denoting this set as ${ℳ}_{ij}$. Similarly, for ${𝒫}_{ji}$, we retain only the IVs that have the strongest correlation with *Y*_*i*_, denoting this set as ${ℳ}_{ji}$. We then construct an estimated direct-effect graph ${\hat{G}}_{ℳ}$ using IVs selected by ScreenMax.
3. Based on the estimated ${\hat{G}}_{ℳ}$ (with Bonferroni adjustment for multiple testing), we augment IV sets as follows: for each SNP $Z_{k}\in {𝒫}_{ij}⧵{ℳ}_{ij}$, consider each trait *Y*_*l*_ that is significantly associated with *Z*_*k*_ and has a stronger correlation with *Z*_*k*_ than *Y*_*j*_. If removing *Y*_*j*_ from ${\hat{G}}_{ℳ}$, results in *Y*_*l*_ having no path to *Y*_*i*_, then *Z*_*k*_ is added back to the IV set ${ℳ}_{ij}\cup \{Z_{k}\}$. A similar procedure is also applied to ${𝒫}_{ji}⧵{ℳ}_{ji}$.

We refer to the above procedure as **ScreenAug**. This procedure balances the strengths of both methods: it reduces bias and false discoveries by excluding potentially invalid IVs and enhances power by reintroducing potentially valid IVs excluded due to overly stringent criteria.

#### Algorithm to estimate the direct-(causal-)effect network from a MR-(causal-)effect network.

Based on the key connection (7), we have $\left(I-G{)}^{-1}=W=\Theta \text{Diag}(w\right)$, which suggests that $(I-G{)}^{-1}$ is a scaled version of Θ with column scaling matrix Diag(w). Therefore, we have


$$\begin{array}{c} 𝐆=I-{\left(\text{Diag}(w)\right)}^{-1}\Theta^{-1}. \end{array}$$ (8)

To ensure zero diagonal elements in **G** as required by Assumption 1 (i.e., no self-loop), which is equivalent to having the diagonal elements of ${\left(\text{Diag}(w)\right)}^{-1}\Theta^{-1}$ being 1, we propose the following algorithm to estimate *w*_*jj*_ and **G** given the estimated $\hat{\Theta }$.

**Algorithm 1. Estimation of G given $\hat{\Theta }$**

**Input:** $\hat{\Theta }$

${\hat{w}}_{jj}\leftarrow ({\hat{\Theta }}^{-1}{)}_{jj}$

$\hat{W}\leftarrow \hat{\Theta }\text{Diag}(\hat{w})$

$\hat{G}\leftarrow I-{\hat{W}}^{-1}$

**return** $\hat{G}$

The following Theorem 2 provides theoretical guarantees for inference on the causal network **G**, and the conditions it requires and the proof can be found in Section 1.2 in S1 Appendix.

**Theorem 2.** *Under suitable regularity conditions,* $\hat{G}$ *is a consistent estimator of* **G** *and follows an asymptotic normal distribution.*

However, since the asymptotic covariance of $\hat{G}$ is analytically complex and does not account for uncertainty arising from model selection in MRcML under finite samples, we adopt a data perturbation strategy (i.e., a parametric bootstrap [55]) for inference, similar to that in our previous work [23], as detailed below.

Specifically, we jointly perturb the entire matrix of GWAS effect estimates $\hat{B}$ across all traits to account for both inter-GWAS correlations and LD structure among SNPs. Letting $Z=\hat{B}⊘S$ denote the matrix of GWAS Z-scores, with ⊘ denoting the element-wise matrix division, where **S** is the matrix of standard errors. We generate perturbed datasets $Z^{\left(b\right)}$ from a matrix normal distribution MN(Z,R,P), with **R** and **P** representing the SNP LD matrix and trait correlation matrix (due to sample overlap), respectively. The perturbed GWAS effect estimates are then reconstructed as ${\hat{B}}^{\left(b\right)}=Z^{\left(b\right)}⊙S$, with ⊙ denoting the element-wise matrix multiplication. In our application, LD matrices are computed within 1703 approximately independent LD blocks [56] using 1000 Genomes Phase 3 [57] European population via TwoSampleMR [58]. For each perturbed dataset, we apply bidirectional MRcML to get estimated ${\hat{\Theta }}^{\left(b\right)}$ and then apply Algorithm 1 to get estimated ${\hat{G}}^{\left(b\right)}$. Repeating this procedure *B* times, we compute the element-wise mean and standard deviation of $\{{\hat{G}}^{\left(b\right)}{\}}_{b=1}^{B}$ to obtain point estimates $\hat{G}$ and standard errors, and calculate *p*-values using a standard normal distribution.

### Relationship with other methods

#### Relationship with network deconvolution.

[24] proposed a network deconvolution formula


$$\begin{array}{c} G_{tot}=G+G^{2}+\ldots \end{array}$$ (9)

$$\begin{array}{c} =(I-G{)}^{-1}G, \end{array}$$ (10)

where the second equality holds under the assumption that the spectral radius of **G** is less than 1. The definition of a total-(causal-)effect graph **G**_*tot*_ in Eq (9) can be motivated by repeatedly substituting the right-hand side of Eq (6) for ***Y***^(∞)^ in the right-hand side [59]:


$$\begin{array}{cc} Y^{\left(\infty \right)} & =G(GY^{\left(\infty \right)}+\Gamma Z+\epsilon )+\Gamma Z+\epsilon \\ & =G^{2}Y^{\left(\infty \right)}+(I+G)(\Gamma Z+\epsilon )\\ & =G^{3}Y^{\left(\infty \right)}+(I+G+G^{2})(\Gamma Z+\epsilon )\\ & \cdots \\ & =G^{k}Y^{\left(\infty \right)}+(I+G+G^{2}+\ldots +G^{k-1})(\Gamma Z+\epsilon ), \end{array}$$

where the matrix **G**^*k*^ represents the indirect effects of length *k* for traits ***Y*** on each other. For example, if we denote $g_{ij}^{\left(k\right)}$ be the element in **G**^*k*^, then $g_{ij}^{\left(2\right)}=\sum_{l}g_{il}g_{lj}$ represents the indirect effect of length 2 from *Y*_*j*_ to *Y*_*i*_ through one node *Y*_*l*_. Similarly $g_{ij}^{\left(3\right)}=\sum_{l}g_{il}g_{lj}^{\left(2\right)}=\sum_{l}g_{il}\sum_{v}g_{lv}g_{vj}=\sum_{l,v}g_{il}g_{lv}g_{vj}$ represents the indirect effect of length 3 from *Y*_*j*_ to *Y*_*i*_ through two nodes *Y*_*l*_ and *Y*_*v*_, and so on. Then the total effect can be defined by the sum $G+G^{2}+\ldots +G^{k}$, and *k* can get arbitrarily large due to possible cycles among ***Y***.

The above infinite summation Eq (9) converges to $(I-G{)}^{-1}G$ *if and only if* the spectral radius of **G** is less than 1, otherwise $G_{tot}$ may not be well defined. In particular, under the spectral radius assumption, we have $W=(I-G{)}^{-1}=I+G+G^{2}+\ldots =I+G_{tot}$, which gives us an insightful relationship between the total-effect network and the MR-effect network


$$\begin{array}{c} G_{tot}=W-I=\Theta \text{Diag}(w)-I=\Theta \left(\text{Diag}(t)+I\right)-I, \end{array}$$ (11)

where $t=(t_{11},\ldots ,t_{TT})$ and *t*_*jj*_ is the *j*^*th*^ diagonal element of $G_{tot}$. In other words, MR estimates a *partial* total effect without accounting for cyclic effects if cycles are present. Cyclic effects manifest as self-loops in the total-effect graph, i.e., *t*_*jj*_. One immediate result is that, if there are no cycles in the direct-effect graph, there will be no self-loops in the total-effect graph (i.e., *t*_*jj*_ = 0), making $G_{tot}=\Theta -I$.

#### Relationship with multivariable MR.

MVMR [7] estimates the direct effect of each exposure on an outcome, conditional on the other exposures. A natural question is whether the direct effects in **G** coincide with the corresponding MVMR estimands. In S1 Appendix Section 3.2 Proposition 1, we prove that, when all other traits are included as MVMR exposures for outcome *Y*_*i*_, the MVMR estimand equals $g_{i,-i}$, the *i*-th row of **G**.

### Simulation setups

#### Simulation with three traits.

In this section, we considered two simple scenarios in Fig 2A and Fig 2B to illustrate the idea of IV screening. We generated individual-level data based on the marginal model (5),


$$\begin{array}{c} 𝐘=(I-G{)}^{-1}\Gamma 𝐙+(I-G{)}^{-1}\epsilon , \end{array}$$ (12)

where **Y** was a 3 × *N* matrix representing three traits, **Z** was a 30 × *N* matrix of 30 SNPs, each independently generated from a binomial distribution with a minor allele frequency of 0.3. Γ was a 3 × 30 matrix, in which the first 8 SNPs had non-zero effects $\gamma_{1k}$ only on *Y*_1_, the next 7 SNPs had non-zero effects $\gamma_{2k}$ only on *Y*_2_, and the remaining 15 SNPs had non-zero effects $\gamma_{3k}$ only on *Y*_3_, and the non-zero effect sizes ($\gamma_{ik}$) were drawn from a uniform distribution over (−0.2,−0.1)∪(0.1,0.2). In other words, $|Z_{1}|=8,|Z_{2}|=7,|Z_{3}|=15$. The random errors ϵ were drawn from $MVN(0,\Sigma_{e})$, where $\Sigma_{e}$ had a first-order autoregressive structure with correlation 0.4 to reflect the unmeasured confounder effect. For Fig 2A, $G=\left(\begin{array}{ccc} 0 & 0 & 0.2\\ 0 & 0 & 0.1\\ 0 & 0 & 0 \end{array}\right)$, and for Fig 2B, $G=\left(\begin{array}{ccc} 0 & 0 & 0.5\\ 0.05 & 0 & 0\\ 0 & 0.5 & 0 \end{array}\right)$. Under this setup, the average proportion of phenotypic variance explained by the genetic component across replicates was approximately 7.1%, 6.0%, and 12.8% for the three traits in the acyclic setting (Fig 2A), and 6.3%, 6.0%, and 8.9% in the cyclic setting (Fig 2B).

We considered sample sizes of *N* = 50 000 and *N* = 200 000. After generating individual-level data, we regressed rows of **Y** on **Z** to obtain the GWAS estimates for the three traits. We then applied MR2G to infer the direct-effect graph using IVs selected by the three different IV selection approaches, **ScreenPair**, **ScreenMax** and **ScreenAug**, with the *p*-value threshold of 5e−8. Additionally, we also compared the results of Graph-MRcML, which uses IVs selected via ScreenPair and the network deconvolution algorithm proposed in [23].

For each scenario, we conducted 200 simulation replicates. We used the *p*-value threshold of 0.05 to evaluate type-I error and a Bonferroni-corrected threshold of 0.05/6 to evaluate power.

#### Simulation based on real GWAS summary data.

To evaluate the performance of the proposed method, we designed simulation studies closely following the structure of our real data application. We focused on a subset of six traits, including three risk factors (BMI, LDL and SBP) and three cardiovascular diseases (CAD, AF and stroke) in the simulation.

We first extracted the 1056 SNPs across 392 LD blocks that were used in the real data analysis for these 6 traits, ensuring that the simulated data retains a realistic LD pattern. We directly generated GWAS summary statistics for the 1056 SNPs as follows.

1. We first selected SNPs that were genome-wide significant in the real GWAS datasets to have non-zero direct effect $\gamma_{jk}$ on the corresponding trait *Y*_*j*_, and the effect sizes $\gamma_{jk}$ were drawn from a normal distribution $𝒩(0,0.1^{2})$;
2. The GWAS effect size matrix **B** was then generated using the relationship $B=(I-G{)}^{-1}\Gamma$. **G** was based on the real data application on the 6 traits (Fig 2C).
3. Finally, the observed GWAS estimates were generated as $\hat{B}=B+S⊙E$, where **E** follows a matrix normal distribution E~MN(0,R,P). Here, **S** represents the standard errors of the GWAS estimates, directly extracted from the real data. **R** is the (block-diagonal) LD-matrix of SNPs, derived from the 1000 Genomes Phase 3 [57] European population. **P** is the correlation matrix among the 6 GWAS data, which is an identity matrix in this case.

With the simulated GWAS summary statistics, we applied the same preprocessing step as in the real data analysis to obtain a set of approximately LD-independent SNPs for each pair of traits. We then applied MR2G (with *B* = 100 data perturbations) using IVs selected by the three different IV selection approaches, ScreenPair, ScreenMax, and ScreenAug, to estimate direct-effect network among traits. We conducted 200 simulation replicates. We used the *p*-value threshold of 0.05 to evaluate type-I error and a Bonferroni-corrected threshold of 0.05/30 to evaluate power.

### Real data application

We applied MR2G to infer the direct-effect network among 9 cardiometabolic risk factors and 6 diseases. The 9 risk factors were triglycerides (TG), low-density lipoprotein (LDL) cholesterol, high-density lipoprotein (HDL) cholesterol, height, body-mass index (BMI), systolic blood pressure (SBP), fasting glucose (FG), smoke (cigarette per day), and alcohol (alcoholic drinks per week). The 6 diseases were coronary artery disease (CAD), stroke, type 2 diabetes (T2D), asthma (more as a negative control), atrial fibrillation (AF), and Alzheimer’s disease (AD). The sample sizes for the 15 GWAS datasets ranged from 10 083–1 030 836, with a median of 256 879. All GWAS summary statistics are publicly available.

We pre-processed the GWAS summary statistics following the procedure described in [12] and [23]. Specifically, for each pair of traits, we used the TwoSampleMR package [60] to obtain a set of approximately LD-independent SNPs that were significantly associated with at least one of the traits (using a *p*-value cutoff of 5e-8). We then aggregated these SNPs across all trait pairs and used ieugwasr::ld_matrix function from the ieugwasr package [61], with the 1000 Genomes Phase 3 [57] European population as the reference panel, to extract the LD matrix **R** among all selected SNPs. The correlation matrix **P** among the 15 GWAS data was estimated by bivariate LD score regression [62], and for the pairs with small correlations (|ρ|<0.1), we set them to be 0 as before [23].

## Supporting information

S1 Appendix Supplementary file with proofs, details of methods, additional real data analysis results and additional simulation results. (PDF)
