# Supplementary material for: MR2G: A novel framework for causal network inference using GWAS summary data
Source: PLoS Genet. 2026 May 26;22(5):e1012144. doi: 10.1371/journal.pgen.1012144 (PMC13210398; doi:10.1371/journal.pgen.1012144)
Supplement: S1 Appendix — (PDF) [file pgen.1012144.s001.pdf]

Supporting Information for "MR2G: A novel framework for causal network inference using GWAS summary data"

Zhaotong Lin[1], Wei Pan[2], and Haoran Xue[3*]

[1]Department of Statistics, Florida State University, Tallahassee, Florida, United States of America

[2]Division of Biostatistics and Health Data Science, School of Public Health, University of Minnesota, Minneapolis, Minnesota, United States of America

[3]Department of Biostatistics, City University of Hong Kong, Hong Kong, China

[*]Correspondence: xue.hr@cityu.edu.hk

Contents

1 Supplementary figures **3**

2 Supplementary tables **18**

3 Supplementary methods **19**

3.1 Theoretical results 19

3.1.1 Proof of Theorem 1 19

3.1.2 Asymptotic property of the estimated causal network 21

3.2 Connection between MVMR and MR2G direct effects 22

3.2.1 Setup and notation 22

3.2.2 MVMR estimand 23

3.2.3 Equivalence when all traits are included as exposures 24
3.3 Additional analyses for the simulation with three traits 26
3.3.1 Setup in the main text 26
3.3.2 Varying AR(1) residual correlation 27
3.3.3 Varying genetic effects and network edge strengths 27
3.3.4 Explicit latent confounder 28
3.3.5 Comparison with conventional UVMR methods 30
3.4 Additional analyses for the simulation with real GWAS data 31
3.4.1 Scaling the direct-effect graph 31
3.5 Sensitivity of ScreenAug to the reference graph used for IV augmentation 32
3.6 Computational efficiency 35

1 Supplementary figures

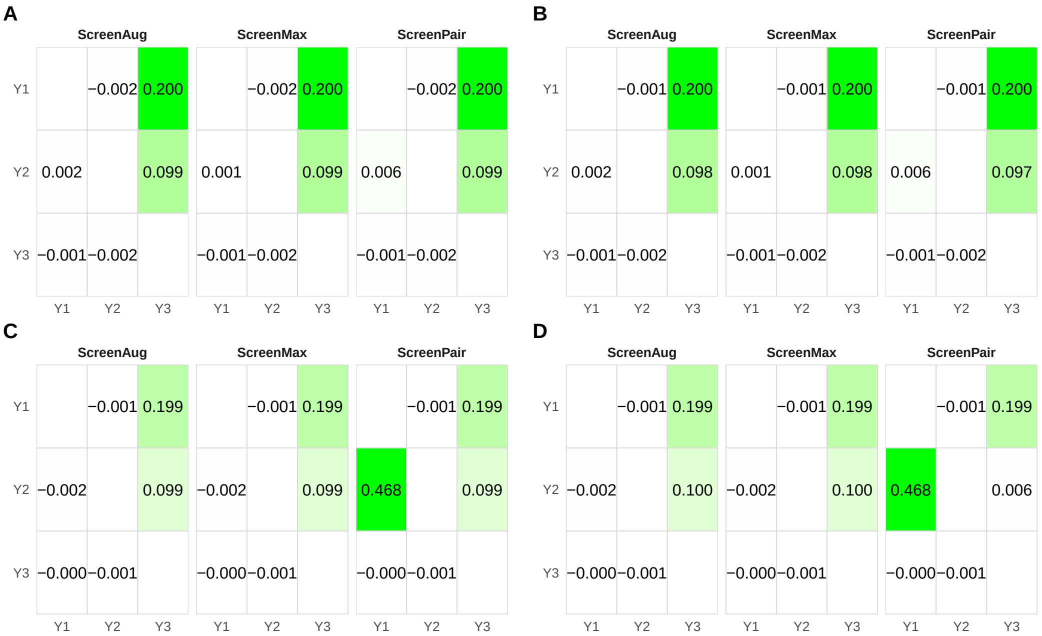

Figure A. **Average estimated MR-effect graph (A, C) and direct-effect graph (B, D) in the simulation with three acyclic traits in Figure 2A.** Panels **A** and **B** show results for sample size $N = 50{,}000$, while Panels **C** and **D** show results for $N = 200{,}000$.

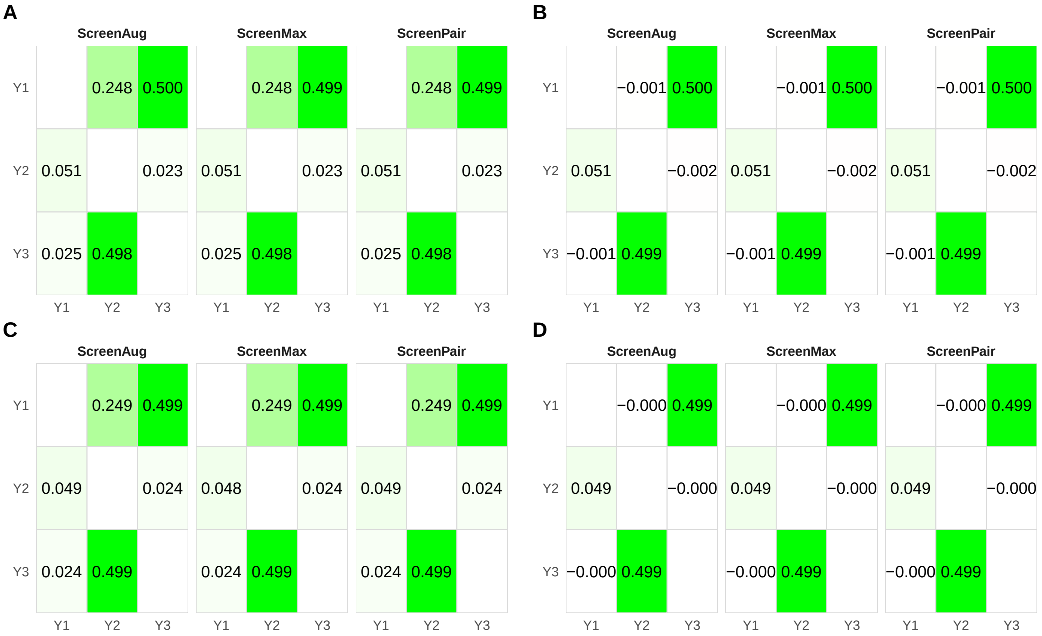

Figure B. **Average estimated MR-effect graph (A, C) and direct-effect graph (B, D) in the simulation with three acyclic traits in Figure 2B.** Panels **A** and **B** show results for sample size $N = 50{,}000$, while Panels **C** and **D** show results for $N = 200{,}000$.

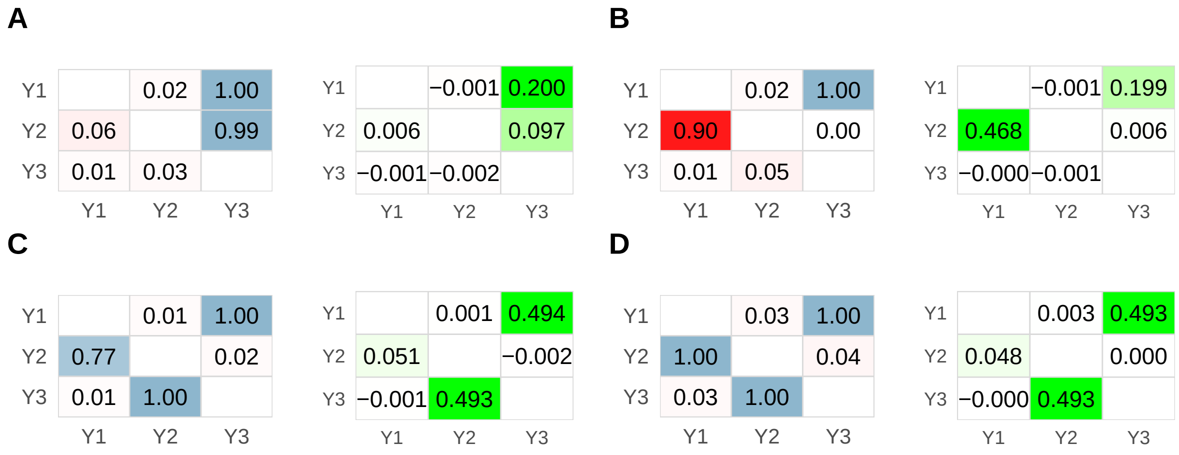

Figure C. **Results of Graph-MRcML in simulation with three traits.** Panels **A** and **B** show results in acyclic setting Figure 2A for sample size $N = 50{,}000$ and $N = 200{,}000$ respectively; Panels **C** and **D** show results in cyclic setting Figure 2B for sample size $N = 50{,}000$ and $N = 200{,}000$ respectively. In each panel, the left heatmap is the rejection rate, and the right heatmap is the average estimated direct-effect graph.

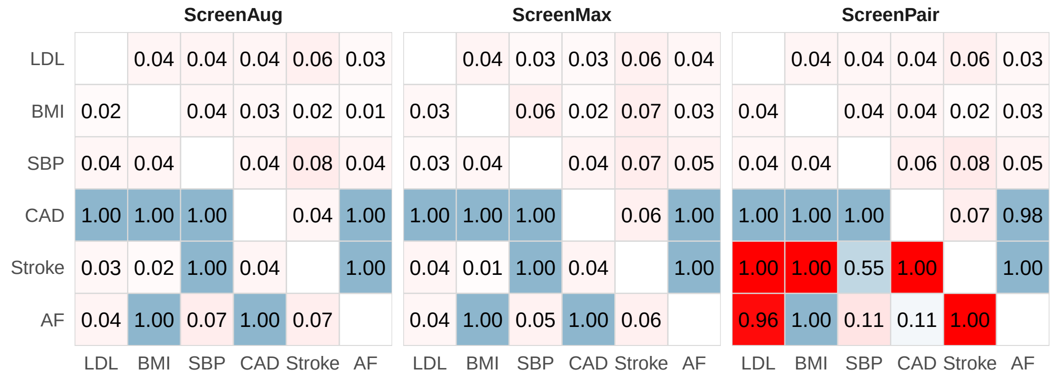

Figure D. **Rejection rate of MR2G with three IV screening strategies in the simulation scenario Figure 2C.** In the heatmaps, power is shown using a blue scale and type-I error is shown using a red scale.

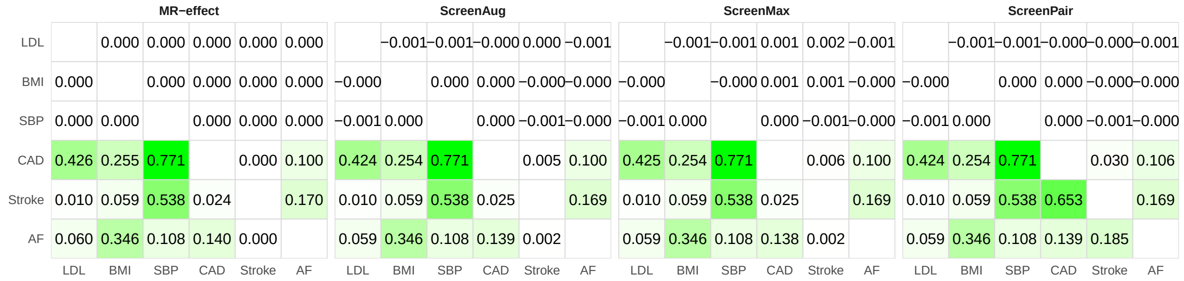

Figure E. **MR effect estimates for the simulation scenario Figure 2C.** From left to right are the true MR-effect matrix $\boldsymbol{\Theta}$, and the average $\widehat{\boldsymbol{\Theta}}$ estimated by MR2G coupled with ScreenAug, ScreenMax and ScreenPair.

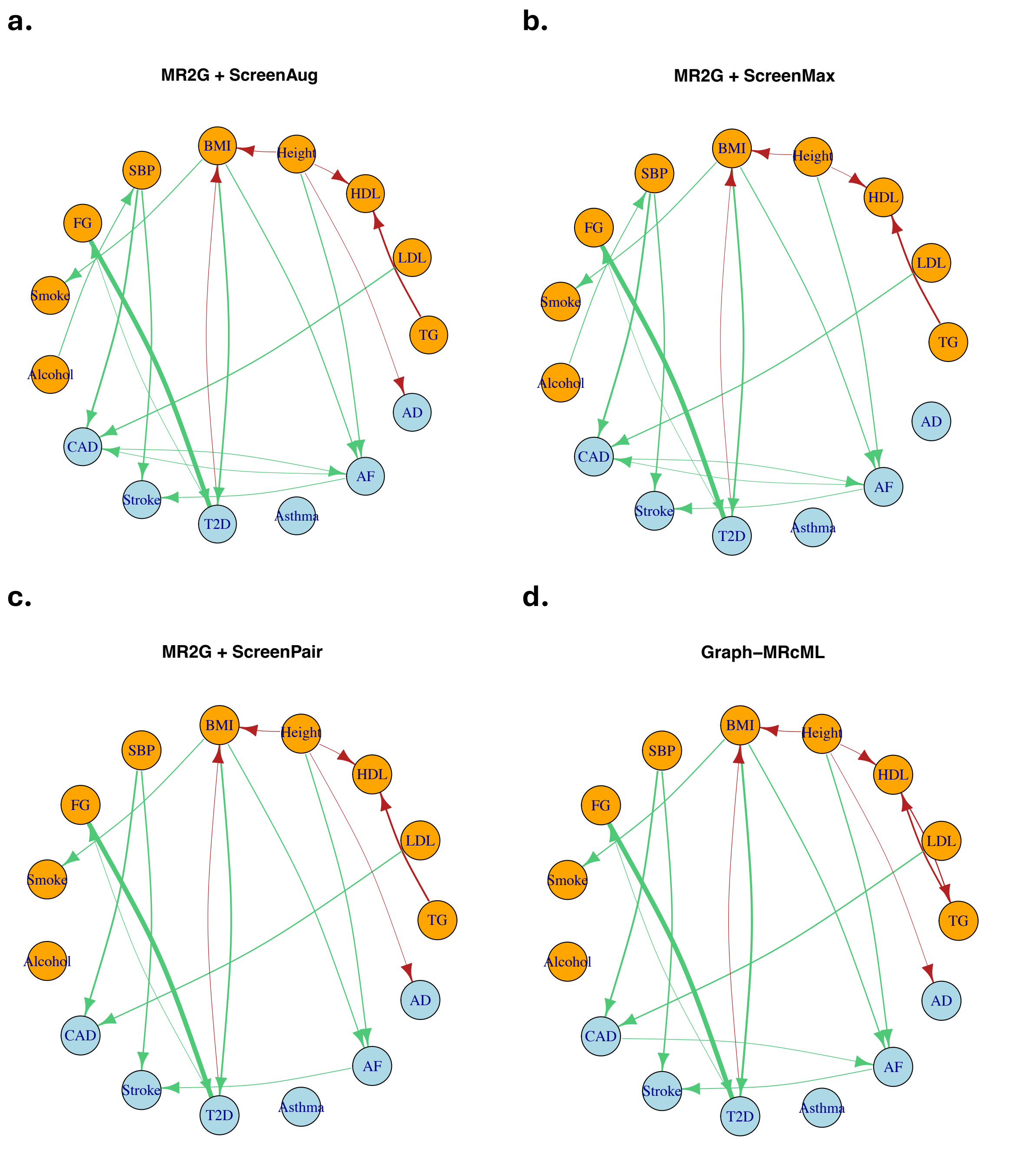

Figure F. Estimated direct-effect graph among 15 traits by MR2G coupled with (a) ScreenAug, (b) ScreenMax, (c) ScreenPair, and (d) Graph-MRcML [1].

	TG	LDL	HDL	Height	BMI	SBP	FG	Smoke	Alcohol	CAD	Stroke	T2D	Asthma	AF	AD
AD	−0.00 (0.02)	0.00 (0.01)	−0.01 (0.01)	−0.02*** (0.01)	0.02 (0.01)	−0.00 (0.02)	0.04 (0.03)	0.02 (0.01)	0.01 (0.03)	0.01 (0.01)	0.01 (0.02)	−0.00 (0.01)	−0.01 (0.01)	−0.00 (0.00)	
AF	0.07 (0.07)	−0.04 (0.03)	0.08 (0.05)	0.37*** (0.03)	0.34*** (0.05)	0.24 (0.12)	0.06 (0.14)	0.03 (0.06)	0.16 (0.15)	0.13*** (0.03)	0.07 (0.12)	−0.03 (0.03)	0.03 (0.03)		−0.21 (0.15)
Asthma	−0.13 (0.10)	0.02 (0.05)	−0.08 (0.08)	0.02 (0.04)	0.12 (0.08)	0.27 (0.13)	−0.21 (0.18)	0.05 (0.06)	−0.15 (0.22)	0.01 (0.07)	−0.15 (0.12)	0.01 (0.05)		0.02 (0.03)	−0.27 (0.27)
T2D	−0.07 (0.20)	−0.05 (0.09)	−0.13 (0.16)	−0.02 (0.08)	0.64*** (0.16)	0.57 (0.22)	2.06*** (0.33)	0.00 (0.19)	−0.18 (0.42)	−0.03 (0.10)	−0.03 (0.21)		−0.03 (0.10)	0.04 (0.06)	−0.52 (0.34)
Stroke	−0.12 (0.06)	0.06 (0.04)	−0.06 (0.05)	−0.03 (0.03)	−0.03 (0.06)	0.50*** (0.09)	−0.17 (0.14)	0.05 (0.06)	0.03 (0.11)	0.12 (0.04)		0.07 (0.03)	−0.02 (0.02)	0.17*** (0.02)	0.15 (0.15)
CAD	−0.02 (0.15)	0.42*** (0.07)	−0.23 (0.13)	−0.08 (0.03)	0.11 (0.07)	0.73*** (0.10)	0.21 (0.11)	0.05 (0.08)	−0.01 (0.19)		0.04 (0.10)	0.03 (0.03)	0.03 (0.03)	0.10*** (0.03)	−0.29 (0.21)
Alcohol	−0.05 (0.02)	0.00 (0.01)	−0.01 (0.01)	−0.01 (0.01)	−0.03 (0.04)	0.02 (0.02)	0.01 (0.04)	0.01 (0.02)		0.00 (0.01)	−0.00 (0.01)	−0.02 (0.01)	−0.00 (0.01)	−0.01 (0.00)	0.01 (0.04)
Smoke	0.07 (0.05)	−0.01 (0.02)	0.01 (0.04)	0.05 (0.02)	0.30*** (0.05)	0.09 (0.05)	0.12 (0.09)		−0.09 (0.10)	−0.02 (0.02)	0.04 (0.05)	−0.02 (0.02)	−0.01 (0.02)	−0.00 (0.01)	−0.17 (0.15)
FG	0.02 (0.03)	−0.02 (0.01)	−0.02 (0.02)	0.01 (0.01)	0.01 (0.03)	−0.05 (0.04)		0.01 (0.02)	0.08 (0.07)	0.01 (0.01)	0.03 (0.03)	0.07*** (0.01)	0.02 (0.01)	−0.01 (0.01)	0.03 (0.05)
SBP	0.05 (0.03)	−0.02 (0.02)	0.03 (0.02)	−0.03 (0.02)	0.03 (0.04)		0.00 (0.07)	−0.00 (0.03)	0.28*** (0.07)	0.08 (0.03)	0.07 (0.07)	0.03 (0.02)	0.01 (0.01)	−0.03 (0.01)	0.07 (0.11)
BMI	−0.05 (0.04)	−0.03 (0.01)	−0.06 (0.03)	−0.10*** (0.02)		−0.05 (0.05)	0.14 (0.08)	−0.02 (0.04)	0.19 (0.13)	−0.03 (0.02)	−0.01 (0.04)	−0.07*** (0.02)	−0.00 (0.01)	0.00 (0.01)	−0.23 (0.09)
Height	−0.20 (0.08)	0.03 (0.04)	−0.07 (0.06)		−0.00 (0.05)	−0.05 (0.07)	0.03 (0.11)	0.00 (0.02)	−0.25 (0.28)	0.04 (0.02)	−0.03 (0.06)	0.01 (0.02)	−0.01 (0.01)	−0.00 (0.02)	−0.05 (0.16)
HDL	−0.67*** (0.13)	−0.01 (0.06)		−0.07*** (0.02)	−0.08 (0.04)	0.04 (0.07)	0.12 (0.13)	−0.03 (0.03)	0.20 (0.13)	−0.01 (0.02)	0.06 (0.05)	0.00 (0.03)	0.02 (0.02)	−0.02 (0.01)	−0.13 (0.24)
LDL	0.49 (0.20)		0.15 (0.13)	−0.05 (0.02)	−0.08 (0.04)	−0.06 (0.06)	0.04 (0.08)	0.05 (0.03)	−0.05 (0.15)	−0.03 (0.02)	−0.06 (0.06)	−0.01 (0.02)	−0.02 (0.02)	−0.00 (0.01)	−0.18 (0.18)
TG		0.08 (0.06)	−0.37 (0.12)	−0.03 (0.02)	0.08 (0.04)	0.06 (0.06)	−0.04 (0.09)	0.03 (0.03)	0.07 (0.13)	0.00 (0.02)	0.07 (0.04)	0.05 (0.03)	0.02 (0.02)	−0.01 (0.01)	0.05 (0.23)

Figure G. **Estimated direct-effect graph among 15 traits by MR2G-ScreenAug.** The number in each cell corresponds to estimate (standard error) of the direct effect from the column trait to the row trait. '***' denotes significant edges at Bonferroni adjusted level of 0.05/181, where 181 is the number of independent tests; '*' denotes edges with $p < 0.001$.

	TG	LDL	HDL	Height	BMI	SBP	FG	Smoke	Alcohol	CAD	Stroke	T2D	Asthma	AF	AD
AD	0.01 (0.01)	0.00 (0.01)	−0.01 (0.01)	**−0.03*** (0.01)**	0.03 (0.01)	0.01 (0.01)	0.04 (0.02)	0.02 (0.01)	0.03 (0.02)	0.01 (0.01)	0.01 (0.02)	−0.00 (0.01)	−0.01 (0.01)	0.00 (0.00)	
AF	−0.01 (0.03)	0.02 (0.02)	0.01 (0.03)	**0.32*** (0.02)**	**0.38*** (0.04)**	**0.32*** (0.09)**	0.07 (0.10)	0.04 (0.05)	0.25 (0.10)	**0.16*** (0.02)**	0.08 (0.11)	−0.03 (0.03)	0.03 (0.03)		−0.32 (0.13)
Asthma	−0.06 (0.07)	−0.00 (0.04)	−0.02 (0.05)	0.02 (0.03)	0.13 (0.07)	0.16 (0.09)	−0.18 (0.14)	0.03 (0.06)	−0.12 (0.21)	−0.00 (0.06)	−0.14 (0.11)	−0.01 (0.04)		−0.01 (0.03)	−0.31 (0.26)
T2D	0.05 (0.12)	−0.13 (0.07)	−0.20 (0.10)	−0.05 (0.06)	**0.87*** (0.13)**	0.49* (0.15)	**2.05*** (0.32)**	0.02 (0.18)	0.26 (0.38)	0.01 (0.09)	0.06 (0.18)		0.01 (0.10)	0.02 (0.04)	−0.59 (0.31)
Stroke	0.00 (0.03)	0.11* (0.03)	−0.05 (0.03)	−0.02 (0.02)	0.15 (0.05)	**0.67*** (0.07)**	0.07 (0.10)	0.07 (0.05)	0.27 (0.09)	**0.19*** (0.04)**		0.08 (0.02)	−0.02 (0.02)	**0.17*** (0.01)**	0.04 (0.13)
CAD	**0.33*** (0.06)**	**0.43*** (0.07)**	−0.27 (0.08)	**−0.10*** (0.02)**	**0.31*** (0.05)**	**0.78*** (0.07)**	**0.31*** (0.07)**	0.10 (0.08)	0.26 (0.16)		0.09 (0.07)	0.07 (0.02)	0.03 (0.03)	**0.09*** (0.02)**	−0.37 (0.17)
Alcohol	−0.04 (0.01)	0.00 (0.01)	0.01 (0.01)	−0.01 (0.00)	−0.05 (0.04)	0.01 (0.01)	−0.02 (0.03)	0.01 (0.01)		0.00 (0.01)	−0.00 (0.01)	−0.02 (0.01)	−0.00 (0.01)	−0.01 (0.00)	0.03 (0.04)
Smoke	0.05 (0.03)	−0.02 (0.02)	−0.02 (0.02)	0.02 (0.01)	**0.31*** (0.05)**	0.06 (0.03)	0.08 (0.07)		−0.02 (0.09)	−0.02 (0.02)	0.04 (0.04)	−0.02 (0.01)	−0.00 (0.01)	−0.00 (0.01)	−0.20 (0.14)
FG	0.03 (0.02)	−0.02 (0.01)	−0.05 (0.01)	0.01 (0.01)	**0.09*** (0.02)**	0.02 (0.02)		0.02 (0.02)	0.09 (0.07)	0.01 (0.01)	0.03 (0.03)	**0.07*** (0.01)**	0.01 (0.01)	0.00 (0.01)	−0.01 (0.04)
SBP	0.03 (0.02)	0.01 (0.01)	−0.01 (0.01)	**−0.06*** (0.01)**	0.07 (0.03)		0.09 (0.04)	0.02 (0.03)	**0.34*** (0.07)**	0.08 (0.03)	0.08 (0.07)	0.04 (0.02)	0.01 (0.01)	−0.02 (0.01)	0.04 (0.10)
BMI	−0.03 (0.03)	**−0.04*** (0.01)**	−0.01 (0.01)	**−0.08*** (0.01)**		−0.11 (0.03)	−0.03 (0.06)	−0.02 (0.04)	0.17 (0.12)	−0.04 (0.01)	−0.02 (0.04)	**−0.07*** (0.01)**	−0.00 (0.01)	−0.00 (0.01)	−0.15 (0.08)
Height	−0.12 (0.05)	0.02 (0.03)	−0.00 (0.03)		−0.00 (0.04)	−0.07 (0.05)	0.06 (0.08)	−0.01 (0.02)	−0.29 (0.28)	0.02 (0.01)	−0.05 (0.05)	0.01 (0.01)	−0.01 (0.01)	−0.00 (0.01)	−0.08 (0.16)
HDL	**−0.67*** (0.12)**	−0.09 (0.03)		−0.05* (0.01)	**−0.24*** (0.03)**	0.01 (0.04)	0.09 (0.09)	−0.06 (0.02)	0.20 (0.10)	−0.00 (0.02)	0.02 (0.04)	−0.02 (0.02)	0.01 (0.01)	−0.02 (0.01)	−0.12 (0.20)
LDL	0.39 (0.13)		−0.04 (0.05)	**−0.05*** (0.01)**	−0.02 (0.03)	−0.05 (0.04)	0.03 (0.04)	0.06 (0.03)	−0.04 (0.12)	−0.04 (0.02)	−0.03 (0.05)	0.01 (0.01)	−0.01 (0.02)	−0.02 (0.01)	−0.15 (0.12)
TG		0.12 (0.06)	−0.39* (0.12)	−0.03 (0.01)	**0.23*** (0.02)**	0.12 (0.04)	0.03 (0.03)	0.06 (0.03)	0.06 (0.13)	0.02 (0.02)	0.07 (0.03)	0.05 (0.03)	0.01 (0.02)	0.01 (0.01)	0.04 (0.22)

Figure H. **Estimated MR-effect graph among 15 traits by MRcML-ScreenAug.** The number in each cell corresponds to estimate (standard error) of the MR effect from the column trait to the row trait. '***' denotes significant edges after Bonferroni adjustment; '*' denotes edges with $p < 0.001$.

IVW

	TG	LDL	HDL	Height	BMI	SBP	FG	Smoke	Alcohol	CAD	Stroke	T2D	Asthma	AF	AD
AD	0.04 (0.03)	**0.05*** (0.01)**	−0.04 (0.04)	**−0.03*** (0.00)**	0.03 (0.01)	0.01 (0.01)	0.02 (0.03)	0.03 (0.01)	0.03 (0.03)	0.04 (0.03)	0.01 (0.02)	−0.00 (0.01)	−0.01 (0.01)	0.00 (0.00)	
AF	−0.00 (0.04)	0.03 (0.02)	−0.00 (0.03)	**0.29*** (0.03)**	**0.35*** (0.04)**	**0.29*** (0.08)**	0.06 (0.09)	0.02 (0.04)	0.13 (0.12)	**0.15*** (0.02)**	0.64 (0.58)	−0.01 (0.03)	0.06 (0.03)		−0.20 (0.14)
Asthma	−0.10 (0.06)	0.01 (0.05)	−0.02 (0.07)	0.01 (0.04)	0.12 (0.07)	0.13 (0.08)	−0.09 (0.21)	0.03 (0.06)	−0.11 (0.21)	−0.04 (0.06)	−0.19 (0.10)	−0.00 (0.04)		0.01 (0.04)	−0.39 (0.28)
T2D	0.05 (0.10)	−0.10 (0.06)	−0.23 (0.09)	−0.04 (0.05)	0.73* (0.22)	**0.51*** (0.12)**	1.47 (0.71)	−0.02 (0.09)	0.22 (0.31)	0.02 (0.07)	0.17 (0.14)		0.00 (0.06)	0.02 (0.04)	−0.60 (0.22)
Stroke	0.01 (0.03)	0.10 (0.03)	−0.08 (0.03)	−0.03 (0.02)	0.15 (0.06)	**0.65*** (0.07)**	0.18 (0.13)	0.07 (0.05)	0.21 (0.11)	**0.21*** (0.03)**		**0.07*** (0.02)**	−0.01 (0.02)	**0.19*** (0.02)**	0.06 (0.10)
CAD	**0.34*** (0.05)**	**0.44*** (0.06)**	**−0.23*** (0.06)**	**−0.09*** (0.02)**	**0.29*** (0.06)**	**0.60*** (0.08)**	0.28 (0.10)	0.05 (0.04)	0.20 (0.15)		0.32 (0.11)	0.07* (0.02)	0.02 (0.04)	**0.10*** (0.02)**	0.29 (0.11)
Alcohol	**−0.06*** (0.01)**	−0.00 (0.01)	0.02 (0.01)	−0.01 (0.01)	−0.07 (0.03)	0.03 (0.03)	0.02 (0.06)	0.00 (0.01)		0.01 (0.01)	−0.00 (0.01)	−0.02 (0.01)	−0.00 (0.01)	−0.01 (0.00)	0.03 (0.03)
Smoke	0.05 (0.03)	−0.01 (0.02)	−0.02 (0.03)	0.03 (0.02)	**0.22*** (0.04)**	0.06 (0.04)	0.06 (0.07)		0.00 (0.11)	−0.01 (0.02)	0.01 (0.04)	−0.02 (0.01)	−0.00 (0.01)	0.00 (0.01)	−0.18 (0.11)
FG	−0.02 (0.02)	−0.02 (0.02)	−0.04 (0.02)	0.01 (0.01)	0.07 (0.02)	0.03 (0.02)		0.02 (0.02)	0.17 (0.09)	0.01 (0.01)	0.04 (0.02)	0.10* (0.03)	0.01 (0.01)	−0.00 (0.01)	−0.01 (0.04)
SBP	0.04 (0.02)	0.01 (0.01)	−0.02 (0.02)	**−0.07*** (0.01)**	0.04 (0.03)		0.07 (0.04)	0.01 (0.02)	0.27* (0.08)	**0.07*** (0.02)**	0.15 (0.06)	0.03 (0.01)	0.01 (0.01)	−0.01 (0.01)	0.04 (0.07)
BMI	−0.04 (0.02)	−0.04 (0.01)	−0.03 (0.03)	**−0.07*** (0.01)**		−0.09 (0.03)	−0.06 (0.08)	−0.03 (0.02)	−0.11 (0.19)	−0.03 (0.01)	−0.03 (0.03)	−0.02 (0.05)	−0.00 (0.01)	−0.01 (0.01)	−0.16 (0.05)
Height	−0.08 (0.03)	−0.03 (0.03)	0.01 (0.03)		−0.01 (0.05)	−0.11 (0.07)	0.09 (0.07)	−0.01 (0.02)	0.11 (0.21)	0.00 (0.02)	−0.17 (0.17)	0.00 (0.01)	−0.03 (0.03)	0.00 (0.02)	0.00 (0.05)
HDL	**−0.48*** (0.11)**	−0.09 (0.06)		−0.04 (0.01)	**−0.23*** (0.05)**	−0.04 (0.05)	0.11 (0.07)	−0.06 (0.02)	0.44* (0.13)	−0.12 (0.04)	−0.07 (0.05)	−0.03 (0.02)	−0.03 (0.02)	−0.01 (0.01)	**−0.29*** (0.04)**
LDL	0.26 (0.11)		−0.09 (0.07)	**−0.06*** (0.01)**	−0.03 (0.06)	−0.07 (0.04)	0.06 (0.16)	0.05 (0.03)	−0.09 (0.15)	0.27 (0.09)	−0.06 (0.05)	0.01 (0.01)	−0.02 (0.02)	−0.01 (0.01)	1.86 (0.73)
TG		0.13 (0.06)	**−0.42*** (0.07)**	−0.05 (0.02)	**0.23*** (0.03)**	0.09 (0.03)	−0.27 (0.34)	0.06 (0.03)	−0.77 (0.39)	0.07 (0.04)	0.07 (0.03)	0.04 (0.02)	0.01 (0.02)	0.01 (0.01)	**0.52*** (0.12)**

Figure I. **Estimated MR-effect graph among 15 traits by MR-IVW.** The number in each cell corresponds to estimate (standard error) of the MR effect from the column trait to the row trait. '***' denotes significant edges after Bonferroni adjustment; '*' denotes edges with $p < 0.001$.

Egger

	TG	LDL	HDL	Height	BMI	SBP	FG	Smoke	Alcohol	CAD	Stroke	T2D	Asthma	AF	AD
AD	0.06 (0.05)	**0.08*** (0.02)**	−0.04 (0.07)	−0.03 (0.01)	−0.00 (0.03)	0.01 (0.04)	0.04 (0.07)	0.01 (0.02)	0.01 (0.04)	0.03 (0.07)	0.04 (0.10)	−0.01 (0.02)	−0.06 (0.03)	0.00 (0.01)	
AF	0.02 (0.07)	0.01 (0.03)	0.01 (0.06)	**0.28*** (0.07)**	0.29 (0.10)	0.35 (0.26)	0.07 (0.21)	−0.05 (0.07)	0.07 (0.21)	0.14 (0.05)	0.53 (4.35)	−0.08 (0.08)	0.14 (0.11)		−0.98 (0.42)
Asthma	−0.01 (0.10)	−0.03 (0.08)	−0.02 (0.14)	0.04 (0.10)	0.29 (0.16)	0.03 (0.29)	−0.35 (0.51)	0.07 (0.09)	−0.68 (0.75)	−0.32 (0.16)	0.38 (0.65)	−0.11 (0.12)		−0.10 (0.08)	−1.05 (1.10)
T2D	−0.17 (0.17)	−0.20 (0.11)	0.04 (0.15)	0.17 (0.14)	1.22 (0.54)	−0.03 (0.43)	−1.77 (1.36)	−0.22 (0.14)	1.91 (0.90)	−0.14 (0.15)	0.15 (0.91)		−0.04 (0.20)	0.07 (0.08)	−0.97 (0.35)
Stroke	−0.05 (0.05)	0.13 (0.05)	0.09 (0.06)	−0.02 (0.06)	0.10 (0.14)	**0.89*** (0.23)**	−0.42 (0.23)	−0.00 (0.09)	0.26 (0.15)	0.12 (0.07)		0.08 (0.05)	0.01 (0.07)	**0.15*** (0.03)**	0.45 (0.21)
CAD	0.20 (0.08)	**0.46*** (0.08)**	−0.04 (0.11)	0.02 (0.06)	0.03 (0.14)	0.78 (0.27)	0.39 (0.25)	−0.06 (0.07)	0.41 (0.24)		0.31 (0.74)	−0.00 (0.05)	−0.07 (0.15)	0.03 (0.04)	**0.62*** (0.11)**
Alcohol	−0.06 (0.02)	0.02 (0.01)	0.01 (0.02)	0.01 (0.02)	−0.12 (0.06)	0.13 (0.08)	−0.06 (0.16)	−0.01 (0.02)		0.02 (0.01)	−0.05 (0.06)	0.02 (0.01)	−0.01 (0.02)	0.00 (0.01)	0.01 (0.08)
Smoke	0.02 (0.05)	−0.02 (0.02)	0.01 (0.05)	0.09 (0.04)	−0.02 (0.11)	0.04 (0.11)	−0.09 (0.16)		−0.06 (0.18)	−0.04 (0.04)	−0.02 (0.27)	−0.02 (0.03)	−0.01 (0.05)	0.01 (0.02)	−0.42 (0.18)
FG	−0.04 (0.04)	−0.02 (0.03)	−0.03 (0.03)	−0.01 (0.02)	0.05 (0.06)	0.02 (0.08)		0.01 (0.03)	0.53 (0.23)	0.04 (0.02)	−0.28 (0.13)	0.02 (0.08)	0.04 (0.04)	−0.01 (0.01)	0.00 (0.06)
SBP	−0.03 (0.03)	0.02 (0.01)	0.03 (0.03)	−0.04 (0.03)	0.05 (0.09)		0.24 (0.08)	−0.03 (0.03)	0.31 (0.13)	−0.04 (0.04)	−0.07 (0.33)	0.03 (0.03)	−0.04 (0.03)	−0.05 (0.02)	0.13 (0.12)
BMI	−0.02 (0.04)	−0.04 (0.02)	−0.02 (0.05)	−0.05 (0.04)		−0.34 (0.11)	0.18 (0.18)	−0.07 (0.03)	0.01 (0.53)	−0.04 (0.03)	−0.06 (0.27)	−0.17 (0.13)	−0.04 (0.03)	−0.00 (0.02)	−0.22 (0.07)
Height	−0.05 (0.05)	−0.02 (0.04)	−0.01 (0.06)		0.03 (0.13)	−0.38 (0.24)	−0.14 (0.18)	−0.00 (0.03)	−0.16 (0.56)	0.01 (0.04)	−1.87 (3.43)	0.03 (0.04)	0.14 (0.10)	0.03 (0.03)	0.07 (0.05)
HDL	−0.35 (0.18)	−0.15 (0.09)		−0.08 (0.04)	−0.27 (0.11)	0.01 (0.15)	−0.27 (0.09)	−0.04 (0.03)	0.70 (0.34)	0.04 (0.10)	−0.17 (0.41)	0.08 (0.05)	−0.09 (0.07)	0.01 (0.02)	**−0.37*** (0.04)**
LDL	0.07 (0.17)		−0.02 (0.13)	−0.01 (0.03)	0.00 (0.14)	−0.03 (0.12)	−0.22 (0.38)	0.06 (0.06)	−0.54 (0.44)	0.49 (0.21)	−0.33 (0.40)	0.01 (0.02)	−0.08 (0.05)	0.00 (0.02)	**7.01*** (0.98)**
TG		−0.04 (0.09)	−0.23 (0.12)	0.03 (0.05)	0.19 (0.06)	−0.05 (0.11)	1.09 (0.69)	0.03 (0.04)	−2.56 (1.09)	−0.09 (0.07)	0.18 (0.21)	−0.06 (0.03)	−0.02 (0.06)	0.02 (0.02)	**0.90*** (0.17)**

Figure J. **Estimated MR-effect graph among 15 traits by MR-Egger.** The number in each cell corresponds to estimate (standard error) of the MR effect from the column trait to the row trait. '***' denotes significant edges after Bonferroni adjustment; '*' denotes edges with $p < 0.001$.

PRESSO

	TG	LDL	HDL	Height	BMI	SBP	FG	Smoke	Alcohol	CAD	Stroke	T2D	Asthma	AF	AD
AD	0.01 (0.01)	0.01 (0.01)	−0.02 (0.01)	**−0.03*** (0.00)**	0.03 (0.01)	0.01 (0.01)	0.04 (0.02)	0.02 (0.01)	0.03 (0.03)	0.01 (0.01)	0.02 (0.01)	−0.00 (0.01)	−0.01 (0.01)	0.00 (0.00)	
AF	−0.00 (0.03)	0.03 (0.02)	0.01 (0.03)	**0.31*** (0.02)**	**0.35*** (0.04)**	**0.31*** (0.06)**	0.10 (0.07)	0.08 (0.06)	0.21 (0.09)	**0.15*** (0.02)**	0.56 (0.11)	−0.01 (0.02)	0.04 (0.02)		−0.27 (0.13)
Asthma	−0.06 (0.05)	−0.02 (0.04)	−0.01 (0.05)	0.03 (0.03)	0.12 (0.07)	0.14 (0.08)	−0.18 (0.15)	0.03 (0.05)	−0.11 (0.21)	−0.02 (0.04)	−0.06 (0.07)	−0.02 (0.04)		−0.01 (0.03)	−0.39 (0.28)
T2D	−0.00 (0.09)	−0.10 (0.06)	−0.24 (0.08)	−0.06 (0.05)	**0.90*** (0.11)**	**0.53*** (0.12)**	**1.81*** (0.27)**	−0.02 (0.09)	0.36 (0.29)	0.03 (0.06)	0.09 (0.13)		0.00 (0.06)	0.03 (0.03)	−0.60 (0.19)
Stroke	0.00 (0.03)	0.09 (0.03)	−0.06 (0.03)	−0.02 (0.02)	0.15 (0.05)	**0.62*** (0.06)**	0.28 (0.13)	0.08 (0.05)	0.24 (0.10)	**0.20*** (0.03)**		0.07 (0.02)	−0.01 (0.02)	**0.17*** (0.02)**	0.06 (0.10)
CAD	**0.33*** (0.04)**	**0.44*** (0.03)**	**−0.25*** (0.04)**	**−0.09*** (0.02)**	**0.28*** (0.05)**	**0.69*** (0.06)**	**0.34*** (0.05)**	0.15 (0.05)	0.25 (0.12)		0.24 (0.07)	0.07 (0.02)	0.04 (0.02)	**0.09*** (0.02)**	−0.13 (0.15)
Alcohol	**−0.04*** (0.01)**	−0.00 (0.01)	0.01 (0.01)	−0.01 (0.00)	−0.07* (0.02)	0.01 (0.01)	−0.01 (0.02)	0.01 (0.01)		0.00 (0.01)	−0.00 (0.01)	−0.02 (0.00)	−0.01 (0.01)	−0.01 (0.00)	0.03 (0.03)
Smoke	0.05 (0.03)	−0.01 (0.02)	−0.02 (0.02)	0.02 (0.01)	**0.29*** (0.04)**	0.04 (0.03)	0.06 (0.07)		−0.02 (0.08)	−0.02 (0.02)	0.01 (0.04)	−0.02 (0.01)	−0.00 (0.01)	−0.00 (0.01)	−0.10 (0.12)
FG	0.03 (0.02)	−0.02 (0.01)	−0.04* (0.01)	0.00 (0.01)	**0.08*** (0.02)**	0.03 (0.02)		0.02 (0.02)	0.06 (0.06)	0.01 (0.01)	0.04 (0.02)	0.08* (0.02)	0.01 (0.01)	−0.00 (0.01)	−0.01 (0.04)
SBP	0.03 (0.01)	0.01 (0.01)	−0.02 (0.01)	**−0.05*** (0.01)**	0.06 (0.02)		0.05 (0.04)	0.01 (0.01)	**0.30*** (0.04)**	**0.08*** (0.01)**	0.14 (0.04)	0.03 (0.01)	0.01 (0.01)	−0.01 (0.01)	−0.00 (0.07)
BMI	−0.04 (0.02)	**−0.04*** (0.01)**	−0.02 (0.02)	**−0.08*** (0.01)**		−0.09 (0.03)	−0.08 (0.04)	−0.03 (0.02)	0.03 (0.09)	−0.04 (0.01)	0.00 (0.02)	−0.05 (0.01)	−0.00 (0.01)	−0.00 (0.01)	−0.16 (0.05)
Height	−0.09* (0.02)	−0.01 (0.01)	−0.00 (0.02)		0.00 (0.03)	−0.05 (0.04)	0.04 (0.05)	−0.01 (0.02)	−0.15 (0.12)	0.02 (0.01)	−0.08 (0.04)	0.01 (0.01)	−0.01 (0.01)	−0.00 (0.01)	0.00 (0.05)
HDL	**−0.51*** (0.03)**	**−0.09*** (0.02)**		−0.04* (0.01)	**−0.24*** (0.03)**	0.01 (0.03)	0.07 (0.05)	−0.06 (0.02)	0.28 (0.08)	−0.04 (0.02)	−0.01 (0.03)	−0.03 (0.01)	0.00 (0.01)	−0.02 (0.01)	**−0.29*** (0.04)**
LDL	**0.28*** (0.03)**		−0.06 (0.03)	**−0.05*** (0.01)**	−0.02 (0.02)	−0.04 (0.03)	0.04 (0.05)	0.06 (0.03)	−0.06 (0.11)	**0.19*** (0.03)**	−0.06 (0.03)	0.01 (0.01)	−0.01 (0.01)	−0.01 (0.01)	1.46 (0.64)
TG		**0.14*** (0.02)**	**−0.43*** (0.03)**	−0.04* (0.01)	**0.22*** (0.02)**	0.11* (0.03)	−0.12 (0.06)	0.06 (0.03)	−0.26 (0.11)	0.04 (0.02)	0.05 (0.03)	0.05 (0.01)	0.02 (0.02)	0.01 (0.01)	0.07 (0.13)

Figure K. **Estimated MR-effect graph among 15 traits by MR-PRESSO.** The number in each cell corresponds to estimate (standard error) of the MR effect from the column trait to the row trait. '***' denotes significant edges after Bonferroni adjustment; '*' denotes edges with $p < 0.001$.

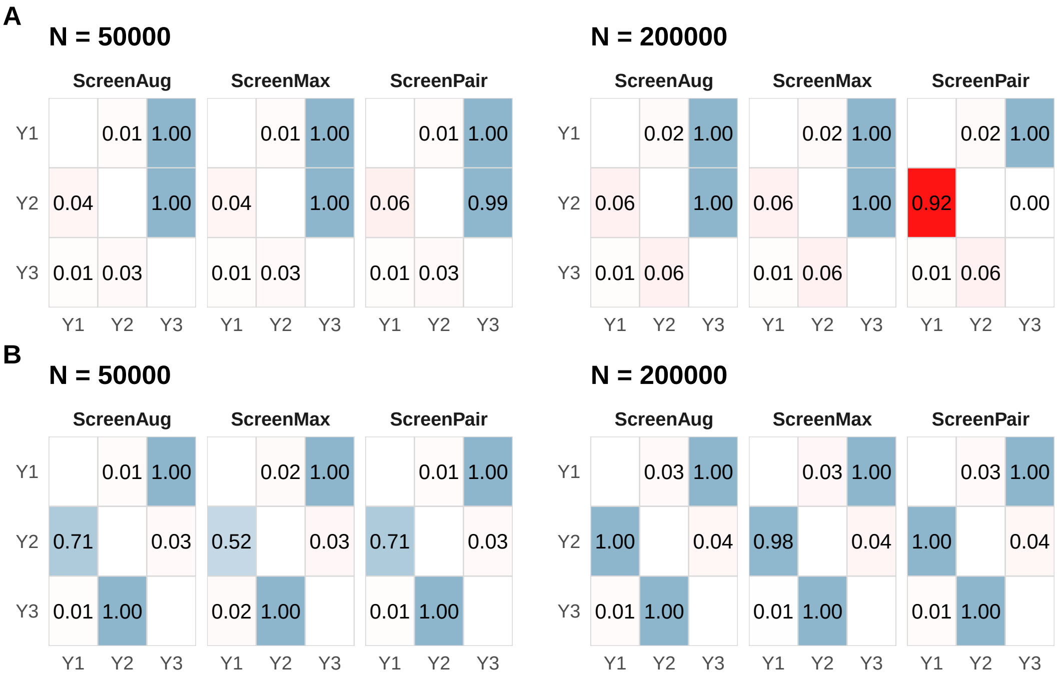

Figure L. **Proportion of rejections across 200 replicates under AR(1) residual correlation with** $\rho = 0.1$**.** Panel **A** corresponds to the acyclic setting in Fig. 2A, and Panel **B** corresponds to the cyclic setting in Fig. 2B. The left column shows results for N = 50,000, and the right column shows results for N = 200,000. In the heatmaps, power is shown using a blue scale and type-I error is shown using a red scale.

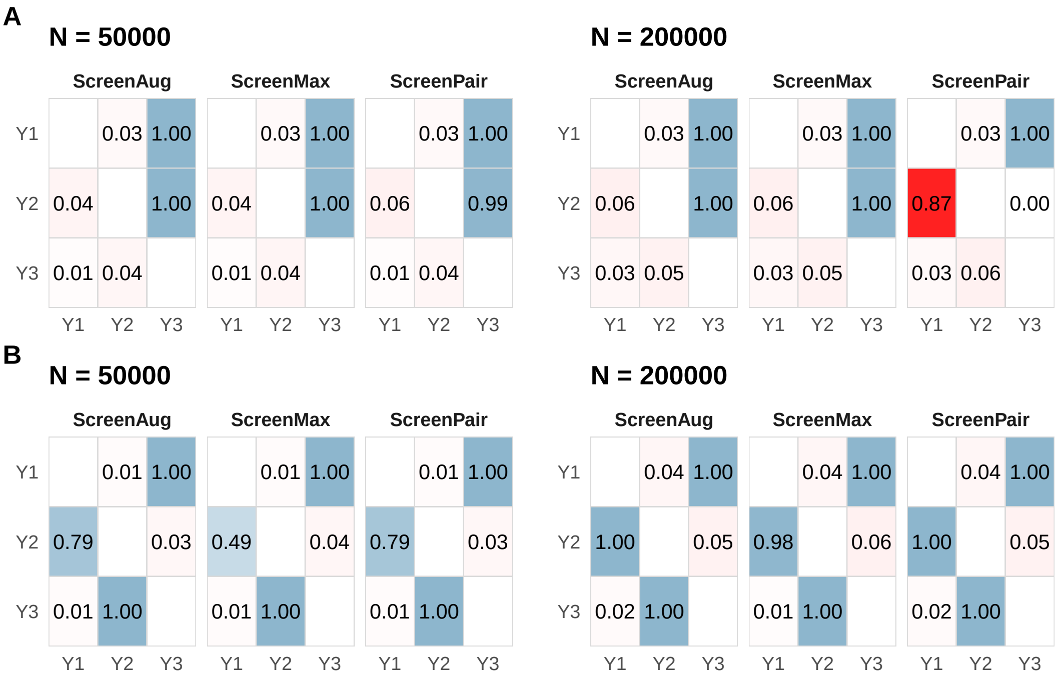

Figure M. **Proportion of rejections across 200 replicates under AR(1) residual correlation with** $\rho = 0.7$**.** Panel **A** corresponds to the acyclic setting in Fig. 2A, and Panel **B** corresponds to the cyclic setting in Fig. 2B. The left column shows results for $N = 50{,}000$, and the right column shows results for $N = 200{,}000$.

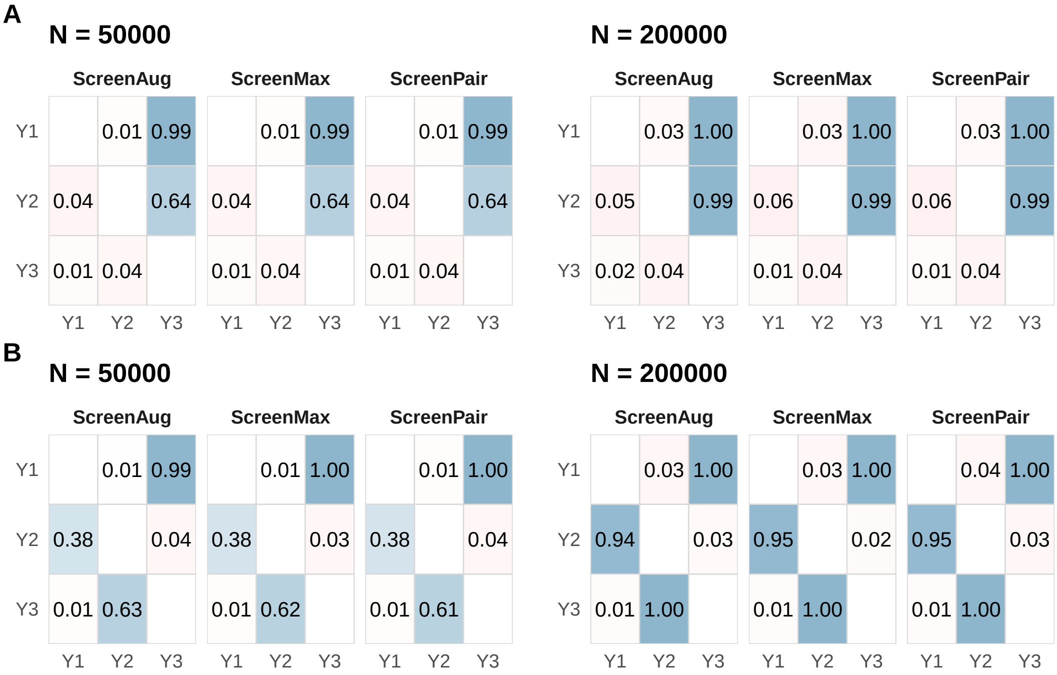

Figure N. **Proportion of rejections across 200 replicates under weaker causal effects and SNP heritability of 0.05.** Panel **A** corresponds to the acyclic setting in Fig. 2A, and Panel **B** corresponds to the cyclic setting in Fig. 2B. The left column shows results for $N = 50{,}000$, and the right column shows results for $N = 200{,}000$.

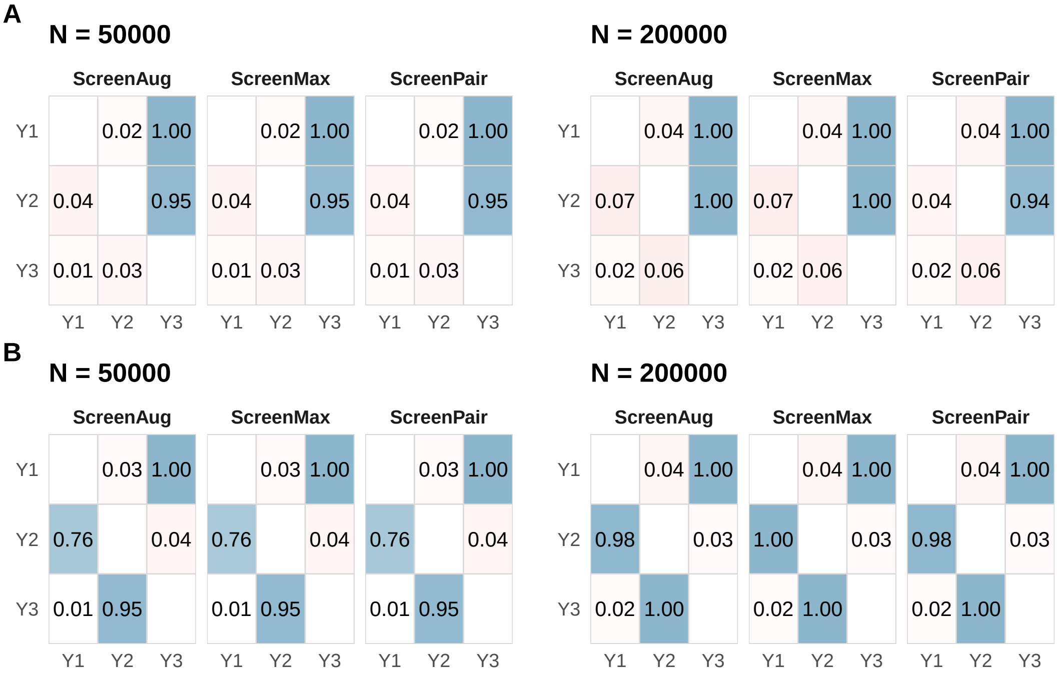

Figure O. **Proportion of rejections across 200 replicates under weaker causal effects and SNP heritability of 0.1.** Panel **A** corresponds to the acyclic setting in Fig. 2A, and Panel **B** corresponds to the cyclic setting in Fig. 2B. The left column shows results for $N = 50{,}000$, and the right column shows results for $N = 200{,}000$.

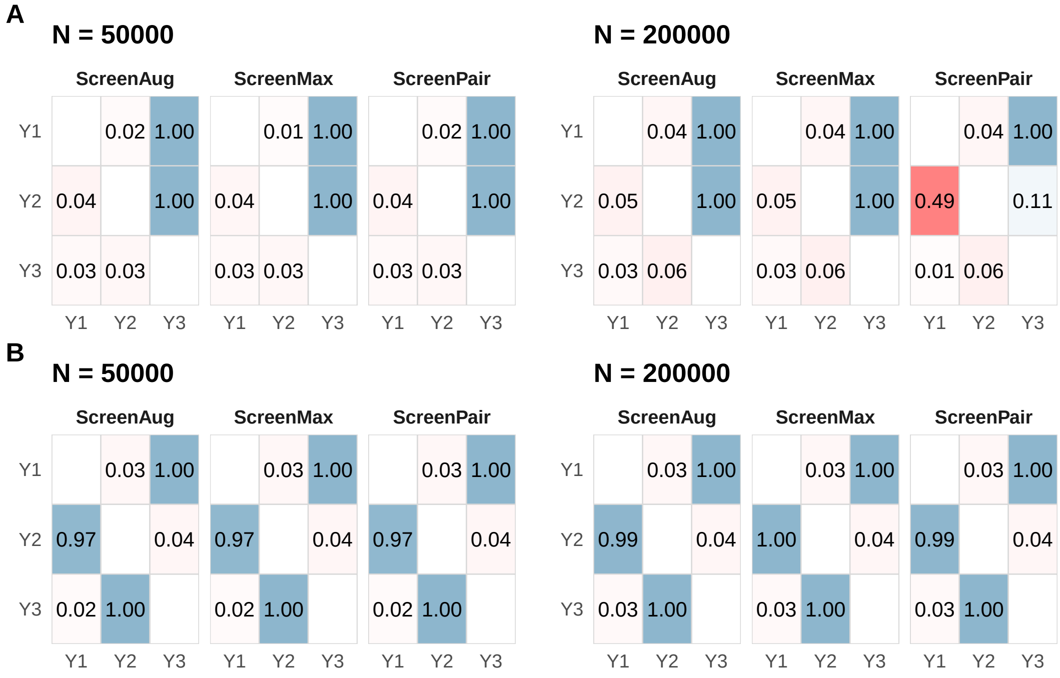

Figure P. **Proportion of rejections across 200 replicates under weaker causal effects and SNP heritability of 0.2.** Panel **A** corresponds to the acyclic setting in Fig. 2A, and Panel **B** corresponds to the cyclic setting in Fig. 2B. The left column shows results for $N = 50{,}000$, and the right column shows results for $N = 200{,}000$.

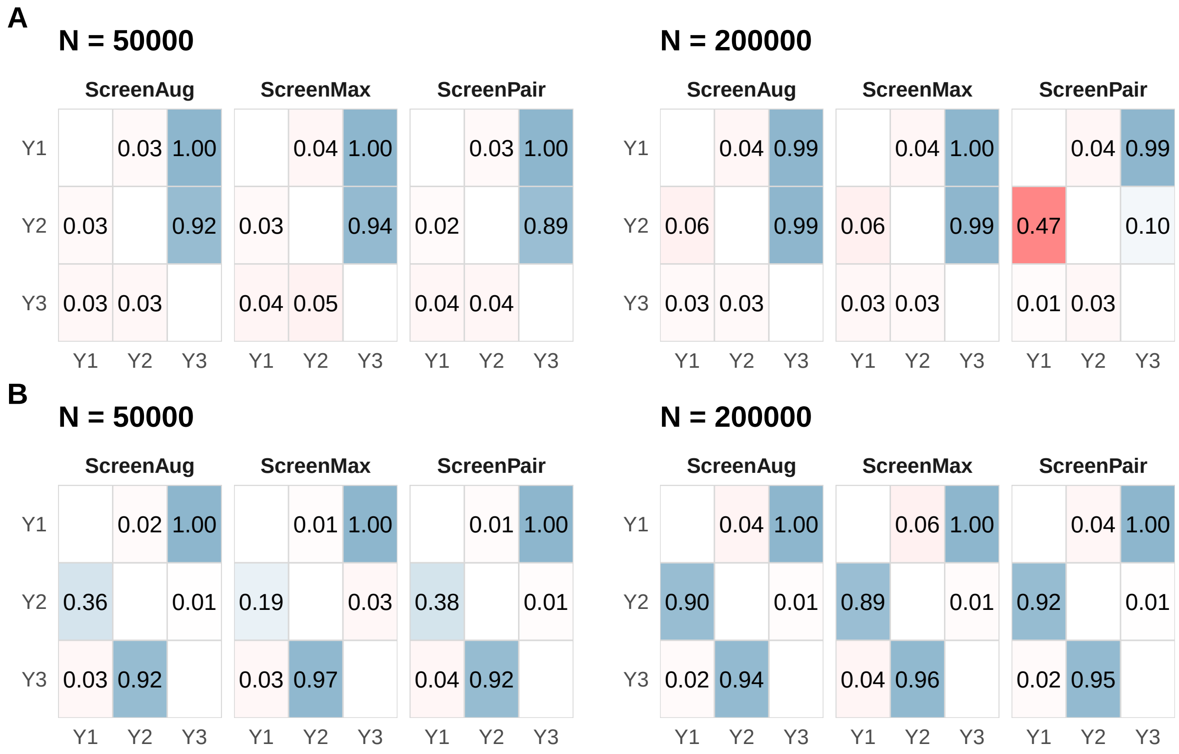

Figure Q. **Proportion of rejections across 200 replicates under latent confounder scenario.** Panel **A** corresponds to the acyclic setting in Fig. 2A, and Panel **B** corresponds to the cyclic setting in Fig. 2B. The left column shows results for $N = 50{,}000$, and the right column shows results for $N = 200{,}000$.

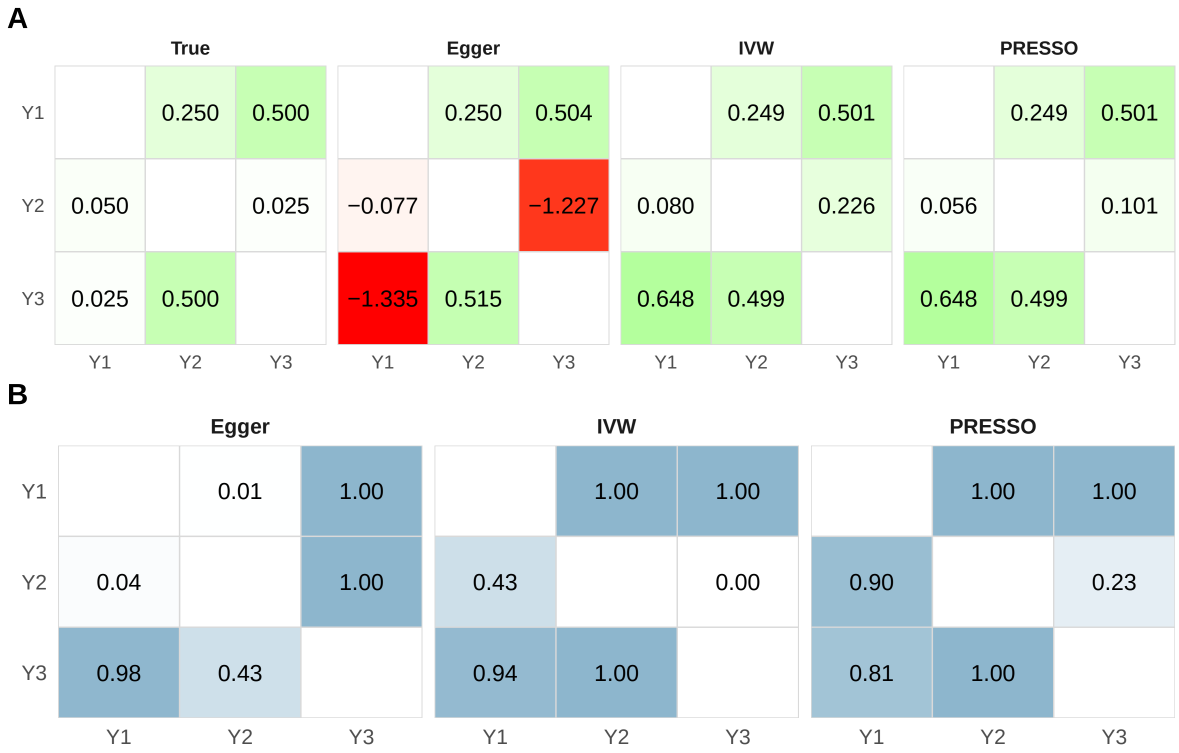

Figure R. **Results of univariable MR methods across 200 replicates under the cyclic setting in Fig. 2B with** $N = 50{,}000$**.** Panel **A** shows the true MR effects and average estimated MR effects ($\hat{\theta}_{ij}$) by MR-Egger, MR-IVW, and MR-PRESSO. Panel **B** shows the corresponding proportion of rejections for each exposure–outcome pair.

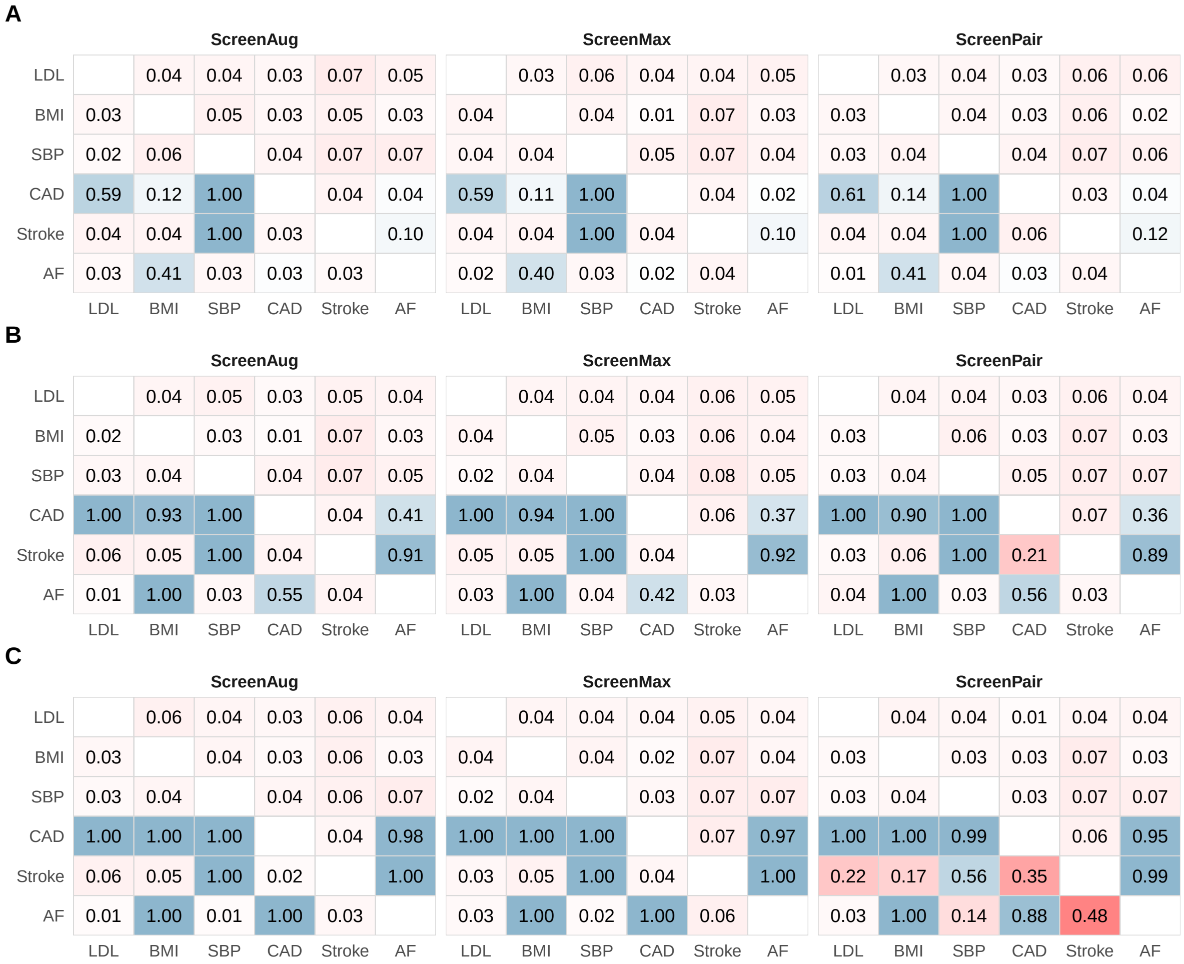

Figure S. **Proportion of rejections across 200 replicates in the simulation with real GWAS data under scaled direct-effect graphs.** Panels **A**–**C** correspond to scaling factors $\lambda = 0.1, 0.25$, and 0.5 respectively, applied to the true direct-effect matrix $\mathbf{G}$ in Figure 4A.

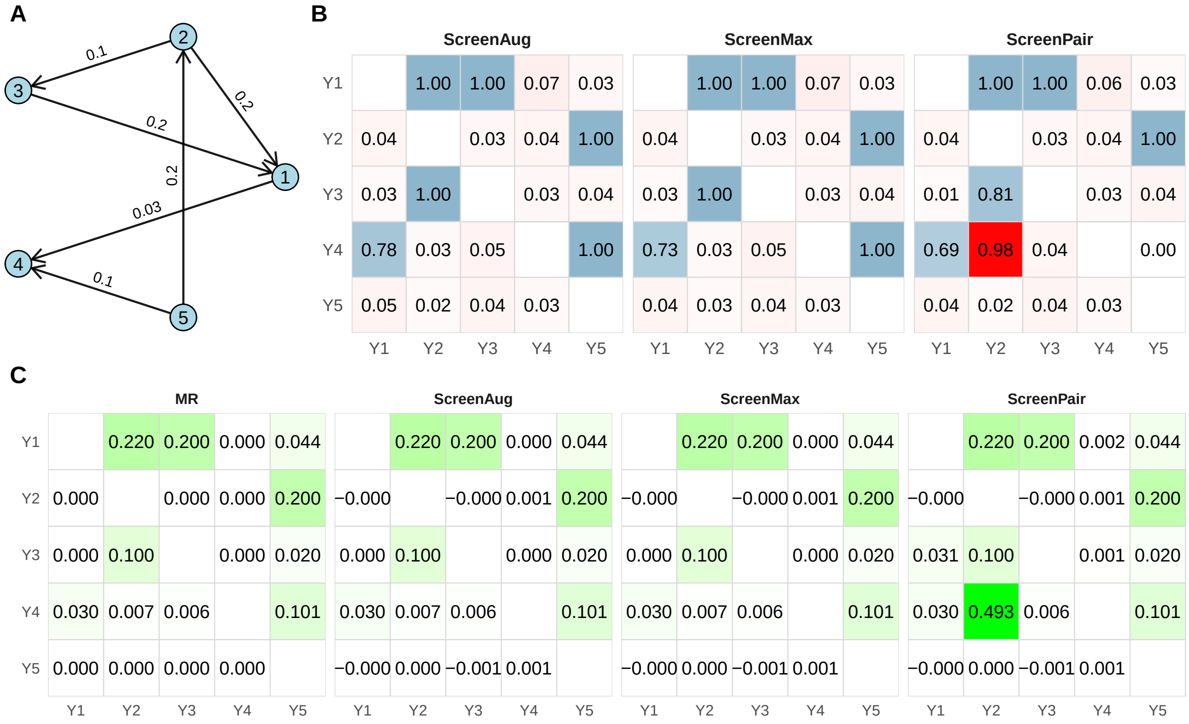

Figure T. **Simulation of the five-trait sensitivity analysis (Section 3.5).** Panel **A** shows the true direct-effect graph among five traits. Panel **B** shows the proportion of rejections for MR2G coupled with ScreenAug, ScreenMax, and ScreenPair. Panel **C** shows the true MR effects and average estimated effects by ScreenAug, ScreenMax, and ScreenPair.

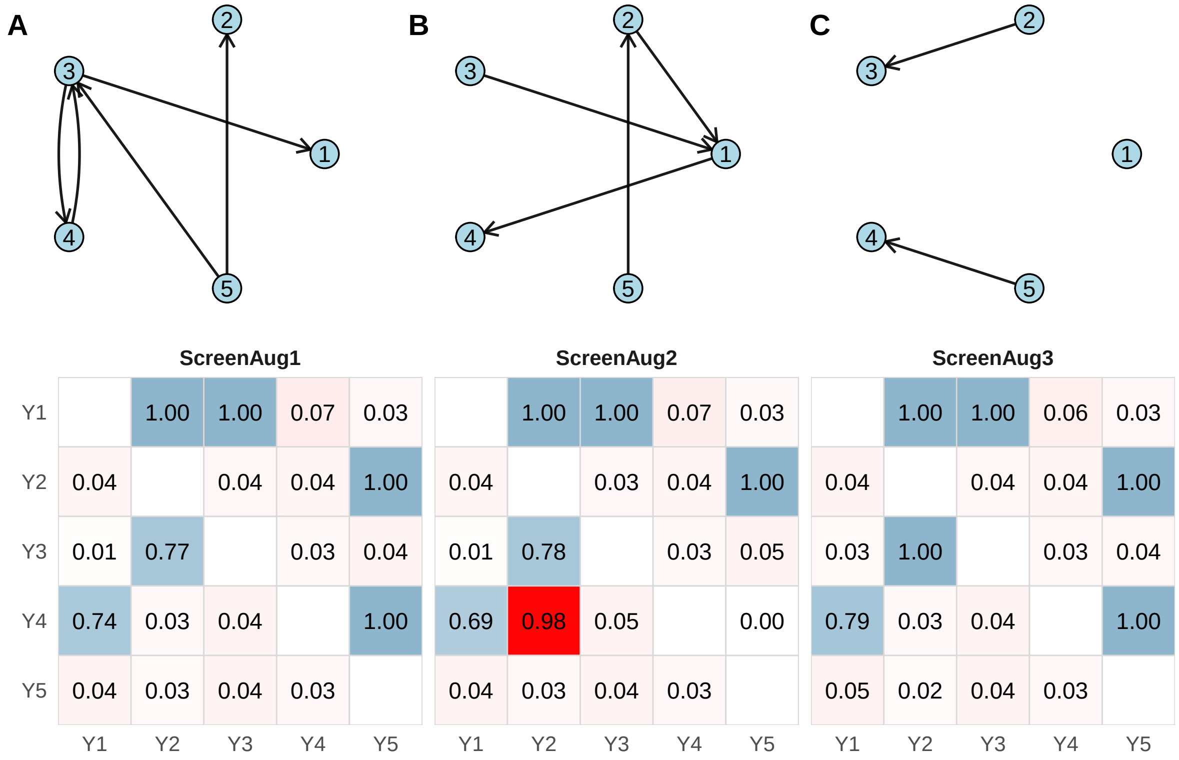

Figure U. **Sensitivity of ScreenAug to the reference graph used for IV augmentation.** Panels **A–C** correspond to three alternative reference graphs (ScreenAug1, ScreenAug2, and ScreenAug3, respectively) and the resulting proportion of rejections for ScreenAug under each misspecified graph.

2 Supplementary tables

Table A. **Root mean squared error for the simulation with GWAS summary statistics (Figure 2C in the main text).** Tables (a)–(c) show the root mean squared error of the estimated direct-effect graph by MR2G coupled with (a) ScreenAug, (b) ScreenPair and (c) ScreenMax across 200 replicates. All values are rounded to three decimal places.

	LDL	BMI	SBP	CAD	Stroke	AF
LDL		0.006	0.006	0.005	0.009	0.006
BMI	0.005		0.004	0.004	0.006	0.005
SBP	0.004	0.004		0.004	0.01	0.004
CAD	0.014	0.013	0.019		0.035	0.012
Stroke	0.013	0.013	0.012	0.013		0.01
AF	0.011	0.011	0.02	0.006	0.038	

(a) ScreenAug

	LDL	BMI	SBP	CAD	Stroke	AF
LDL		0.006	0.005	0.006	0.009	0.006
BMI	0.005		0.003	0.004	0.006	0.005
SBP	0.005	0.004		0.008	0.011	0.003
CAD	0.014	0.014	0.034		0.067	0.017
Stroke	0.268	0.139	0.486	0.639		0.07
AF	0.05	0.023	0.008	0.121	0.184	

(b) ScreenPair

	LDL	BMI	SBP	CAD	Stroke	AF
LDL		0.007	0.014	0.009	0.022	0.008
BMI	0.006		0.012	0.006	0.018	0.006
SBP	0.004	0.004		0.004	0.011	0.004
CAD	0.014	0.013	0.02		0.036	0.012
Stroke	0.014	0.013	0.012	0.014		0.01
AF	0.012	0.011	0.022	0.01	0.037	

(c) ScreenMax

Table B. GWAS summary data for the 15 traits used in real data analysis.

GWAS trait	ID in IEU data base	Reference
LDL	ebi-a-GCST002222	[2]
HDL	ebi-a-GCST002223	[2]
Triglycerides	ebi-a-GCST002216	[2]
Height	ieu-a-89	[3]
BMI	ieu-a-835	[4]
SBP	ukb-a-360	[5]
Fasting glucose	ebi-a-GCST000568	[6]
Smoke	ieu-b-25	[7]
Alcohol	ieu-b-73	[7]
CAD	ebi-a-GCST005195	[8]
Stroke	ebi-a-GCST005838	[9]
T2D	ieu-a-26	[10]
Asthma	ebi-a-GCST006862	[11]
AF	ebi-a-GCST006414	[12]
Alzheimer's disease	Downloaded from GWAS catalog (study ID: GCST007320)	[13]

Table C. **Runtime of MR2G (ScreenMax) under different simulation settings.** All runtimes are averaged across replicates and measured on a 36-core computing node with parallelization via `pbmclapply` in R.

Setting	Traits (T)	Avg. IVs per pair	Perturbations (B)	Avg. runtime (s)
Section 3.3.1	3	10	200	6
Section 3.5	5	12	200	21
Section 3.4.1	6	70	100	26

3 Supplementary methods

3.1 Theoretical results

3.1.1 Proof of Theorem 1

Theorem 1. Suppose Z_k is a valid IV for Y_j to Y_i, we have the ratio of total marginal effects as $\beta_{ik}/\beta_{jk} = w_{ij}/w_{jj} := \theta_{ij}$. Therefore, θ_{ij} is independent of Z_k and totally determined by $\mathbf{W}$ and thus by $\mathbf{G}$. We call θ_{ij} as the MR effect from Y_j to Y_i.

Proof. For a directed path $\boldsymbol{Q}_M$, we denote the effect of Q_1 to Q_M passing through $\boldsymbol{Q}_M$ as $E_{\boldsymbol{Q}_M}$, and we have $E_{\boldsymbol{Q}_M} = \prod_{l=1}^{M-1} E_l$, with E_l being the direct effect from Q_l to Q_{l+1} (i.e., element in $\boldsymbol{\Gamma}$ or $\mathbf{G}$). For two directed paths $\boldsymbol{Q}_M = (Q_1, \cdots, Q_M)$ and $\boldsymbol{Q}'_{M'} = (Q'_1, \cdots, Q'_{M'})$, if the last variable of $\boldsymbol{Q}_M$ (i.e., Q_M) and the first variable of $\boldsymbol{Q}'_{M'}$ (i.e., Q'_1) are the same trait, we can concatenate them as one direct path with $(M+M'-1)$ variables as $\boldsymbol{Q}^*_{M+M'-1} = (Q_1, \cdots, Q_M, \cdots, Q'_{M'})$. The effect of Q_1 to $Q'_{M'}$ through $\boldsymbol{Q}^*_{M+M'-1}$ is $E_{\boldsymbol{Q}^*_{M+M'-1}} = E_{\boldsymbol{Q}_M} \cdot E_{\boldsymbol{Q}'_{M'}}$.

Denote $\mathcal{Q} = \left\{\boldsymbol{Q}_M \middle| \boldsymbol{Q}_M = (Q_1, \cdots, Q_M), M \geq 3, Q_1 = Z_k, Q_M = Y_i\right\}$ as the set of all directed paths from Z_k to Y_i, here we have $M \geq 3$ as Z_k is a valid IV for Y_j to Y_i and therefore cannot have direct effect on Y_i. β_{ik} is the total marginal effect from Z_k to Y_i, we have

$$\beta_{ik} = \sum_{\boldsymbol{Q}_M \in \mathcal{Q}} E_{\boldsymbol{Q}_M}.$$

Since Z_k is a valid IV for Z_j to Z_i, for each $\boldsymbol{Q}_M \in \mathcal{Q}$, there is at least one variable in $\boldsymbol{Q}_M$ being Z_j. We denote Q_l as the first element in $\mathbf{Q}_M$ such that $Q_l = Z_j$, and we decompose $\boldsymbol{Q}_M$ into two sub-directed paths $\boldsymbol{Q}^{(1)}_l = (Q_1, \cdots, Q_l)$ and $\boldsymbol{Q}^{(2)}_{M-l+1} = (Q_l, \cdots, Q_M)$. We

have $E_{\boldsymbol{Q}_M} = E_{\boldsymbol{Q}_l^{(1)}} \cdot E_{\boldsymbol{Q}_{M-l+1}^{(2)}}$. Denote $\mathcal{Q}_1 = \{\boldsymbol{Q}_l^{(1)} | \boldsymbol{Q}_l^{(1)} = (Q_1, \cdots, Q_l), l \geq 2, Q_1 = Z_k, Q_l = Y_j, Q_2, \cdots, Q_{l-1} \neq Y_j\}$ as the set of all directed paths from Z_k to Y_j such that Y_j only appears once as the last variable, and $\mathcal{Q}_2 = \{\boldsymbol{Q}_{M-l+1}^{(2)} | \boldsymbol{Q}_{M-l+1}^{(2)} = (Q_l, \cdots, Q_M), M - l + 1 \geq 2, Q_l = Y_j, Q_M = Y_i\}$ as the set of all directed paths from Y_j to Y_i. Since each Q_M can be uniquely decomposed as two sub-directed paths in $\mathcal{Q}_1$ and $\mathcal{Q}_2$ respectively, and each pair of directed paths with the first from $\mathcal{Q}_1$ and the second from $\mathcal{Q}_2$ can be uniquely concatenated as one directed path in Q_M, we have

$$\beta_{ik} = \sum_{\boldsymbol{Q}_M \in \mathcal{Q}} E_{\boldsymbol{Q}_M} = \sum_{\boldsymbol{Q}_M \in \mathcal{Q}} E_{\boldsymbol{Q}_l^{(1)}} \cdot E_{\boldsymbol{Q}_{M-l+1}^{(2)}} = \sum_{\boldsymbol{Q}_l^{(1)} \in \mathcal{Q}_1} E_{\boldsymbol{Q}_l^{(1)}} \cdot \sum_{\boldsymbol{Q}_{M-l+1}^{(2)} \in \mathcal{Q}_2} E_{\boldsymbol{Q}_{M-l+1}^{(2)}}.$$

Denote $\mathcal{Q}' = \{\boldsymbol{Q}'_{M'} | \boldsymbol{Q}'_{M'} = (Q'_1, \cdots, Q'_{M'}), M' \geq 2, Q'_1 = Z_k, Q'_{M'} = Y_j\}$ as the set of all directed paths from Z_k to Y_j, we have the total marginal effect from Z_k to Y_j as

$$\beta_{jk} = \sum_{\boldsymbol{Q}'_{M'} \in \mathcal{Q}'} E_{\boldsymbol{Q}'_{M'}}.$$

Due to the possible existence of cycles, Y_j can appears multiple times in $\boldsymbol{Q}'_{M'}$. Denote $Q'_{l'}$ as the first Y_j in $\boldsymbol{Q}'_{M'}$, we decompose $\boldsymbol{Q}'_{M'}$ as two parts $\boldsymbol{Q}'^{(1)}_{l'} = (Q'_1, \cdots, Q'_{l'})$ and $\boldsymbol{Q}'^{(2)}_{M'-l'+1} = (Q'_{l'}, \cdots, Q'_{M'})$, we have $E_{\boldsymbol{Q}'_{M'}} = E_{\boldsymbol{Q}'^{(1)}_{l'}} \cdot E_{\boldsymbol{Q}'^{(2)}_{M'-l'+1}}$. Denote $\mathcal{Q}'_1 = \{\boldsymbol{Q}'^{(1)}_{l'} | \boldsymbol{Q}'^{(1)}_{l'} = (Q'_1, \cdots, Q'_{l'}), l' \geq 2, Q'_1 = Z_k, Q'_{l'} = Y_j, Q'_2, \cdots, Q'_{l'-1} \neq Y_j\}$ as the set of all directed paths from Z_k to Y_j such that Y_j only appears once as the last variable, and $\mathcal{Q}'_2 = \{\boldsymbol{Q}'^{(2)}_{M'-l'+1} | \boldsymbol{Q}'^{(2)}_{M'-l'+1} = (Q'_{l'}, \cdots, Q'_{M'}), M' - l' + 1 \geq 3$ or $M' - l' + 1 = 1, Q'_{l'} = Y_j, Q'_{M'} = Y_j\}$ as the set of all directed paths from Y_j to Y_j (i.e., itself); note that, when $M' - l' - 1 = 1$ (i.e., the directed path contains only one variable Y_j), we denote the effect $E_{\boldsymbol{Q}'_{M'}} = 1$. Since each $Q'_{M'}$ can be uniquely decomposed as two sub-directed paths in $\mathcal{Q}'_1$ and $\mathcal{Q}'_2$ respectively, and each pair of directed paths with the first from $\mathcal{Q}'_1$ and the second from $\mathcal{Q}'_2$ can be uniquely concatenated as one directed path in $Q'_{M'}$, we have

$$\beta_{jk} = \sum_{\boldsymbol{Q}'_{M'} \in \mathcal{Q}'} E_{\boldsymbol{Q}'_{M'}} = \sum_{\boldsymbol{Q}'_{M'} \in \mathcal{Q}'} E_{\boldsymbol{Q}'^{(1)}_{l'}} \cdot E_{\boldsymbol{Q}'^{(2)}_{M'-l'+1}} = \sum_{\boldsymbol{Q}'^{(1)}_{l'} \in \mathcal{Q}'_1} E_{\boldsymbol{Q}'^{(1)}_{l'}} \cdot \sum_{\boldsymbol{Q}'^{(2)}_{M'-l'+1} \in \mathcal{Q}'_2} E_{\boldsymbol{Q}'^{(2)}_{M'-l'+1}}.$$

Note that, the two sets $\mathcal{Q}_1$ and $\mathcal{Q}_1'$ are identical, so $\sum_{\boldsymbol{Q}_l^{(1)}\in\mathcal{Q}_1} E_{\boldsymbol{Q}_l^{(1)}} = \sum_{\boldsymbol{Q}_{l'}^{\prime(1)}\in\mathcal{Q}_1'} E_{\boldsymbol{Q}_{l'}^{\prime(1)}}$. Furthermore, according to the definition of $\mathcal{Q}_2$ and $\mathcal{Q}_2'$, we have $\sum_{\boldsymbol{Q}_{M-l+1}^{(2)}\in\mathcal{Q}_2} E_{\boldsymbol{Q}_{M-l+1}^{(2)}}$ being the marginal total effect of Y_j to Y_i, i.e. w_{ij}; and $\sum_{\boldsymbol{Q}_{M'-l'+1}^{\prime(2)}\in\mathcal{Q}_2'} E_{\boldsymbol{Q}_{M'-l'+1}^{\prime(2)}}$ being the marginal total effect of Y_j to Y_j itself, i.e. w_{jj}. Therefore, we have the ratio of the two total marginal effects as

$$\frac{\beta_{ik}}{\beta_{jk}} = \frac{\sum_{\boldsymbol{Q}_{M-l+1}^{(2)}\in\mathcal{Q}_2} E_{\boldsymbol{Q}_{M-l+1}^{(2)}}}{\sum_{\boldsymbol{Q}_{M'-l'+1}^{\prime(2)}\in\mathcal{Q}_2'} E_{\boldsymbol{Q}_{M'-l'+1}^{\prime(2)}}} = \frac{w_{ij}}{w_{jj}} := \theta_{ij}.$$

□

3.1.2 Asymptotic property of the estimated causal network

In this section, we establish the asymptotic property of the estimated causal network $\widehat{\mathbf{G}}$ under some suitable conditions. Denote vec($\mathbf{G}$) and vec($\widehat{\mathbf{G}}$) as vectors of length $T \times T$, which are the vectorizations of the matrices $\mathbf{G}$ and $\widehat{\mathbf{G}}$, respectively. The GWAS sample sizes for the T traits are $N_1, \cdots, N_T$, and denote $N = \min(N_1, \cdots, N_T)$.

Assumption 3. (Orders of the variances and sample sizes.) All $\hat{\beta}_{ik}$'s jointly follow a multivariate normal distribution, all of the sample sizes $N_1, \cdots, N_T$ are of order $O(N)$, and all of the standard errors SE($\hat{\beta}_{ik}$)'s are of order $O(N)$ as well.

Assumption 4. (Plurality valid condition.) For each ordered pair of traits (Y_j, Y_i), denote $\mathcal{M}_{ij}^0$ as the set of true valid IVs in $\mathcal{M}_{ij}$ (i.e., candidate IV set obtained with ScreenMax). For any $B \subseteq \mathcal{M}_{ij}$ and $|B| = \mathcal{M}_{ij}^0$, if $B \neq \mathcal{M}_{ij}^0$, then the ratios $\{\beta_{ik}/\beta_{jk}, k \in \mathcal{M}_{ij} \setminus \mathcal{M}_{ij}^0\}$ are not all equal. Similarly, denote $\mathcal{A}_{ij}^0$ as the set of true valid IVs in $\mathcal{A}_{ij} := \mathcal{M}_{ij} \cup \{Z_k's\}$ (i.e., candidate IV set obtained with ScreenAug). For any $B \subseteq \mathcal{A}_{ij}$ and $|B| = \mathcal{A}_{ij}^0$, if $B \neq \mathcal{A}_{ij}^0$, then the ratios $\{\beta_{ik}/\beta_{jk}, k \in \mathcal{A}_{ij} \setminus \mathcal{A}_{ij}^0\}$ are not all equal.

Theorem 2. Under Assumptions 1 to 4, we have vec($\widehat{\mathbf{G}}$) is a consistent estimator of vec($\mathbf{G}$) and follows an asymptotic multivariate normal distribution, such that

$$\sqrt{N}\left(\text{vec}(\widehat{\mathbf{G}}) - \text{vec}(\mathbf{G})\right) \xrightarrow{d} N\left(\mathbf{0}, \mathbf{V}\right),$$

here $\mathbf{V} \in \mathbb{R}^{(T\times T)\times(T\times T)}$ is the asymptotic covariance matrix.

Proof. For each ordered pair of traits (Y_j, Y_i), with Assumptions 3 and 4, following Theorem 1 in [14] we have the sets of valid IVs $\mathcal{M}_{ij}^0$ and $\mathcal{A}_{ij}^0$ can be consistently selected. Therefore, the estimated MR effect $\hat{\theta}_{ij}$ with ScreenAug is a consistent estimator of the true MR effect θ_{ij}, and $\hat{\theta}_{ij}$ follows an asymptotic normal distribution. Following similar arguments in the proof of Corollary 1 in [1], each $\hat{\theta}_{ij}$ is asymptotically equivalent to a linear function of $\hat{\beta}_{ik}$'s and $\hat{\beta}_{jk}$'s. Therefore, with Assumption 3 that all $\hat{\beta}_{ik}$'s follow an joint multivariate normal distribution, we have all $\hat{\theta}_{ij}$'s follow an asymptotic multivariate normal distribution with means as θ_{ij}'s and covariance matrix denoted by $\mathbf{W}$, which can be expressed as $\sqrt{N}\left(\text{vec}(\widehat{\boldsymbol{\Theta}}) - \text{vec}(\boldsymbol{\Theta})\right) \xrightarrow{d} N(\mathbf{0}, \mathbf{W})$. From Algorithm 1, $\widehat{\mathbf{G}}$ can be obtained from $\widehat{\boldsymbol{\Theta}}$ through matrix inversion and multiplication, which are continuous and differentiable mappings [15]. With Delta method [16], we have that $\text{vec}(\widehat{\mathbf{G}})$ also follows an asymptotic normal distribution, denoted as $\sqrt{N}\left(\text{vec}(\widehat{\mathbf{G}}) - \text{vec}(\mathbf{G})\right) \xrightarrow{d} N(\mathbf{0}, \mathbf{V})$. □

Note that, although Theorem 2 provides theoretical support for MR2G, due to the complicated form of the asymptotic covariance matrix $\mathbf{V}$ (which is not explicitly calculated here), inference of $\widehat{\mathbf{G}}$ is based on data-perturbation throughout the paper.

3.2 Connection between MVMR and MR2G direct effects

In this section, we formally establish the connection between the direct effects estimated by MVMR and the direct-effect matrix $\mathbf{G}$ recovered by MR2G. We show that, when all other traits are included as exposures, the MVMR estimand for a given outcome coincides exactly with the corresponding row of $\mathbf{G}$, even in the presence of cycles.

3.2.1 Setup and notation

Consider T traits $Y_1, \ldots, Y_T$ related by the recursive model in Eq. (5) of the main text, with equilibrium

$$\mathbf{Y} = \mathbf{G}\mathbf{Y} + \boldsymbol{\Gamma}\mathbf{Z} + \boldsymbol{\epsilon}, \tag{1}$$

so that the reduced form is $\mathbf{Y} = (\mathbf{I}-\mathbf{G})^{-1}\mathbf{\Gamma}\mathbf{Z}+(\mathbf{I}-\mathbf{G})^{-1}\boldsymbol{\epsilon}$. Define $\mathbf{W} = (\mathbf{I}-\mathbf{G})^{-1}$, and the matrix of SNP–trait associations in the reduced form is $\mathbf{B} = \mathbf{W}\mathbf{\Gamma}$, i.e., $\beta_{ik} = \sum_{l=1}^{T} w_{il}\gamma_{lk}$.

3.2.2 MVMR estimand

In a standard MVMR analysis with outcome Y_i and exposures $\{Y_j : j \in \mathcal{E}\}$, one assumes the relationship between outcome's SNP–trait associations and the exposures' SNP–trait association for a valid IV Z_k is:

$$\beta_{ik} = \sum_{j\in\mathcal{E}} \alpha_j \, \beta_{jk}. \tag{2}$$

Recall that in the reduced form, $\beta_{ik} = \sum_{l=1}^{T} w_{il}\,\gamma_{lk}$. If all IVs are valid, meaning each Z_k has a non-zero direct effect γ_{lk} only on traits in $\mathcal{E}$ (i.e., $\gamma_{lk} = 0$ for $l \notin \mathcal{E}$), then the sums collapse to only include traits in $\mathcal{E}$:

$$\beta_{ik} = \sum_{l\in\mathcal{E}} w_{il}\,\gamma_{lk}, \qquad \beta_{jk} = \sum_{l\in\mathcal{E}} w_{jl}\,\gamma_{lk}, \quad j \in \mathcal{E}. \tag{3}$$

Substituting into (2) gives

$$\sum_{l\in\mathcal{E}} w_{il}\,\gamma_{lk} = \sum_{j\in\mathcal{E}} \alpha_j \sum_{l\in\mathcal{E}} w_{jl}\,\gamma_{lk} = \sum_{l\in\mathcal{E}} \left(\sum_{j\in\mathcal{E}} \alpha_j \, w_{jl} \right) \gamma_{lk}.$$

For this to hold for all valid IVs, the coefficients of γ_{lk} must match for each $l \in \mathcal{E}$:

$$w_{il} = \sum_{j\in\mathcal{E}} \alpha_j \, w_{jl}, \quad \forall\, l \in \mathcal{E}.$$

Writing this in matrix form across all $l \in \mathcal{E}$:

$$\underbrace{(w_{il})_{l\in\mathcal{E}}}_{\mathbf{w}_{i,\mathcal{E}}^{\top}} = \underbrace{(\alpha_j)_{j\in\mathcal{E}}}_{\boldsymbol{\alpha}^{\top}} \underbrace{(w_{jl})_{j,l\in\mathcal{E}}}_{\mathbf{W}_{\mathcal{E},\mathcal{E}}}, \tag{4}$$

i.e., $\mathbf{w}_{i,\mathcal{E}}^\top = \boldsymbol{\alpha}^\top \mathbf{W}_{\mathcal{E},\mathcal{E}}$. Assuming sufficient IVs so that $\mathbf{W}_{\mathcal{E},\mathcal{E}}$ is invertible, the MVMR estimand is

$$\boldsymbol{\alpha}^\top = \mathbf{w}_{i,\mathcal{E}}^\top \mathbf{W}_{\mathcal{E},\mathcal{E}}^{-1}. \tag{5}$$

3.2.3 Equivalence when all traits are included as exposures

We now show that when $\mathcal{E} = \{1, \ldots, T\} \setminus \{i\}$ (i.e., all other traits serve as exposures for outcome Y_i), the MVMR estimand $\boldsymbol{\alpha}$ equals $\mathbf{g}_{i,-i}$, the i-th row of $\mathbf{G}$ restricted to columns $j \neq i$.

Proposition 1. Let $\mathbf{W} = (\mathbf{I} - \mathbf{G})^{-1}$ with $g_{ii} = 0$ for all i. For any outcome Y_i with all remaining traits as exposures ($\mathcal{E} = \{1, \ldots, T\} \setminus \{i\}$), the MVMR estimand satisfies

$$\boldsymbol{\alpha}^\top = \mathbf{w}_{i,-i}^\top \mathbf{W}_{-i,-i}^{-1} = \mathbf{g}_{i,-i}^\top,$$

where $\mathbf{g}_{i,-i} = (g_{ij})_{j \neq i}$ is the i-th row of $\mathbf{G}$ with the diagonal entry removed, expressed as a column vector.

Proof. Since $\mathbf{W} = (\mathbf{I} - \mathbf{G})^{-1}$, we have $\mathbf{W}(\mathbf{I} - \mathbf{G}) = \mathbf{I}$. The i-th row of this identity gives, for each j:

$$\sum_{l=1}^{T} w_{il}(\delta_{lj} - g_{lj}) = \delta_{ij}.$$

For $j \neq i$, this yields

$$w_{ij} = w_{ii}\, g_{ij} + \sum_{l \neq i} w_{il}\, g_{lj}, \tag{6}$$

or equivalently, in matrix form,

$$\mathbf{w}_{i,-i}^\top = w_{ii}\, \mathbf{g}_{i,-i}^\top + \mathbf{w}_{i,-i}^\top\, \mathbf{G}_{-i,-i}. \tag{7}$$

Rearranging,

$$\mathbf{w}_{i,-i}^{\top}(\mathbf{I} - \mathbf{G}_{-i,-i}) = w_{ii}\, \mathbf{g}_{i,-i}^{\top}. \tag{8}$$

For $j = i$ (using $g_{ii} = 0$),

$$w_{ii} = 1 + \sum_{l \neq i} w_{il}\, g_{li} = 1 + \mathbf{w}_{i,-i}^{\top}\, \mathbf{g}_{-i,i}. \tag{9}$$

Next, we compute $\mathbf{W}_{-i,-i}^{-1}$ via the Schur complement. Partition $\mathbf{W}$ as

$$\mathbf{W} = \begin{pmatrix} w_{ii} & \mathbf{w}_{i,-i}^{\top} \\ \mathbf{w}_{-i,i} & \mathbf{W}_{-i,-i} \end{pmatrix}, \quad \mathbf{W}^{-1} = \mathbf{I} - \mathbf{G} = \begin{pmatrix} 1 & -\mathbf{g}_{i,-i}^{\top} \\ -\mathbf{g}_{-i,i} & \mathbf{I} - \mathbf{G}_{-i,-i} \end{pmatrix}.$$

By the standard block-inversion formula, the inverse of the lower-right block $\mathbf{W}_{-i,-i}$ is

$$\mathbf{W}_{-i,-i}^{-1} = (\mathbf{I} - \mathbf{G}_{-i,-i}) - \frac{(-\mathbf{g}_{-i,i})(-\mathbf{g}_{i,-i}^{\top})}{1} = (\mathbf{I} - \mathbf{G}_{-i,-i}) - \mathbf{g}_{-i,i}\, \mathbf{g}_{i,-i}^{\top}. \tag{10}$$

Substituting (10) into the MVMR estimand (5) with $\mathcal{E} = \{1, \ldots, T\} \setminus \{i\}$:

$$\begin{aligned} \boldsymbol{\alpha}^{\top} &= \mathbf{w}_{i,-i}^{\top}\, \mathbf{W}_{-i,-i}^{-1} \\ &= \mathbf{w}_{i,-i}^{\top} \left[(\mathbf{I} - \mathbf{G}_{-i,-i}) - \mathbf{g}_{-i,i}\, \mathbf{g}_{i,-i}^{\top}\right] \\ &= \mathbf{w}_{i,-i}^{\top}(\mathbf{I} - \mathbf{G}_{-i,-i}) - \mathbf{w}_{i,-i}^{\top}\mathbf{g}_{-i,i}\, \mathbf{g}_{i,-i}^{\top}. \end{aligned} \tag{11}$$

Using Eq. (8), the first term equals $w_{ii}\, \mathbf{g}_{i,-i}^{\top}$. Substituting:

$$\begin{aligned} \boldsymbol{\alpha}^{\top} &= w_{ii}\, \mathbf{g}_{i,-i}^{\top} - \mathbf{w}_{i,-i}^{\top}\mathbf{g}_{-i,i}\, \mathbf{g}_{i,-i}^{\top} \\ &= \left(w_{ii} - \mathbf{w}_{i,-i}^{\top}\mathbf{g}_{-i,i}\right) \mathbf{g}_{i,-i}^{\top}. \end{aligned} \tag{12}$$

From Eq. (9), the scalar factor is $w_{ii} - \mathbf{w}_{i,-i}^\top \mathbf{g}_{-i,i} = 1$. Therefore,

$$\boldsymbol{\alpha}^\top = \mathbf{g}_{i,-i}^\top.$$

□

Remark 1. Proposition 1 shows that MVMR with all other traits as exposures targets the same direct effects as the matrix $\mathbf{G}$ recovered by MR2G. This equivalence holds regardless of whether $\mathbf{G}$ encodes a DAG or contains cycles.

Remark 2 (Partial exposure set). When only a strict subset $\mathcal{E} \subsetneq \{1, \dots, T\} \setminus \{i\}$ of traits is included as exposures, the MVMR estimand $\boldsymbol{\alpha}^\top = \mathbf{w}_{i,\mathcal{E}}^\top \mathbf{W}_{\mathcal{E},\mathcal{E}}^{-1}$ generally does *not* equal the corresponding entries of $\mathbf{g}_{i,-i}$. Traits omitted from $\mathcal{E}$ act as unmeasured mediators, and their indirect effects are absorbed into the estimated coefficients for the included exposures.

3.3 Additional analyses for the simulation with three traits

3.3.1 Setup in the main text

In the main text, we generated individual-level data based on the following marginal model,

$$\mathbf{Y} = (\mathbf{I} - \mathbf{G})^{-1}\boldsymbol{\Gamma}\mathbf{Z} + (\mathbf{I} - \mathbf{G})^{-1}\boldsymbol{\epsilon}, \tag{13}$$

where $\mathbf{Y}$ was a $3 \times N$ matrix, $\mathbf{Z}$ was a $30 \times N$ matrix of 30 SNPs, each independently generated from a binomial distribution with a minor allele frequency of 0.3. $\boldsymbol{\Gamma}$ was a 3×30 matrix, in which the first 8 SNPs had non-zero effects γ_{1k} only on Y_1, the next 7 SNPs had non-zero effects γ_{2k} only on Y_2, and the remaining 15 SNPs had non-zero effects γ_{3k} only on Y_3, and the non-zero effect sizes (γ_{ik}) were drawn from a uniform distribution over $(-0.2, -0.1) \cup (0.1, 0.2)$. The random errors $\boldsymbol{\epsilon}$ were drawn from $\mathcal{MVN}(0, \boldsymbol{\Sigma}_e)$, where $\boldsymbol{\Sigma}_e$ had an AR(1) structure with correlation 0.4 to reflect the unmeasured confounder effect. We considered $\mathbf{G} = \left(\begin{smallmatrix} 0 & 0 & 0.2 \\ 0 & 0 & 0.1 \\ 0 & 0 & 0 \end{smallmatrix}\right)$, and $\mathbf{G} = \left(\begin{smallmatrix} 0 & 0 & 0.5 \\ 0.05 & 0 & 0 \\ 0 & 0.5 & 0 \end{smallmatrix}\right)$ in Fig. 2a and Fig. 2b in the

main text respectively.

3.3.2 Varying AR(1) residual correlation

In the main text, we used an AR(1) structure with correlation parameter $\rho = 0.4$ for the residual covariance matrix $\boldsymbol{\Sigma}_e$. To assess the sensitivity of MR2G to the strength of residual correlation, we repeated the simulation under $\rho \in \{0.1, 0.7\}$, keeping all other settings identical to those described in Section 3.3.1. A smaller ρ corresponds to weaker confounding, whereas a larger ρ implies stronger confounding among the traits. We considered both graph structures: the acyclic graph $\mathbf{G} = \begin{pmatrix} 0 & 0 & 0.2 \\ 0 & 0 & 0.1 \\ 0 & 0 & 0 \end{pmatrix}$ (Fig. 2A in the main text) and the cyclic graph $\mathbf{G} = \begin{pmatrix} 0 & 0 & 0.5 \\ 0.05 & 0 & 0 \\ 0 & 0.5 & 0 \end{pmatrix}$ (Fig. 2B in the main text).

Figs. L and M present the results. Across both values of ρ and both graph structures, ScreenMax and ScreenAug maintain stable performance with well-controlled type-I error. In particular, for the acyclic setting (Panel A), ScreenPair exhibits inflated type-I error for $Y_1 \to Y_2$ when $N = 200{,}000$, consistent with the phenomenon observed in the main text under $\rho = 0.4$: as sample size increases, SNPs in $\boldsymbol{Z}_3$ become significantly associated with Y_1 and Y_2, and ScreenPair incorrectly selects them as IVs for Y_1 due to their stronger marginal associations with Y_1 than with Y_2 (as $g_{13} = 0.2 > g_{23} = 0.1$), introducing correlated pleiotropy and inflating false positives. In contrast, ScreenAug and ScreenMax correctly assign these SNPs only to Y_3 and maintain well-controlled type-I error across all settings. For the cyclic setting (Fig. 2B), all three screening methods perform similarly, again consistent with the main text. At $N = 50{,}000$, ScreenMax has slightly lower power for detecting $Y_1 \to Y_2$ (e.g., 0.49 under $\rho = 0.7$) compared to ScreenAug and ScreenPair (both 0.79), because ScreenMax uses fewer IVs. ScreenAug recovers this power by augmenting the IV set with valid IVs from $\boldsymbol{Z}_3$. These results confirm that the conclusions from the main text are robust to varying levels of residual correlation.

3.3.3 Varying genetic effects and network edge strengths

The data-generating setup follows Section 3.3.1, with two modifications. First, we used weaker direct-effect graphs. For the acyclic graph (Figure 2A), we set $g_{13} = 0.1$ and

$g_{23} = 0.05$ (halved from the main text values of 0.2 and 0.1). For the cyclic graph (Figure 2B), we set $g_{21} = 0.05$, $g_{32} = 0.1$, and $g_{13} = 0.1$. Second, we controlled the per-trait SNP heritability h_g^2 to a pre-specified target value $h^2_{\text{target}} \in \{0.05, 0.10, 0.20\}$. Initial genetic effect sizes (γ_{ik}) were drawn from Uniform$(-0.06, -0.03) \cup (0.03, 0.06)$. The reduced-form genetic component $(\mathbf{I} - \mathbf{G})^{-1}\mathbf{\Gamma Z}$ and the noise component $(\mathbf{I} - \mathbf{G})^{-1}\boldsymbol{\epsilon}$ were computed, and the genetic component was subsequently rescaled trait-by-trait so that the realized heritability matched h^2_{target}. Concretely, for trait Y_i, letting $\hat{\sigma}^2_{G,i}$ and $\hat{\sigma}^2_{E,i}$ denote the empirical variances of the genetic and noise contributions respectively, the scaling factor was

$$s_i = \sqrt{\frac{h^2_{\text{target}}}{1 - h^2_{\text{target}}} \cdot \frac{\hat{\sigma}^2_{E,i}}{\hat{\sigma}^2_{G,i}}}.$$

Other settings were kept the same as in Section 3.3.1.

Figs. N to P presents the results across $h^2_{\text{target}} \in \{0.05, 0.10, 0.20\}$ and both graph structures. Overall, the results are consistent with the conclusions from the main text. Across all heritability levels and both graph structures, ScreenMax and ScreenAug maintain well-controlled type-I error and produce reliable estimates of the direct-effect network. For the acyclic setting (Panel A), when $h^2_{\text{target}} = 0.05$ or 0.10, the weaker genetic effects and edge effects prevent SNPs in $\boldsymbol{Z}_3$ (those directly associated with Y_3) reaching genome-wide significance for Y_1 or Y_2 even at $N = 200{,}000$, and therefore ScreenPair does not incorrectly select them as IVs for Y_1 when inferring $Y_1 \to Y_2$. As a result, ScreenPair maintains well-controlled type-I error at these lower heritability levels. However, when $h^2_{\text{target}} = 0.20$, the stronger genetic effects allow SNPs in $\boldsymbol{Z}_3$ to pass the significance threshold for marginal associations with Y_1 at $N = 200{,}000$, and ScreenPair again exhibits inflated type-I error for $Y_1 \to Y_2$, consistent with the phenomenon observed in the main text.

3.3.4 Explicit latent confounder

The AR(1) residual correlation structure used in the main text captures confounding through correlated error terms, but does not explicitly model a latent confounder that enters upstream and propagates through the causal network. To evaluate the robustness

of the IV screening strategies under more realistic structural confounding, we augmented the data-generating process with an explicit latent confounder U.

Specifically, we expanded the structural model by treating U as a fourth variable in the system alongside Y_1, Y_2, Y_3. The augmented direct-effect matrix $\mathbf{G}^*$ is a 4×4 matrix of the form

$$\mathbf{G}^* = \begin{pmatrix} \mathbf{G} & \boldsymbol{\delta} \\ \mathbf{0}^\top & 0 \end{pmatrix},$$

where the upper-left $3{\times}3$ block equals the original direct-effect matrix $\mathbf{G}$ among (Y_1, Y_2, Y_3), $\boldsymbol{\delta} = (0.5, 0.5, 0.5)^\top$ encodes the direct causal effect of U on each observable trait, and the last row is zero, so that U acts as an unobserved confounder variable of all three traits.

The genetic effect matrix $\boldsymbol{\Gamma}^*$ is 30×4: the first three columns are identical to the original $\boldsymbol{\Gamma}$ (SNPs 1–8 affect Y_1, SNPs 9–15 affect Y_2, SNPs 16–30 affect Y_3), and the fourth column assigns non-zero effects on U to 7 SNPs randomly selected from all 30 SNPs, with effect sizes drawn from Uniform$(-0.2, -0.1) \cup (0.1, 0.2)$. Because some of these 7 SNPs already have non-zero effects on one of Y_1, Y_2, or Y_3, they become horizontally pleiotropic through U. The random errors $\boldsymbol{\epsilon}^* = (\epsilon_1, \epsilon_2, \epsilon_3, \epsilon_U)^\top$ were drawn independently from $\mathcal{N}(0, 1)$, since all confounding is now encoded structurally through U. The reduced-form model for the augmented system is

$$\mathbf{Y}^* = (\mathbf{I} - \mathbf{G}^*)^{-1} \boldsymbol{\Gamma}^{*\top} \mathbf{Z} + (\mathbf{I} - \mathbf{G}^*)^{-1} \boldsymbol{\epsilon}^*, \tag{14}$$

where $\mathbf{Y}^* = (Y_1, Y_2, Y_3, U)^\top$ is the $4{\times}N$ matrix. Only Y_1, Y_2, and Y_3 are observed, and U is treated as an unobserved latent confounder. GWAS summary statistics and IV screening are performed using only the three observable traits. The two direct-effect graphs $\mathbf{G}$ (Fig. 2a and Fig. 2b) and all other settings were kept the same as in Section 3.3.1.

Fig. Q presents the results. Overall, the results are consistent with the conclusions from the main text. For the acyclic setting (Panel A), ScreenAug and ScreenMax maintain well-controlled type-I error across both sample sizes. In contrast, ScreenPair exhibits inflated type-I error for $Y_1 \to Y_2$ at $N = 200{,}000$ (0.47), and lower power for $Y_3 \to Y_1$ (0.10). For the cyclic setting (Panel B), all three screening methods maintain well-

controlled type-I error across both sample sizes, consistent with the main text. At $N =$ 50,000, ScreenMax has slightly lower power for detecting $Y_2 \to Y_1$ (0.19) compared to ScreenAug (0.36) and ScreenPair (0.38), as ScreenMax uses fewer IVs.

3.3.5 Comparison with conventional UVMR methods

To illustrate the advantage of MR2G over conventional UVMR methods in the network setting, we applied three widely used UVMR approaches, inverse-variance weighted (IVW) [17], MR-Egger [18], and MR-PRESSO [19], to the cyclic graph scenario (Fig. 2b in the main text) with sample size $N = 50{,}000$.

For each ordered pair of traits (Y_j, Y_i), we selected SNPs that were genome-wide significant ($p < 5 \times 10^{-8}$) for the exposure Y_j as candidate IVs, following the standard practice in conventional UVMR analyses. We then applied IVW, MR-Egger, and MR-PRESSO to estimate the causal effect of Y_j on Y_i. This was repeated for all six ordered pairs among the three traits across 200 simulation replicates.

It is important to note that conventional UVMR methods estimate the *MR effect* θ_{ij} (i.e., the ratio w_{ij}/w_{jj}), which reflects both direct and indirect causal effects propagated through the network. In contrast, MR2G further recovers the *direct effect* g_{ij} by leveraging the analytical relationship between $\boldsymbol{\Theta}$ and $\mathbf{G}$ (Eq. 7 in the main text). Consequently, even if UVMR methods correctly estimate the MR effects, they cannot distinguish direct from indirect effects without an additional network recovery step.

Fig. R presents the results. Panel A shows the true MR effects alongside the average estimates from MR-Egger, MR-IVW, and MR-PRESSO. In the cyclic graph $Y_1 \to Y_2 \to Y_3 \to Y_1$, all six MR effects θ_{ij} are non-zero because every pair of traits is connected by at least an indirect path. For example, although there is no direct edge from Y_2 to Y_1, the indirect path $Y_2 \to Y_3 \to Y_1$ yields a true MR effect of $\theta_{12} = 0.25$ $(= 0.5 \times 0.5)$.

First, MR-Egger produces severely biased estimates for certain pairs (e.g., $\hat{\theta} = -1.33$ for $Y_1 \to Y_3$ and $\hat{\theta} = -1.23$ for $Y_3 \to Y_2$), and correspondingly low power for $Y_1 \to Y_2$ (0.01) and $Y_2 \to Y_1$ (0.04). This is consistent with the well-known sensitivity of MR-Egger to allele harmonization issues [20]. Second, for pairs such as $Y_3 \to Y_1$ and $Y_2 \to Y_3$,

all three methods produce reasonable estimates and achieve high power, because the candidate IVs for the exposure (e.g., SNPs in $\boldsymbol{Z}_3$ for $Y_3 \to Y_1$) are largely valid — they affect the exposure directly and reach the outcome only through the exposure. Third, MR-PRESSO performs well for $Y_1 \to Y_2$: the candidate IV set contains SNPs from $\boldsymbol{Z}_1 \cup \boldsymbol{Z}_3$ (valid) along with a few SNPs from $\boldsymbol{Z}_2$ that sometimes also reach genome-wide significance for Y_1. MR-PRESSO is able to detect these pleiotropic outliers and remove them, yielding a good estimate and high power. Fourth, the most problematic pairs are $Y_1 \to Y_3$ and $Y_3 \to Y_2$ (true MR effect $\theta_{31} = \theta_{23} = 0.025$). For $Y_1 \to Y_3$, the candidate IV set includes SNPs from both $\boldsymbol{Z}_1$ and $\boldsymbol{Z}_3$ that are genome-wide significant for Y_1, but the SNPs in $\boldsymbol{Z}_3$ directly affect Y_3 and are therefore invalid IVs. Because a large proportion of the candidate IV set consists of such invalid IVs, MR-PRESSO cannot reliably identify them, leading to severely biased estimates. The same phenomenon occurs for $Y_3 \to Y_2$, where SNPs in $\boldsymbol{Z}_2$ are selected as IVs for Y_3 but directly affect Y_2.

These results highlight two fundamental limitations of conventional UVMR methods in the network setting. First, standard IV selection based on genome-wide significance for the exposure cannot distinguish between SNPs that directly affect the exposure and those that reach significance through indirect paths in the causal network, leading to systematic inclusion of invalid IVs for certain exposure–outcome pairs. This is precisely the issue that the multi-trait IV screening strategies (ScreenMax and ScreenAug) in MR2G are designed to address. Second, even when all IVs are valid, UVMR methods estimate MR effects θ_{ij} that conflate direct and indirect causal effects, making it impossible to recover the direct-effect structure $\mathbf{G}$ without the additional network recovery step employed by MR2G.

3.4 Additional analyses for the simulation with real GWAS data

3.4.1 Scaling the direct-effect graph

In the simulation with real GWAS data (Fig. 2C in the main text), the true direct-effect graph $\mathbf{G}$ was derived from the real GWAS summary statistics of six traits (LDL, BMI, SBP, CAD, Stroke, AF). To assess the robustness of MR2G across a range of effect-size

magnitudes, we repeated this simulation under weaker causal networks by multiplying the entire true direct-effect matrix by a scalar $\lambda \in \{0.1, 0.25, 0.5\}$. All other settings, including the GWAS summary statistic generation, were kept identical to those in the main text.

Fig. S presents the results across $\lambda \in \{0.1, 0.25, 0.5\}$. Overall, the results are consistent with the conclusions from the main text. When $\lambda = 0.25$ (Panel B), ScreenPair begins to exhibit inflated type-I error for CAD $\leftarrow$ Stroke, and when $\lambda = 0.5$ (Panel C), it exhibits inflated type-I error for several pathways involving Stroke. This is consistent with the phenomenon observed in the main text: as the causal effects strengthen, SNPs with direct effects on upstream risk factors (e.g., SBP) become genome-wide significant for Stroke through indirect pathways, and ScreenPair incorrectly selects them as IVs based on comparison of pairwise marginal associations. Additionally, at $\lambda = 0.25$, ScreenAug achieves higher power than ScreenMax for detecting the edges between CAD and AF, illustrating the benefit of augmenting the IV set with additional valid instruments.

3.5 Sensitivity of ScreenAug to the reference graph used for IV augmentation

ScreenAug augments the IV set by re-adding SNPs that do not have an alternative path to the outcome after removing the exposure, based on a reference graph. In the main analyses, this reference graph is estimated via ScreenMax. To assess the sensitivity of ScreenAug's performance to misspecification of the reference graph, we conducted a simulation with five traits and evaluated three alternative reference graphs.

The data-generating setup follows Section 3.3.1 with $p = 5$ traits, $m = 60$ SNPs, sample size $N = 200{,}000$, and the direct-effect matrix $\mathbf{G} = \begin{pmatrix} 0 & 0.2 & 0.2 & 0 & 0 \\ 0 & 0 & 0 & 0 & 0.2 \\ 0 & 0.1 & 0 & 0 & 0 \\ 0.03 & 0 & 0 & 0 & 0.1 \\ 0 & 0 & 0 & 0 & 0 \end{pmatrix}$. The genetic effect matrix $\mathbf{\Gamma} \in \mathbb{R}^{60\times 5}$ was block-structured: the first four traits $Y_1, \ldots, Y_4$ were each directly affected by 10 non-overlapping SNPs ($|\boldsymbol{Z}_1| = |\boldsymbol{Z}_2| = |\boldsymbol{Z}_3| = |\boldsymbol{Z}_4| = 10$) with effect sizes drawn from Uniform$(0.1, 0.2)$, while Y_5 was directly affected by the remaining 20 SNPs ($|\boldsymbol{Z}_5| = 20$). All other settings were the same as in Section 3.3.1.

Alternative reference graphs for ScreenAug. Recall that ScreenAug starts from the ScreenMax IV set $\mathcal{M}$ and re-adds SNPs from ScreenPair IV set $\mathcal{P}$ whose inclusion is supported by a reference graph: a SNP is re-included as an IV for an exposure–outcome pair only if the reference graph contains no alternative path from the SNP to the outcome after removing the exposure (see Section 4.2.2 in the main text). Beside using the ScreenMax-estimated graph as the reference graph, we considered three alternative reference graphs to evaluate the sensitivity of ScreenAug to misspecification of the reference graph:

- **ScreenAug1**: The reference graph was a randomly misspecified graph with 6 directed edges sampled uniformly at random from all $p(p-1)$ possible edges.
- **ScreenAug2**: The reference graph contained four of the six true edges, $Y_2 \to Y_1$, $Y_3 \to Y_1$, $Y_5 \to Y_2$, $Y_1 \to Y_4$, but omitted the two true edges $Y_5 \to Y_4$ and $Y_2 \to Y_3$.
- **ScreenAug3**: The reference graph contained only the two omitted edges from G2, i.e., $Y_5 \to Y_4$ and $Y_2 \to Y_3$.

Fig. T presents the results of the five-trait simulation. Panel **A** displays the true direct-effect graph and panel **B** shows the proportion of rejections across 400 replicates for MR2G coupled with ScreenAug, ScreenMax, and ScreenPair. ScreenAug and ScreenMax both maintain well-controlled type-I error across all null pairs and achieve high power for all six true edges. In contrast, ScreenPair exhibits a severely inflated type-I error for $Y_2 \to Y_4$, and fails to detect the true edge $Y_5 \to Y_4$. The inflation for $Y_2 \to Y_4$ is driven by SNPs in $\boldsymbol{Z}_5$: these variants are associated with both Y_2 and Y_4 via $Y_5 \to Y_2$ and $Y_5 \to Y_4$. In the pairwise comparison (Y_2, Y_4), the association with Y_2 is stronger ($g_{25} = 0.2 > g_{45} = 0.1$), so ScreenPair assigns many $\boldsymbol{Z}_5$ SNPs to Y_2 and uses them as IVs for $Y_2 \to Y_4$, even though they have an alternative path to Y_4 through Y_5. Moreover, the complete loss of power for $Y_5 \to Y_4$ under ScreenPair is a downstream consequence of this false detection. Although ScreenPair can correctly estimate the MR effect θ_{45} (Fig. TC, since SNPs in $\boldsymbol{Z}_5$ are correctly assigned to Y_5 in the pairwise comparison with Y_4), the spurious edge $Y_2 \to Y_4$ absorbs the indirect effect $Y_5 \to Y_2 \to Y_4$ during the

network recovery step. This causes the estimated direct effect $\hat{g}_{45}$ to shrink toward zero, effectively explaining away the true $Y_5 \to Y_4$ edge through the falsely detected $Y_2 \to Y_4$ pathway. Similarly, the reduced power for $Y_2 \to Y_3$ under ScreenPair can be attributed to a small spurious MR effect estimate for $Y_1 \to Y_3$ (Panel C), which allows part of the $Y_2 \to Y_3$ signal to be explained through the indirect path $Y_2 \to Y_1 \to Y_3$ during the network recovery step, thereby attenuating $\hat{g}_{32}$ and reducing power.

Fig. U summarizes the sensitivity of ScreenAug to the reference graph used in the augmentation step. As discussed above, the difficult pairs in this five-trait design are those embedded in confounder-like motifs, in particular $Y_4 \leftarrow Y_5 \to Y_2$ (driving spurious $Y_2 \to Y_4$) and $Y_1 \leftarrow Y_2 \to Y_3$ (which can attenuate $Y_2 \to Y_3$). In ScreenAug1 (Panel A), the reference graph is randomly generated and happens to contain a path from Y_5 to Y_4 (via $Y_5 \to Y_3 \to Y_4$). As a result, after removing Y_2, SNPs in $\boldsymbol{Z}_5$ still have a path to Y_4 in the reference graph, and ScreenAug does not re-add them as IVs for $Y_2 \to Y_4$ and type-I error remains well-controlled. However, the random graph does not contain $Y_2 \to Y_3$, so when inferring $Y_1 \to Y_3$, SNPs in $\boldsymbol{Z}_2$ have no recognized path to Y_3 after removing Y_1 and are incorrectly re-added as IVs. This introduces a small spurious $Y_1 \to Y_3$ signal that attenuates $\hat{g}_{32}$ and reduces power for $Y_2 \to Y_3$.

In ScreenAug2 (Panel B), the reference graph contains four of the six true edges but omits $Y_5 \to Y_4$ and $Y_2 \to Y_3$. Without $Y_5 \to Y_4$, after removing Y_2, SNPs in $\boldsymbol{Z}_5$ have no recognized path to Y_4, so ScreenAug re-adds them as IVs for $Y_2 \to Y_4$, leading to inflated type-I error and loss of power for $Y_5 \to Y_4$ — the same failure mode as ScreenPair. Similarly, without $Y_2 \to Y_3$, SNPs in $\boldsymbol{Z}_2$ are incorrectly re-added for $Y_1 \to Y_3$, which attenuates the $Y_2 \to Y_3$ estimate.

In ScreenAug3 (Panel C), the reference graph contains *only* the two edges $Y_5 \to Y_4$ and $Y_2 \to Y_3$. Despite being highly incomplete, these two edges are exactly what is needed: $Y_5 \to Y_4$ prevents $\boldsymbol{Z}_5$ SNPs from being re-added for $Y_2 \to Y_4$, and $Y_2 \to Y_3$ prevents $\boldsymbol{Z}_2$ SNPs from being re-added for $Y_1 \to Y_3$. As a result, type-I error is well-controlled and power matches that of ScreenAug with the ScreenMax-estimated reference graph.

Overall, ScreenAug is robust to moderate reference-graph misspecification, but it relies

on correctly capturing a small set of edges, especially those involved in the confounder-like motifs. In practice, the ScreenMax-estimated graph serves as a reasonable default. Alternatively, if prior knowledge about the causal network is available (e.g., known confounder-like motifs from domain expertise or previous studies), users can directly supply a reference graph to ScreenAug, bypassing the ScreenMax estimation step entirely.

3.6 Computational efficiency

In MR2G, once a set of IVs is specified, constructing the directed trait network mainly involves performing pairwise MRcML analyses for all $T(T-1)$ ordered trait pairs, followed by the network recovery algorithm (Algorithm 1 in the main text). The network recovery step itself is computationally inexpensive, as it only involves operations on a $T \times T$ matrix. In addition, the ScreenAug IV augmentation step only involves simple graph reachability checks on a graph with at most $T-1$ nodes and therefore takes negligible time.

The major computational cost arises from the pairwise MRcML runs. For a given IV set, constructing the network requires $T(T-1)$ MR analyses, which scales quadratically with the number of traits T. When B perturbation replicates are used for inference, the total number of MRcML runs becomes $B \times T(T-1)$. Importantly, the number of MR analyses depends primarily on the number of traits T and perturbation replicates B, and is largely insensitive to the underlying network structure. Within each MRcML run, the computational cost is approximately linear in the number of candidate SNPs, as the method performs invalid-IV detection and selection among these SNPs. However, the MRcML analyses across trait pairs and perturbation replicates are independent and can be easily parallelized, allowing substantial improvement in computational efficiency in practice.

To give a concrete picture of the computational efficiency, we recorded the runtime of the main computational steps under several simulation settings for ScreenMax (Table C). All runtime measurements were obtained on a 36-core computing node, with parallelization of data perturbation implemented using `pbmclapply` in R. The runtime for the network recovery step (Algorithm 1) was negligible (less than 0.1 seconds on average)

across all settings.

References

[1] Zhaotong Lin, Haoran Xue, and Wei Pan. Combining mendelian randomization and network deconvolution for inference of causal networks with gwas summary data. *PLoS genetics*, 19(5):e1010762, 2023.

[2] Cristen J Willer, Ellen M Schmidt, Sebanti Sengupta, Gina M Peloso, Stefan Gustafsson, Stavroula Kanoni, Andrea Ganna, Jin Chen, Martin L Buchkovich, Samia Mora, et al. Discovery and refinement of loci associated with lipid levels. *Nature genetics*, 45(11):1274, 2013.

[3] Andrew R Wood, Tonu Esko, Jian Yang, Sailaja Vedantam, Tune H Pers, Stefan Gustafsson, Audrey Y Chu, Karol Estrada, Zoltán Kutalik, Najaf Amin, et al. Defining the role of common variation in the genomic and biological architecture of adult human height. *Nature genetics*, 46(11):1173–1186, 2014.

[4] Adam E Locke, Bratati Kahali, Sonja I Berndt, Anne E Justice, Tune H Pers, Felix R Day, Corey Powell, Sailaja Vedantam, Martin L Buchkovich, Jian Yang, et al. Genetic studies of body mass index yield new insights for obesity biology. *Nature*, 518(7538):197–206, 2015.

[5] GWAS result from Neale Lab. http://www.nealelab.is/uk-biobank/.

[6] Josée Dupuis, Claudia Langenberg, Inga Prokopenko, Richa Saxena, Nicole Soranzo, Anne U Jackson, Eleanor Wheeler, Nicole L Glazer, Nabila Bouatia-Naji, Anna L Gloyn, et al. New genetic loci implicated in fasting glucose homeostasis and their impact on type 2 diabetes risk. *Nature genetics*, 42(2):105–116, 2010.

[7] Mengzhen Liu, Yu Jiang, Robbee Wedow, Yue Li, David M Brazel, Fang Chen, Gargi Datta, Jose Davila-Velderrain, Daniel McGuire, Chao Tian, et al. Association

studies of up to 1.2 million individuals yield new insights into the genetic etiology of tobacco and alcohol use. *Nature genetics*, 51(2):237–244, 2019.

[8] Pim van der Harst and Niek Verweij. Identification of 64 novel genetic loci provides an expanded view on the genetic architecture of coronary artery disease. *Circulation research*, 122(3):433–443, 2018.

[9] Rainer Malik, Ganesh Chauhan, Matthew Traylor, Muralidharan Sargurupremraj, Yukinori Okada, Aniket Mishra, Loes Rutten-Jacobs, Anne-Katrin Giese, Sander W Van Der Laan, Solveig Gretarsdottir, et al. Multiancestry genome-wide association study of 520,000 subjects identifies 32 loci associated with stroke and stroke subtypes. *Nature genetics*, 50(4):524–537, 2018.

[10] Andrew P Morris, Benjamin F Voight, Tanya M Teslovich, Teresa Ferreira, Ayellet V Segre, Valgerdur Steinthorsdottir, Rona J Strawbridge, Hassan Khan, Harald Grallert, Anubha Mahajan, et al. Large-scale association analysis provides insights into the genetic architecture and pathophysiology of type 2 diabetes. *Nature genetics*, 44(9):981, 2012.

[11] Florence Demenais, Patricia Margaritte-Jeannin, Kathleen C Barnes, William OC Cookson, Janine Altmüller, Wei Ang, R Graham Barr, Terri H Beaty, Allan B Becker, John Beilby, et al. Multiancestry association study identifies new asthma risk loci that colocalize with immune-cell enhancer marks. *Nature genetics*, 50(1):42–53, 2018.

[12] Jonas B Nielsen, Rosa B Thorolfsdottir, Lars G Fritsche, Wei Zhou, Morten W Skov, Sarah E Graham, Todd J Herron, Shane McCarthy, Ellen M Schmidt, Gardar Sveinbjornsson, et al. Biobank-driven genomic discovery yields new insight into atrial fibrillation biology. *Nature genetics*, 50(9):1234–1239, 2018.

[13] Iris E Jansen, Jeanne E Savage, Kyoko Watanabe, Julien Bryois, Dylan M Williams, Stacy Steinberg, Julia Sealock, Ida K Karlsson, Sara Hägg, Lavinia Athanasiu, et al.

Genome-wide meta-analysis identifies new loci and functional pathways influencing alzheimer's disease risk. *Nature genetics*, 51(3):404–413, 2019.

[14] Haoran Xue, Xiaotong Shen, and Wei Pan. Constrained maximum likelihood-based mendelian randomization robust to both correlated and uncorrelated pleiotropic effects. *The American Journal of Human Genetics*, 108(7):1251–1269, 2021.

[15] James E Gentle. Matrix algebra. *Springer texts in statistics, Springer, New York, NY, doi*, 10:978–0, 2007.

[16] Dennis D Boos and Leonard A Stefanski. *Essential statistical inference: theory and methods*, volume 591. Springer, 2013.

[17] Stephen Burgess, Adam Butterworth, and Simon G Thompson. Mendelian randomization analysis with multiple genetic variants using summarized data. *Genetic epidemiology*, 37(7):658–665, 2013.

[18] Jack Bowden, George Davey Smith, and Stephen Burgess. Mendelian randomization with invalid instruments: effect estimation and bias detection through egger regression. *International journal of epidemiology*, 44(2):512–525, 2015.

[19] Marie Verbanck, Chia-Yen Chen, Benjamin Neale, and Ron Do. Detection of widespread horizontal pleiotropy in causal relationships inferred from mendelian randomization between complex traits and diseases. *Nature genetics*, 50(5):693–698, 2018.

[20] Zhaotong Lin, Isaac Pan, and Wei Pan. A practical problem with egger regression in mendelian randomization. *PLoS genetics*, 18(5):e1010166, 2022.
